# FBXL4 suppresses mitophagy by restricting the accumulation of NIX and BNIP3 mitophagy receptors

**DOI:** 10.15252/embj.2022112767

**Published:** 2023-05-10

**Authors:** Giang Thanh Nguyen‐Dien, Keri‐Lyn Kozul, Yi Cui, Brendan Townsend, Prajakta Gosavi Kulkarni, Soo Siang Ooi, Antonio Marzio, Nissa Carrodus, Steven Zuryn, Michele Pagano, Robert G Parton, Michael Lazarou, S Sean Millard, Robert W Taylor, Brett M Collins, Mathew JK Jones, Julia K Pagan

**Affiliations:** ^1^ Faculty of Medicine, School of Biomedical Sciences University of Queensland Brisbane QLD Australia; ^2^ Department of Biotechnology, School of Biotechnology Viet Nam National University‐International University Ho Chi Minh City Vietnam; ^3^ Department of Biochemistry and Molecular Pharmacology New York University Grossman School of Medicine New York NY USA; ^4^ Perlmutter Cancer Center New York University Grossman School of Medicine New York NY USA; ^5^ Department of Pathology and Lab Medicine, Meyer Cancer Center Weill Cornell Medicine New York NY USA; ^6^ Clem Jones Centre for Ageing Dementia Research, Queensland Brain Institute The University of Queensland Brisbane QLD Australia; ^7^ Institute for Molecular Bioscience The University of Queensland Brisbane QLD Australia; ^8^ Centre for Microscopy and Microanalysis University of Queensland Brisbane QLD Australia; ^9^ Walter and Eliza Hall Institute of Medical Research Parkville VIC Australia; ^10^ Department of Biochemistry and Molecular Biology, Biomedicine Discovery Institute Monash University Melbourne VIC Australia; ^11^ Department of Medical Biology University of Melbourne Melbourne VIC Australia; ^12^ Wellcome Centre for Mitochondrial Research, Translational and Clinical Research Institute, Faculty of Medical Sciences Newcastle University Newcastle upon Tyne UK; ^13^ NHS Highly Specialised Service for Rare Mitochondrial Disorders Newcastle upon Tyne Hospitals NHS Foundation Trust Newcastle upon Tyne UK; ^14^ The University of Queensland Diamantina Institute, Faculty of Medicine The University of Queensland Brisbane QLD Australia; ^15^ School of Chemistry & Molecular Biosciences University of Queensland Brisbane QLD Australia

**Keywords:** BNIP3, FBXL4, mitochondria, mitophagy, NIX/BNIP3L, Autophagy & Cell Death, Organelles, Post-translational Modifications & Proteolysis

## Abstract

To maintain both mitochondrial quality and quantity, cells selectively remove damaged or excessive mitochondria through mitophagy, which is a specialised form of autophagy. Mitophagy is induced in response to diverse conditions, including hypoxia, cellular differentiation and mitochondrial damage. However, the mechanisms that govern the removal of specific dysfunctional mitochondria under steady‐state conditions to fine‐tune mitochondrial content are not well understood. Here, we report that SCF^FBXL4^, an SKP1/CUL1/F‐box protein ubiquitin ligase complex, localises to the mitochondrial outer membrane in unstressed cells and mediates the constitutive ubiquitylation and degradation of the mitophagy receptors NIX and BNIP3 to suppress basal levels of mitophagy. We demonstrate that the pathogenic variants of FBXL4 that cause encephalopathic mtDNA depletion syndrome (MTDPS13) do not efficiently interact with the core SCF ubiquitin ligase machinery or mediate the degradation of NIX and BNIP3. Thus, we reveal a molecular mechanism whereby FBXL4 actively suppresses mitophagy by preventing NIX and BNIP3 accumulation. We propose that the dysregulation of NIX and BNIP3 turnover causes excessive basal mitophagy in FBXL4‐associated mtDNA depletion syndrome.

## Graphical abstract

Steady‐state ubiquitylation and turnover of NIX and BNIP3 limits basal mitophagy and is dysregulated in MTDPS13, accounting for the elevated mitophagy associated with this encephalopathic mtDNA depletion syndrome.

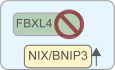

## Introduction

Mitophagy, also known as mitochondrial autophagy, is a process where surplus, aged or damaged mitochondria are selectively degraded through autophagy (Pickles *et al*, 2018; Onishi *et al*, 2021). It involves the engulfment of mitochondria in a double‐membrane vesicle called an autophagosome, which then fuses with lysosomes for degradation. Diverse mechanisms trigger mitophagy in response to various mitochondrial stressors or physiological signals, such as mitochondrial membrane depolarisation (Narendra *et al*, 2008; Jin *et al*, 2010), hypoxia (Sowter *et al*, 2001; Bellot *et al*, 2009; Allen *et al*, 2013) or cellular differentiation (Schweers *et al*, 2007; Sandoval *et al*, 2008; Esteban‐Martinez *et al*, 2017; Simpson *et al*, 2021). In addition, it is increasingly recognised that mitophagy occurs under basal conditions (i.e. in the absence of induced mitochondrial damage) (Lee *et al*, 2018; McWilliams *et al*, 2018). In contrast to stimulus‐induced mitophagy, the mechanisms by which cells regulate mitophagy at steady state are not understood.

Mitophagy is initiated through specific signals on the mitochondrial outer membrane that act as docking sites for the nascent autophagosome. In the case of mitochondrial membrane depolarisation, Pink1 and Parkin proteins cooperate to induce the ubiquitylation of outer membrane proteins, which indirectly induce autophagosome formation via autophagy adaptors that bind to ubiquitin (Lazarou *et al*, 2015). In contrast, in response to hypoxia or developmental signals, mitochondrial outer membrane proteins NIX and BNIP3 are upregulated and act as direct receptors for the autophagosome (Schweers *et al*, 2007; Sandoval *et al*, 2008; Zhang *et al*, 2008; Bellot *et al*, 2009; Novak *et al*, 2010; Esteban‐Martinez *et al*, 2017; Simpson *et al*, 2021). NIX and BNIP3 proteins share approximately 50% homology and structural features, including an atypical BH3 domain, a C‐terminal transmembrane domain necessary for mitochondrial outer membrane localisation and LC3‐interaction motifs (LIR) that face the cytoplasm, facilitating the recruitment of LC3 proteins on the autophagosome (Novak *et al*, 2010; Hanna *et al*, 2012). Under normal conditions, NIX and BNIP3 proteins are barely detectable on the outer mitochondrial membrane, but in response to hypoxia or iron chelation, they are upregulated through HIF1α‐mediated transcription to mediate mitophagy (Sowter *et al*, 2001; Allen *et al*, 2013; Zhao *et al*, 2020).

Cullin‐RING ligases (CRLs) comprise the largest family of multi‐subunit E3 ligases (Lydeard *et al*, 2013; Harper & Schulman, 2021). Each CRL complex contains one of eight different Cullin subunits, which act as assembly scaffolds, binding at their C‐termini to a RING finger protein (RBX1 or RBX2), which is required for binding to the E2 ubiquitin conjugating enzyme. To recognise specific substrates, each CRL complex binds to adaptor proteins which recruit variable substrate recognition proteins at their N‐termini. The SCF (SKP1–CUL1–F‐box protein) sub‐family of CRLs (also known as CRL1 complexes) consist of the CUL1 backbone, the RBX1 RING subunit, the adaptor protein SKP1 and one of 69 different F‐box proteins in humans as a substrate‐binding component (Skaar *et al*, 2013; Duan & Pagano, 2021), one of which is the mitochondria‐localised F‐box protein, FBXL4.

In humans, pathogenic, bi‐allelic *FBXL4* variants result in encephalopathic mitochondrial DNA (mtDNA) depletion syndrome (MTDPS13) (Bonnen *et al*, 2013; Gai *et al*, 2013; Ballout *et al*, 2019), a multi‐system disease that presents with congenital lactic acidosis, neurodevelopmental delays, poor growth and encephalopathy (Bonnen *et al*, 2013; Gai *et al*, 2013). FBXL4‐deficiency leads to severe oxidative phosphorylation deficiency correlating with a quantitative loss of mtDNA copy number (mtDNA depletion), hyper‐fragmentation of the mitochondrial network and diminished steady‐state levels of mitochondrial proteins (Bonnen *et al*, 2013; Gai *et al*, 2013; Ballout *et al*, 2019; Sabouny *et al*, 2019; Alsina *et al*, 2020). Despite the serious consequences of FBXL4 deficiency, no mitochondrial substrates for FBXL4 have yet been identified.

Here, we report a mechanism whereby SCF‐FBXL4 constitutively targets the mitophagy receptors NIX and BNIP3 for degradation, restricting steady‐state mitophagy. We found that MTDPS13‐associated pathogenic variants of FBXL4 are unable to efficiently mediate NIX and BNIP3 degradation. Our results suggest that the increased basal mitophagy and associated molecular phenotypes in FBXL4‐associated mtDNA depletion syndrome are caused by NIX and BNIP3 hyperaccumulation.

## Results

### Identification of FBXL4 as a suppresser of NIX and BNIP3 levels

HIF1α is the master regulator of hypoxia‐ and iron chelation‐induced mitophagy via transcriptional upregulation of NIX and BNIP3 mitophagy receptors (Sowter *et al*, 2001; Allen *et al*, 2013; Zhao *et al*, 2020). This pathway is antagonised by the activity of the CRL2‐VHL ubiquitin ligase, which mediates the polyubiquitylation and proteolytic degradation of HIF1α (Maxwell *et al*, 1999; Ivan *et al*, 2001; Jaakkola *et al*, 2001), thus suppressing mitophagy by preventing both HIF1α stabilisation and the consequent upregulation of NIX and BNIP3. We investigated whether, in addition to CRL2‐VHL, any other CRLs suppress mitophagy, possibly through targeting NIX and BNIP3 for degradation directly. Mitophagy was assessed using the pH‐dependent mito‐Keima (mt‐Keima) reporter and confocal microscopy to detect mito‐lysosomes (Sun *et al*, 2017). To ensure that the contribution of Parkin‐dependent mitophagy was excluded, mitophagy assays were conducted using U2OS or HeLa cells, with low or no Parkin expression respectively (Tang *et al*, 2017; Munson *et al*, 2022).

**Figure 1:**
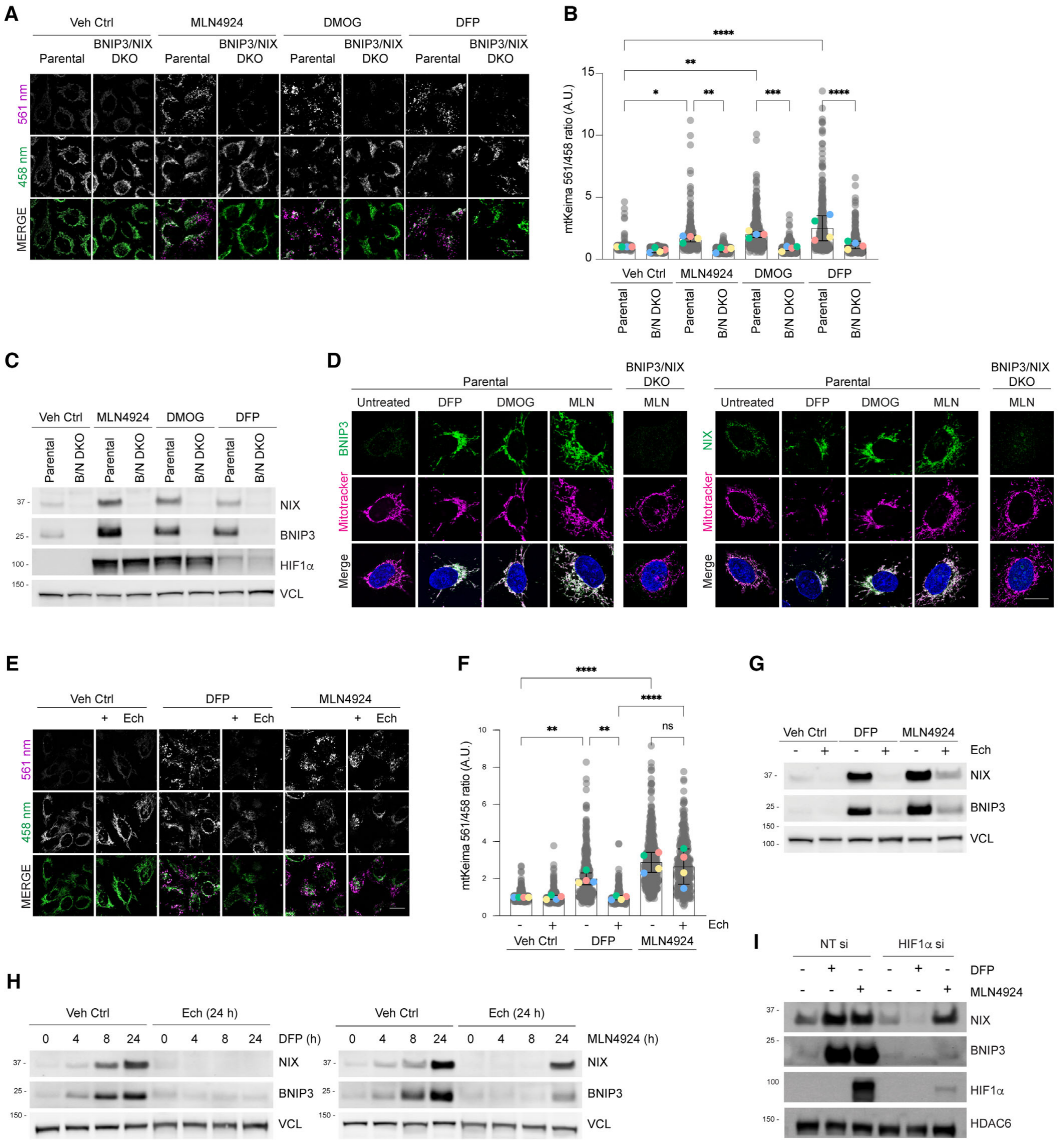
Cullin‐RING ligases suppress mitophagy and NIX/BNIP3 levels independently of HIF1α *NIX and BNIP3 are required for mitophagy induced by MLN4924 and other HIF1α stabilisers*. Parental HeLa cells and NIX/BNIP3 double‐knockout (NIX/BNIP3 DKO) HeLa cells expressing mt‐Keima were treated with DFP (1 mM), MLN4924 (0.5 μM) or DMOG (10 nM) for 24 h and analysed by live‐cell confocal microscopy. The emission signals obtained after excitation with the 458 nm laser (neutral pH) or 561 nm laser (acidic pH) are shown in green and magenta respectively.
*Quantification of mitophagy shown in panel A*. Mitophagy is represented as the ratio of mt‐Keima 561 nm fluorescence intensity to mt‐Keima 458 nm fluorescence intensity for individual cells normalised to the mean of the untreated condition.
*Analysis of NIX and BNIP3 protein levels after MLN4924*, *DMOG or DFP treatments*. HeLa cells or HeLa NIX/BNIP3 DKO cells were treated with MLN4924, DMOG or DFP for 24 h. Total‐cell lysates corresponding to conditions in panel A were subject to immunoblotting.
*NIX and BNIP3 accumulate on mitochondria in response to MLN4924*, *DMOG or DFP treatments*. HeLa cells or HeLa NIX/BNIP3 DKO cells were treated as in panel A, fixed and stained with the indicated antibodies and MitoTracker.
*Inhibition of HIF1α with echinomycin prevents DFP‐induced mitophagy but not MLN4924‐induced mitophagy*. U2OS cells expressing mt‐Keima were treated with the indicated drugs for 24 h and analysed by live‐cell confocal microscopy.Quantification of mitophagy shown in panel D.Analysis of NIX and BNIP3 levels after DFP or MLN4924 treatments when HIF1α is inhibited using echinomycin. Total‐cell lysates corresponding to conditions in panel E‐F were subject to immunoblotting.
*Inhibition of HIF1*α *with echinomycin prevents the increase of NIX and BNIP3 in response to DFP*, *but not MLN4924*. U2OS cells were treated with DFP or MLN4924 over a time course. Where indicated, cells were also treated for 24 h with echinomycin. Total‐cell lysates were subject to immunoblotting as shown.
*Depletion of HIF1α by siRNA prevents the increase of NIX and BNIP3 in response to DFP*, *but not in response to MLN4924*. U2OS cells were transfected with non‐targeting siRNAs (NT si) or siRNAs targeting HIF1α. Cells were treated with DFP or MLN4924 for 24 h prior to harvesting for immunoblotting. Data information: In (B and F), the measurements from individual cells are represented as translucent grey dots, and the mean ratio from each independent experiment is represented by coloured circles. The centre lines and bars depict the mean of the averaged independent replicates ± SD. *N* = 4. A minimum of 50 cells were analysed for each condition within each individual replicate experiment, and over 300 cells were analysed for each condition in total. *P* values were calculated based on the mean ratio values from independent experiments using one‐way ANOVA (*P < 0.05, **P < 0.005, ***P < 0.001, ****P < 0.0001). Scale bars = 20 μm. Source data are available online for this figure.

First, we inhibited the entire CRL‐ubiquitin ligase family using MLN4924, an inhibitor of Cullin neddylation (Soucy *et al*, 2009). We observed robust mitophagy (Fig 1A and B), along with an increase in NIX and BNIP3 levels (Fig 1C and D), similar to the effects seen with other HIF1α stabilisers like the iron chelator deferiprone (DFP) and prolyl hydroxylase inhibitor dimethyloxalylglycine (DMOG) (Allen *et al*, 2013). We used CRISPR–Cas9 to create NIX/BNIP3 double‐knockout cells to investigate whether NIX and BNIP3 are essential for mitophagy in response to MLN4924, as is the case for mitophagy triggered by DFP (Zhao *et al*, 2020; Wilhelm *et al*, 2022). We observed that mitophagy was diminished in the absence of NIX and BNIP3 in response to MLN4924, as well as the other HIF1α activators DMOG and DFP (Fig 1A and B).

To investigate whether a different Cullin‐RING ligase besides CRL2‐VHL is involved in regulating mitophagy and NIX/BNIP3 protein levels, we treated cells with MLN4924 and inhibited the effect of HIF1α transcription on NIX and BNIP3 protein levels using echinomycin, which is a HIF1α inhibitor (Kong *et al*, 2005). As anticipated from prior studies (Zhao *et al*, 2020), inhibiting HIF1α activity with echinomycin resulted in the inhibition of DFP‐induced mitophagy (Fig 1E and F) and DFP‐induced upregulation of NIX and BNIP3 (Fig 1G and H). However, we observed that MLN4924‐induced mitophagy was only partially eliminated by echinomycin (Fig 1D and E), demonstrating that one or more CRL(s) suppress mitophagy via a HIF1α‐independent mechanism. Similarly, we found that echinomycin or HIF1α siRNA only partially prevented the upregulation of NIX and BNIP3 protein levels in response to MLN4924 (Fig 1G–I), suggesting that an additional CRL is involved in the regulation of NIX and BNIP3 protein levels via a HIF1α‐independent mechanism. Taken together, these findings suggest that a CRL‐based mechanism restricts mitophagy under basal conditions in cells, in a HIF1α‐independent manner, possibly through post‐translational regulation of NIX/BNIP3.

**Figure EV1:**
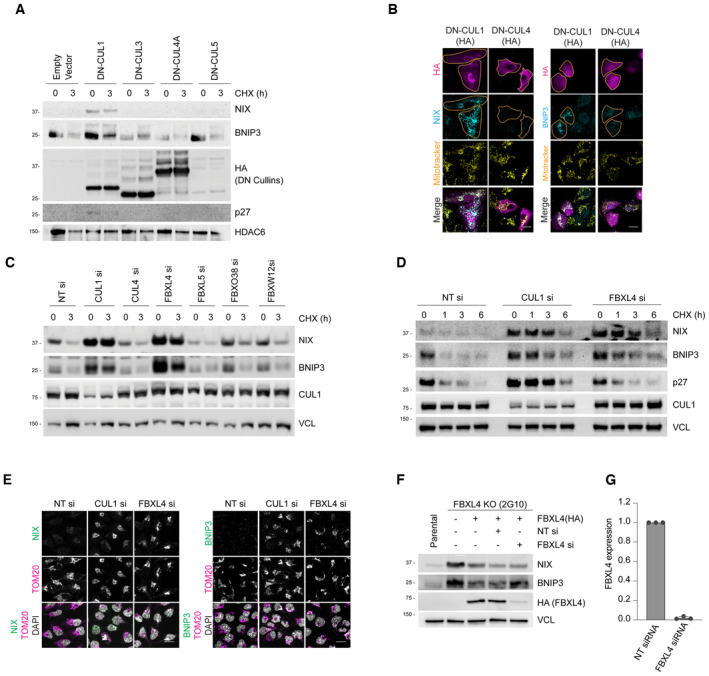
Identification of SCF‐FBXL4 as a negative regulator of NIX and BNIP3 stability *Expression of dominant‐negative* (*DN*) *Cullin 1 results in an increase in the levels and half‐life of NIX and BNIP3*. HeLa‐T‐REx‐Flp‐in cells were transfected with FLAG‐HA‐tagged dominant‐negative CUL1, CUL3, CUL4A and CUL5 or an empty vector. Cells were treated with cycloheximide for 3 h followed by immunoblotting with the indicated antibodies.
*Expression of dominant‐negative* (*DN*) *Cullin 1 results in the accumulation of NIX and BNIP3 at mitochondria*. U2OS cells were transfected with FLAG‐HA‐tagged DN‐CUL1 or FLAG‐HA‐tagged DN‐CUL4 and immunostained for both HA and either NIX or BNIP3. An orange line marks the edge of the individual cells expressing the dominant‐negative cullin protein.
*Screen for F‐box proteins required for turnover of NIX and BNIP3*. U2OS cells were transfected with the indicated siRNAs. Total‐cell lysates were subject to immunoblotting as shown. NIX and BNIP3 are stabilised by depletion of CUL1 and FBXL4 (but not other F‐box proteins). NT = non‐targeting.
*NIX and BNIP3 are upregulated and stabilised by depletion of FBXL4 and CUL1*. U2OS cells were transfected with non‐targeting siRNA, CUL1 siRNA or FBXL4 siRNA. Cells were treated with cycloheximide for the indicated times prior to immunoblotting with the specified antibodies.
*Depletion of FBXL4 and CUL1 results in NIX and BNIP3 accumulation at mitochondria*. U2OS cells were transfected with non‐targeting siRNA, CUL1 siRNA or FBXL4 siRNA. Cells were fixed and stained with the indicated antibodies.
*Evaluation of the efficacy of FBXL4 siRNA to reduce exogenous HA‐tagged FBXL4 protein levels*. U2OS FBXL4 knockout cells (which are described in Fig 2 and Table EV1) constitutively expressing FBXL4‐HA (FBXL4 tagged with C‐terminal HA) were transfected with non‐targeting siRNA or FBXL4 siRNA. Immunoblotting was performed as indicated.U2OS cells were transfected with non‐targeting siRNA or FBXL4 siRNA and the efficiency of FBXL4 siRNA was evaluated using quantitative‐PCR. Bars represent the mean ± SD from three independent transfections. Data information: Scale bars = 20 μm.

Next, to narrow down the cullin‐RING ligase family involved in turnover of NIX and BNIP3, we examined the effect of disrupting individual cullin proteins using dominant‐negative (DN) versions. Expression of DN cullin proteins interferes with the function of the respective endogenous cullin, resulting in the accumulation of their specific substrates (Emanuele *et al*, 2011; Simoneschi *et al*, 2021). We transfected dominant‐negative (DN) versions of CUL1, CUL3, CUL4A and CUL5 into cells, finding that only expression of DN‐CUL1 increased the steady‐state levels and extended the half‐lives of NIX and BNIP3 (Fig EV1A). Furthermore, cells expressing DN‐CUL1 displayed an accumulation of NIX and BNIP3 at the mitochondria when compared to either the surrounding untransfected cells or cells expressing DN‐CUL4 (Fig EV1B). These findings indicate that NIX and BNIP3 mitophagy receptors are subject to SCF‐ubiquitin ligase‐mediated turnover.

CUL1 forms the backbone of 69 distinct SCF complexes, each containing a different F‐box protein (Skaar *et al*, 2013). To identify the specific F‐box protein(s) targeting NIX and/or BNIP3 to the SCF complex, we screened a partial siRNA library targeting F‐box proteins for increased levels of NIX and BNIP3 (Fig EV1C, shows 4 of 11 tested). Of the F‐box proteins assessed, siRNA‐targeting FBXL4 resulted in the greatest upregulation of both NIX and BNIP3 protein levels. Using a cycloheximide chase assay, we observed that the silencing of either FBXL4 or CUL1 promoted the stabilisation of both NIX and BNIP3, whereas silencing of CUL4 did not (Fig EV1D). Similarly, the depletion of FBXL4 or CUL1 resulted in the upregulation of NIX and BNIP3 at mitochondria (Fig EV1E). As endogenous FBXL4 could not be detected with available antibodies, we validated the efficiency of FBXL4 siRNA in downregulating exogenous FBXL4 protein using a cell line expressing exogenous FBXL4‐HA‐C. Western blotting analysis revealed a significant reduction in FBXL4‐HA protein levels upon transfection with FBXL4 siRNA (Fig EV1F). Additionally, q‐PCR analysis confirmed a reduction in FBXL4 mRNA levels following FBXL4 siRNA transfection (Fig EV1G). The results from the siRNA screen and follow‐up experiments collectively suggest that NIX and BNIP3 mitophagy receptors are subject to turnover mediated by SCF‐FBXL4 under steady‐state conditions.

**Figure 2:**
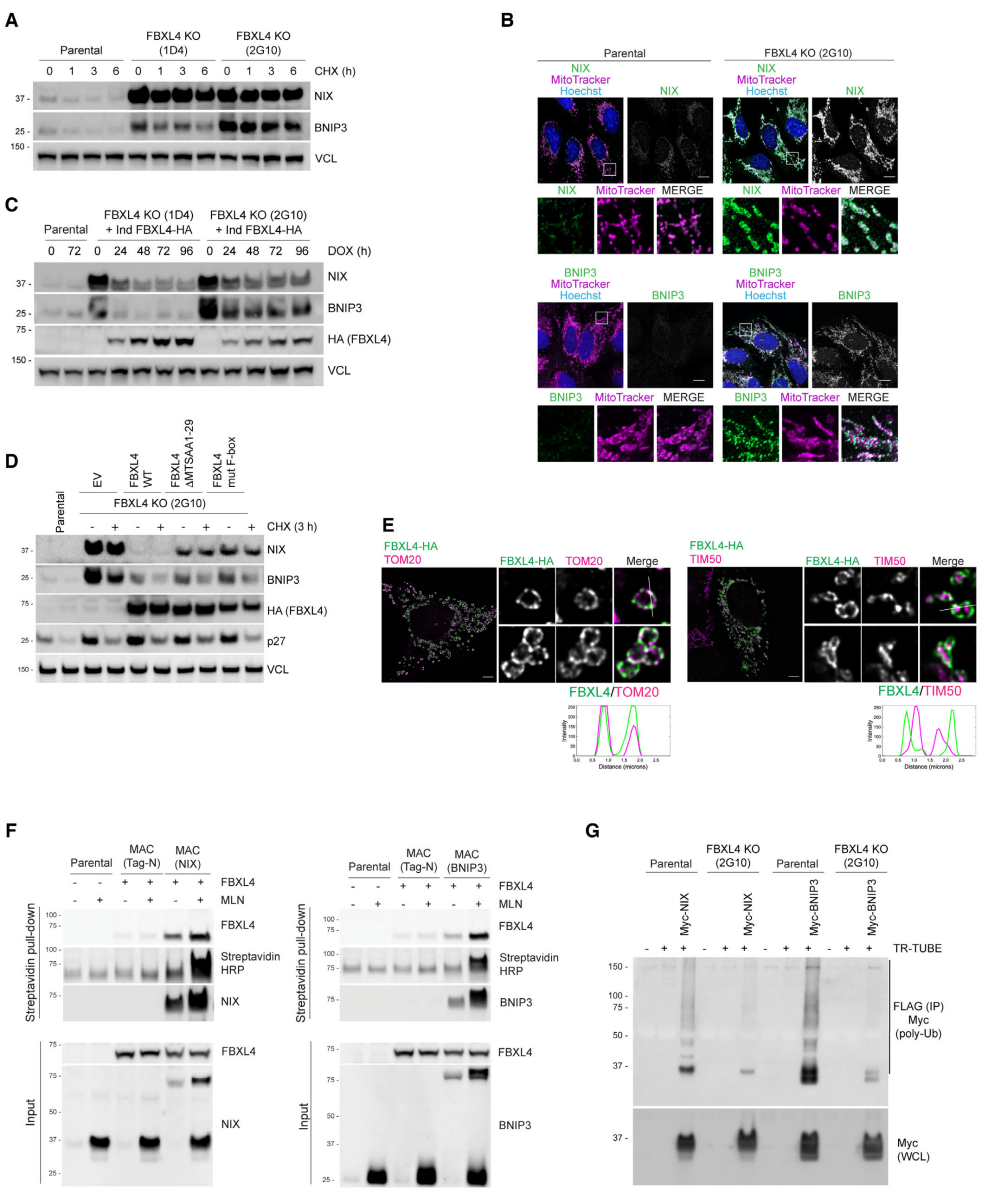
FBXL4 localises to the mitochondrial outer membrane and mediates the turnover and ubiquitylation NIX and BNIP3 *NIX and BNIP3 are upregulated and stabilised in CRISPR–Cas9 generated FBXL4‐deficient cells*. CRISPR‐mediated genome editing was used to modify the *FBXL4* locus in U2OS cells. Clonal cell lines lacking FBXL4 were treated with cycloheximide for the indicated times prior to immunoblotting.
*NIX and BNIP3 accumulate at mitochondria in FBXL4‐deficient cells*. FBXL4‐deficient cells (clone 2G10) were fixed and stained with MitoTracker (magenta) and with antibodies to NIX or BNIP3 (green). Scale bar = 10 μm.
*Re‐expression of FBXL4 into FBXL4‐defective CRISPR lines reduces the levels of NIX and BNIP3 in FBLX4‐deficient clones*. FBXL4‐deficient 2G10 and FBXL4‐deficient 1D4 cell lines were stably transduced with a doxycycline‐inducible FBXL4‐HA construct. Cells were treated with doxycycline for the indicated times prior to immunoblotting with the specified antibodies.
*FBXL4 requires its mitochondrial targeting sequence and F‐box domain to mediate NIX and BNIP3 turnover*. U2OS FBXL4 KO (2G10) cells were rescued with wild‐type FBXL4‐HA or variants lacking either the mitochondrial targeting sequence (FBXL4‐ΔMTS) or the F‐box domain (FBXL4‐F‐box mut) variants. Cells were treated with cycloheximide for 3 h prior to harvesting.
*FBXL4 localises to the mitochondrial outer membrane*. Cells transiently transfected with FBXL4‐HA‐C were treated with DFP for 24 h. Cells were stained with an anti‐HA antibody (to recognise FBXL4) and either TOM20 (an outer mitochondrial membrane protein) or TIM50 (an inner mitochondrial membrane protein). The line scan intensity profiles for FBXL4 (green) and TOM20/TIM50 (magenta) represent fluorescence intensity (*y*‐axis) plotted against distance (*x*‐axis). Scale bars = 5 μm.
*FBXL4 is a proximity interactor of NIX and BNIP3*. Cells expressing inducible BirA‐BNIP3, BirA‐NIX and BirA control were transduced with a lentiviral vector expressing FBXL4, as indicated. Cells were treated with doxycycline for 48 h (to induce BirA‐bait protein expression), biotin for 24 h (for the biotinylation reaction) and, where indicated, MLN4924 for 24 h (to stabilise NIX and BNIP3). Streptavidin‐coupled beads were used to capture the biotinylated proteins. FBXL4 was specifically detected in the eluate from BirA‐BNIP3 and BirA‐NIX compared with BirA‐alone.
*NIX and BNIP3 polyubiquitylation depend on FBXL4*. U2OS or U2OS‐FBXL4 KO cells were co‐transfected with TR‐TUBE and either myc‐BNIP3 or myc‐NIX, as indicated. Cell lysates obtained 48 h post‐transfection were immunoprecipitated with anti‐FLAG beads, and the immunoprecipitates were analysed by immunoblotting using anti‐myc antibody (to detect ubiquitylated NIX or BNIP3). The line on the right marks a ladder of bands corresponding to polyubiquitylated myc‐BNIP3 or myc‐NIX. Source data are available online for this figure.

**Figure 3:**
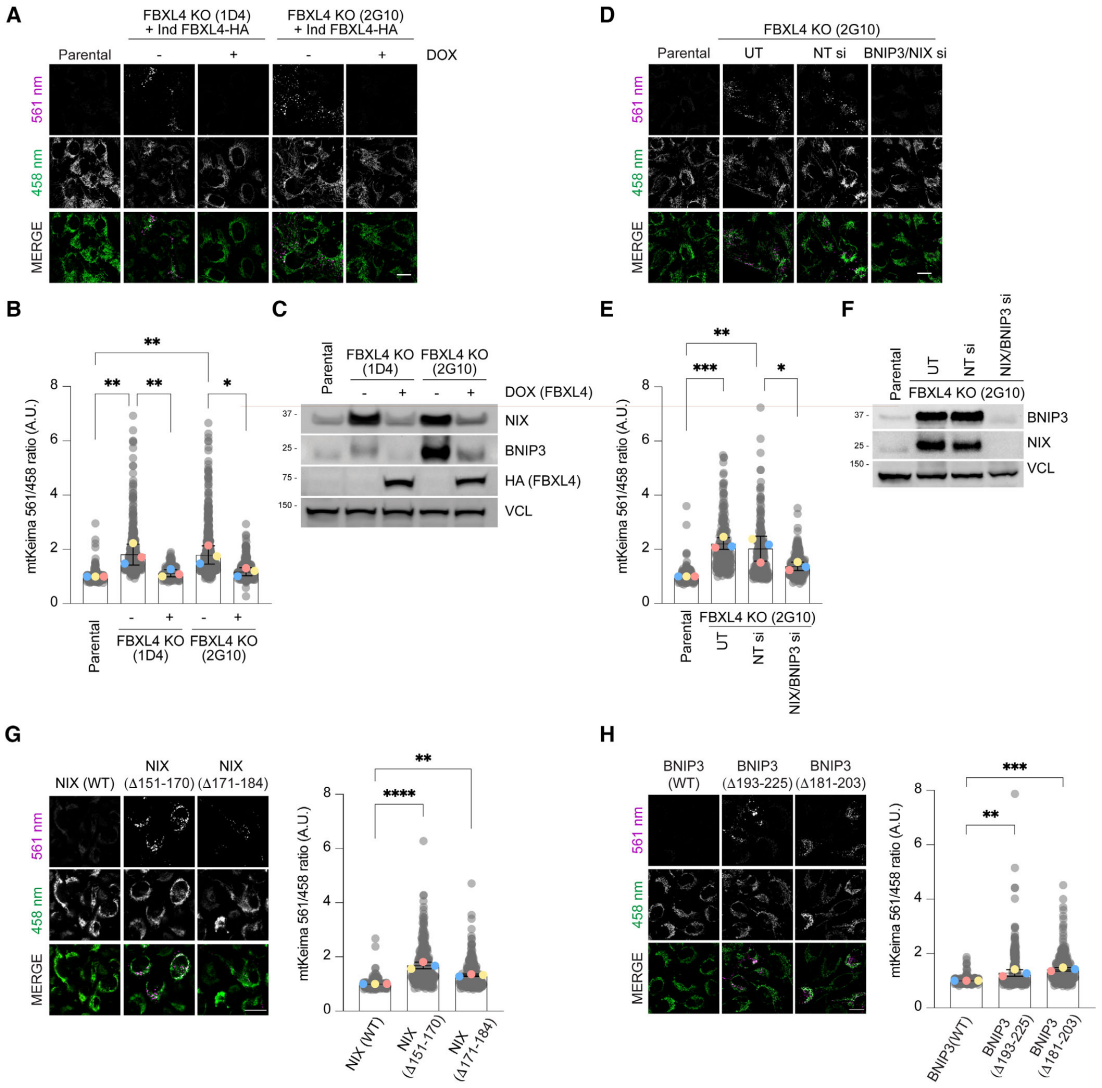
FBXL4 deficiency promotes mitophagy through NIX/BNIP3 stabilisation *Loss of FBXL4 leads to an increase in mitophagy*, *which can be reduced by re‐expression of FBXL4*. U2OS mt‐Keima FBXL4 KO clones (2G10 and 1D4) expressing doxycycline‐inducible wild‐type FBXL4‐HA were treated with doxycycline for 72 h. The emission signals obtained after excitation with the 458 nm laser (neutral pH) or 561 nm laser (acidic pH) are shown in green and magenta respectively.
*Quantification of mitophagy shown in panel A*. Mitophagy is represented as the ratio of mt‐Keima 561 nm fluorescence intensity divided by mt‐Keima 458 nm fluorescence intensity for individual cells normalised to the parental condition.Corresponding cells from (A, B) were harvested for immunoblotting to analyse the extent of NIX and BNIP3 reduction by induction of FBXL4‐HA.
*Depleting NIX/BNIP3* via *siRNA reduces the increased mitophagy caused by FBXL4 deficiency*. U2OS mt‐Keima cells and U2OS mt‐Keima FBXL4 KO 2G10 cells were transfected with siRNAs targeting both NIX and BNIP3 (NIX/BNIP3 si) or non‐targeting siRNA (NT si). UT, untransfected.Quantification of (D).Corresponding cells from (D, E) were harvested for immunoblotting to analyse the extent of NIX and BNIP3 reduction after siRNA transfection.
*Hyper‐stable NIX deletion mutants increase mitophagy compared with wild‐type NIX*. Hela Flp‐in NIX KO mt‐Keima cells stably expressing inducible NIX(WT), FLAG‐NIXΔ151‐170 or FLAG‐NIXΔ171‐184 were treated with doxycycline for 48 h (see Fig EV2B and E, top panel). Mitophagy was visualised as in (A) and quantified as in (B).
*Hyper‐stable BNIP3 deletion mutants increase mitophagy compared with wild‐type NIX*. Hela Flp‐in NIX/BNIP3 DKO mt‐Keima cells stably expressing BNIP3 deletion mutants were treated with doxycycline for 48 h (see Fig EV2C and E, bottom panel). Data information: In (B, E, G and H), translucent grey dots represent measurements from individual cells. Coloured circles represent the mean ratio from independent experiments. The centre lines and bars represent the mean of the independent replicate means ± SD. *N* = 3. At least 200 cells were analysed per condition. *P* values were calculated based on the mean values using a one‐way ANOVA (*P < 0.05, **P < 0.005, ***P < 0.001, ****P < 0.0001). Scale bars = 20 μm. Source data are available online for this figure.

To confirm that FBXL4 mediates the turnover of NIX and BNIP3, we generated FBXL4‐deficient U2OS cell lines using CRISPR/Cas9‐mediated gene disruption. Genomic sequencing analysis of the FBXL4‐deficient clones, FBXL4‐2G10 and FBXL4‐1D4, revealed a frameshift mutation resulting in an early termination codon at position Arg209 and a 5 amino acid deletion between Glu367–Glu372 respectively (Table EV1). Results from a cycloheximide chase assay demonstrated that the FBXL4‐deficient cell lines had increased stability of NIX and BNIP3 (Fig 2A), and further analysis revealed significantly higher levels of NIX and BNIP3 at mitochondria (Fig 2B). These findings support the role of FBXL4 in mediating the turnover of NIX and BNIP3. Rescue experiments demonstrated that inducible expression of FBXL4 tagged with HA at its C‐terminus (FBXL4^HA‐C^) in both FBXL4‐deficient cell lines (FBXL4‐2G10 and FBXL4‐1D4) could restore the elevated NIX and BNIP3 protein levels back to parental levels, further demonstrating that FBXL4 mediates the turnover of NIX and BNIP3 (Figs 2C and 3C). The downregulation of NIX and BNIP3 by FBXL4 was found to require FBXL4's mitochondria‐localisation sequence and a functional F‐box domain (Fig 2D), indicating that FBXL4's activity depends on its mitochondrial localisation and its interaction with SKP1 and CUL1. Thus, our results suggest that FBXL4 ubiquitin ligase mediates the turnover of NIX and BNIP3 mitophagy receptors.

### FBXL4 localises to the outer mitochondrial membrane and mediates the ubiquitylation of NIX and BNIP3

NIX and BNIP3 localise to the mitochondrial outer membrane (Fig 1D), and therefore we investigated if FBXL4 also localises to the mitochondrial outer membrane by immunofluorescence microscopy. Cells were treated with DFP to induce the formation of swollen and donut‐shaped mitochondria, which facilitated the distinction between the inner and outer mitochondrial membranes. We observed that FBXL4‐HA‐C predominantly colocalised with TOM20, an outer mitochondrial membrane protein, and was located outside of TIM50, an inner membrane protein (Fig 2E). This was supported by line scan colocalisation analysis demonstrating that there is a distinct separation between the signals of TIM50 and FBXL4, whereas the FBXL4 and TOM20 signals largely overlap. This led us to conclude that FBXL4 predominantly localises to the mitochondrial outer membrane.

To further demonstrate the spatial proximity between FBXL4 and NIX and BNIP3, we utilised proximity‐dependent biotin identification (BioID) (Fig 2F). BioID was chosen due to its usefulness in detecting weak or transient interactions before cell lysis and under denaturing conditions that allow for the solubilisation of mitochondrial membrane proteins. BioID is based on the biotin ligase BirA, which is fused to a bait protein (in this case, either NIX or BNIP3), to biotinylate prey proteins within a labelling radius of approximately 10 nm. Cells expressing inducible BirA‐tagged BNIP3 or BirA‐tagged NIX were treated with MLN4924 to inhibit the CRL‐dependent degradation of NIX/BNIP3. Immunoblot analysis of the biotinylated proteins isolated in the Streptavidin‐pulldown revealed both BirA‐BNIP3 and BirA‐NIX, but not the BirA‐alone control, associated with FBXL4‐HA‐C (Fig 2F), indicating that FBXL4 is co‐located with NIX and BNIP3.

We investigated whether FBXL4 mediates the ubiquitylation of NIX and BNIP3. To assess this, we co‐transfected myc‐tagged NIX or BNIP3 with FLAG‐tagged TR‐TUBE, which is a tandem ubiquitin‐binding entity that binds polyubiquitin chains and protects them from proteasome‐mediated degradation (Yoshida *et al*, 2015). The assay involves affinity purification of FLAG‐tagged TUBE, followed by Western blotting of the potentially ubiquitylated protein of interest to detect smears of polyubiquitylated species (in this case myc‐tagged NIX or BNIP3). Immunoprecipitation of TR‐TUBE showed a co‐precipitating smear of high‐molecular weight species detected by the myc antibody, reflecting polyubiquitylation of myc‐NIX and myc‐BNIP3. The ubiquitylated species induced by TR‐TUBE and detected using the anti‐myc antibody were dramatically reduced in the FBXL4 knockout cell line, indicating that ubiquitylation of both NIX and BNIP3 relies on the presence of FBXL4 (Fig 2G).

**Figure EV2:**
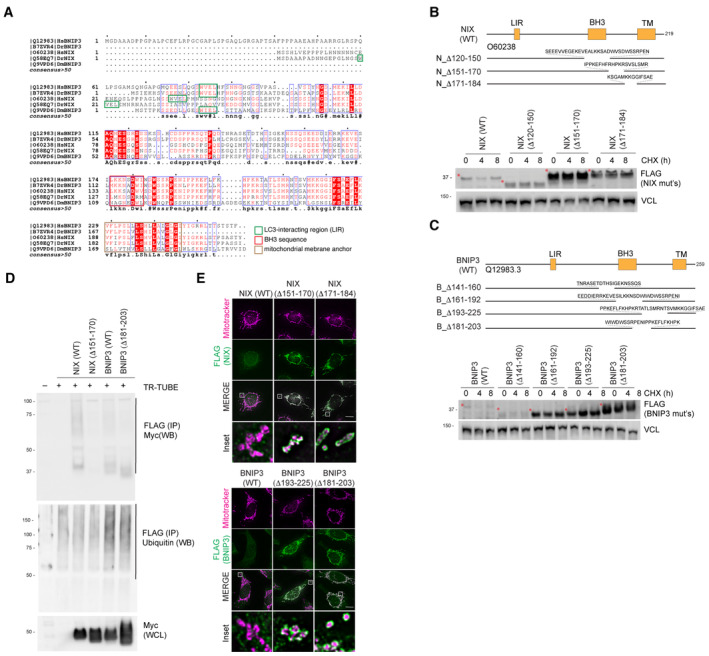
Mapping the instability regions of NIX and BNIP3 to generate hyper‐stable NIX and BNIP3 *Alignment of NIX and BNIP3 orthologues outlining conserved residues*. The LC3‐interacting region, non‐canonical BH3 region and the trans‐membrane domains are shown. The blue boxes represent regions of conservation. White letters on a red background are strictly conserved, and red letters are highly conserved.
*Deletion of regions from the C‐terminus of NIX results in its stabilisation compared with wild‐type NIX*. Hela‐Flp‐In NIX knockout cells expressing inducible FLAG‐NIX‐WT, FLAG‐NIXD120‐150, FLAG‐NIXD151‐170 and FLAG‐NIXD171‐184 were treated with cycloheximide (CHX) for the indicated times. Cells were then lysed and analysed by immunoblotting. Red asterisks denote the size of NIX or its deletion mutants.
*Deletion of regions from the C‐terminus of BNIP3 results in its stabilisation compared with wild‐type BNIP3*. HeLa Flp‐in BNIP3 knockout cells expressing inducible FLAG‐BNIP3‐WT or the specified FLAG‐BNIP3 deletions mutants were treated with CHX. Red asterisks denote the size of BNIP3 or its deletion mutants.
*Hyper‐stable NIX and BNIP3 are ubiquitylated to a lesser extent than wild‐type NIX and BNIP3*. HeLa NIX/BNIP3 DKO cells were co‐transfected with FLAG‐tagged TR‐TUBE and myc‐tagged NIX/BNIP3 constructs: NIX(WT), hyper‐stable NIXΔ151‐170, BNIP3(WT) or hyper‐stable BNIP3(Δ181‐203). FLAG‐tagged TUBE protein was affinity purified from the corresponding lysates using FLAG‐beads, and the precipitates were analysed by immunoblotting using antibodies to detect myc. The ladder of signal in the wild‐type NIX and BNIP3 lanes corresponds to polyubiquitylated myc‐BNIP3 or myc‐NIX.
*FLAG‐NIX/BNIP3 and deletion mutants localise to the mitochondria*. HeLa‐Flp‐in cells expressing inducible FLAG‐NIX/BNIP3‐WT or deletion mutants were stained for MitoTracker (magenta) and FLAG antibodies (green). Scale bars = 20 mm.

Unlike some F‐box proteins that recognise short degrons on their substrates (e.g. βTRCP), crystal structures of F‐box proteins of the LRR family (e.g. FBXL3) suggest that their interaction with substrates can occur over large surfaces (Skaar *et al*, 2013; Xing *et al*, 2013), precluding the mapping of short degron sequences that disrupt binding to FBXL4. Therefore, we aimed to identify the regions of NIX and BNIP3 required for their destabilisation by generating a series of deletion constructs for inducible expression in HeLa T‐rex Flp‐in cells. Stable mutants were selected based on their increased expression levels and longer half‐lives in the presence of cycloheximide (Fig EV2B and C). We consistently found that the deletion of several adjacent highly conserved C‐terminal regions in NIX and BNIP3 led to their increased expression compared to wild‐type versions. Specifically, the region of aa151–184 in NIX (adjacent regions aa151–170 and aa 171–184) and aa161‐225 in BNIP3 (adjacent regions aa161–192 and aa193–225) were expressed at higher levels than wild‐type counterparts (Fig EV2A–C).

Theoretically, the increased stability of the NIX and BNIP3 deletion mutants could be due to a combination of either the loss of ubiquitylation sites or the loss of FBXL4 binding site (and/or loss of post‐translational modifications that support recruitment of required factors). To determine whether the hyper‐stable mutants of NIX and BNIP3 exhibit reduced ubiquitylation, we performed the TR‐TUBE assay using hyper‐stable NIX (NIXΔ151‐170) and BNIP3 (BNIP3Δ181‐203). We found that both NIXΔ151‐170 and BNIP3Δ181‐203 were ubiquitylated to a lesser extent than wild‐type proteins (Fig EV2D). Notably, these deleted regions in NIX and BNIP3 contain several lysine residues, including a Lysine that has been reported to be modified with ubiquitin after MG132 treatment (NIX‐Lys154; Phosphosite database). We confirmed that the stable deletion mutants localised normally to mitochondria, demonstrating that their stabilisation is not due to mis‐localisation from the mitochondria (Fig EV2E).

### FBXL4 restricts NIX‐ and BNIP3‐dependent mitophagy via NIX/BNIP3 destabilisation

To test whether the elevated levels of NIX and BNIP3 in FBXL4‐deficient cells result in elevated mitophagy in basal conditions, we used the mt‐Keima mitophagy assay. FBXL4‐deficient U2OS cell lines exhibited increased mitophagy compared with parental cell lines and this was rescued by re‐introducing FBXL4 (Fig 3A–C). To test whether FBXL4 restricts BNIP3‐ and NIX‐dependent mitophagy, we depleted NIX and BNIP3 by siRNA in the FBXL4‐deficient cells in basal conditions and found that the elevated mitophagy in FBXL4‐deficient cells was reduced upon deletion of NIX and BNIP3 (Fig 3D–F).

**Figure EV3:**
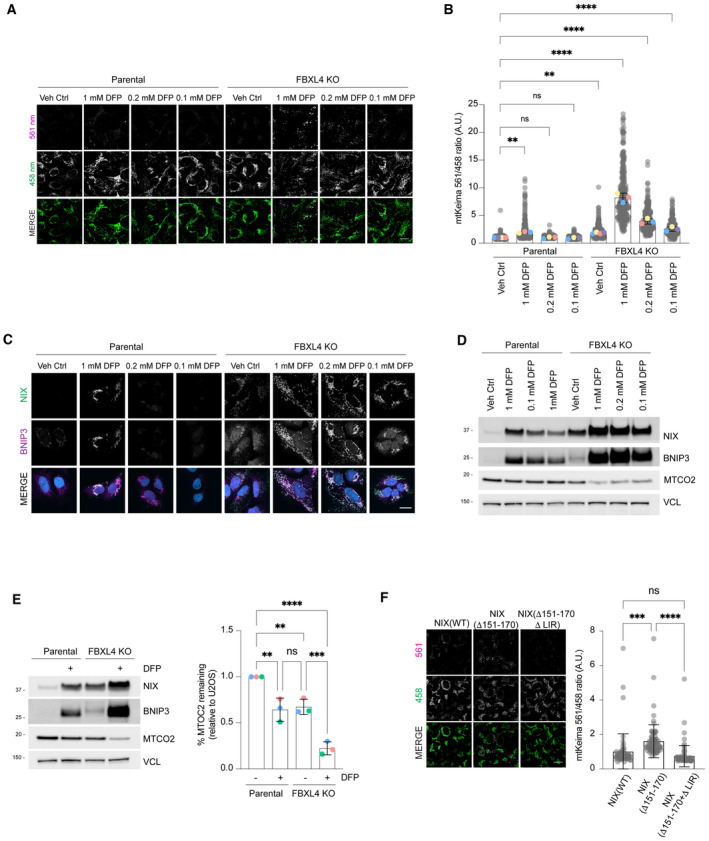
FBXL4 deficiency promotes mitophagy through NIX/BNIP3 stabilisation FBXL4‐deficient cells are ultra‐sensitive to DFP‐induced mitophagy. U2OS mt‐Keima cells and U2OS mt‐Keima FBXL4 KO cells were treated with DFP at specified concentrations for 24 h and analysed by live‐cell confocal microscopy. The emission signals obtained after excitation with the 458 nm laser (neutral pH) or 561 nm laser (acidic pH) are shown in green and magenta respectively.Quantification of mitophagy shown in panel (A). Mitophagy is represented as the ratio of mt‐Keima 561 nm fluorescence intensity divided by mt‐Keima 458 nm fluorescence intensity for individual cells normalised to the parental condition.Corresponding conditions from (A, B) were harvested for immunoblotting to analyse levels of NIX and BNIP.Analysis of NIX and BNIP localisation in parental cells or FBXL4 KO cells treated with specified concentration of DFP.Quantification of the MT‐CO2 protein levels in parental or FBXL4 KO cells treated with DFP. Western blotting was used to assess MT‐CO2 levels remaining relative to parental untreated cells.
*Mitophagy induced by hyper‐stable NIX* (*Δ151‐170*) *requires NIX's LC3 interaction domain*. Hela Flp‐in Keima cells stably expressing inducible NIX(WT), NIX(Δ151‐170) or NIX (151–170, LIRmut) were treated with doxycycline for 48 h. Mitophagy was quantified as in (B). Data information: In (B and F), translucent grey dots represent measurements from individual cells. In (B), coloured circles represent the mean ratio from independent experiments (*N* = 4 for Veh Ctrl conditions and *N* = 3 for other conditions) and the centre lines and bars represent the mean of the independent replicate means ± SD. In (E), the coloured circles represent the = densitometry values of MT‐CO2 normalised to VCL loading control from independent experiments. In (F), the centre lines and bars represent the mean of the individual cells ± SD (*N* = 1, > 60 cells analysed). For (B), *P* values were calculated from the mean values from independent experiments using one‐way ANOVA; for (F), *P* values were calculated from the ratios of the individual cells (*P < 0.05, **P < 0.005, ***P < 0.001, ****P < 0.0001). Scale bars = 20 μm. Source data are available online for this figure.

We next examined mitophagy in response to the combination of FBXL4 deficiency and DFP treatment. We observed that FBXL4‐deficient cells treated with DFP (at increasing concentrations) exhibited substantially enhanced mitophagy (Fig EV3A and B), as well as elevated NIX and BNIP3 levels (Fig EV3C and D) compared to parental cells treated with DFP, possibly suggesting that the elevated levels of mitophagy receptors sensitises cells to DFP‐induced mitophagy. In addition to mt‐Keima analysis, we also monitored the downregulation of mitochondrial protein MTCO2, finding that MTCO2 levels decreased upon DFP treatment and in FBLX4 knockout cells, and this reduction was increased in the combination treatment (Fig EV3E).

To directly determine the effect of NIX and BNIP3 stabilisation on mitophagy, we expressed the hyper‐stable mutants identified in Fig EV2 (NIXΔ150‐171, NIXΔ170‐184, BNIP3Δ181‐203 or BNIP3Δ193‐225) in mt‐Keima‐expressing HeLa cells and compared the induction of mitophagy to that induced by wild‐type NIX or BNIP3. In this inducible system, the wild‐type NIX and BNIP3 expression is minimal and therefore does not induce significant mitophagy. We observed that the induction of hyper‐stable NIXΔ150‐171 or NIXΔ170‐184 resulted in an approximately 2‐fold increase in the mean mitophagy ratio compared with wild‐type NIX (Fig 3G) in the absence of any external triggers for mitophagy. Likewise, the induction of hyper‐stable mutants of BNIP3 also triggered mitophagy when compared to wild‐type BNIP3 (Fig 3H). NIX‐dependent mitophagy has previously been shown to depend on NIX's LC3 interacting region (Novak *et al*, 2010; Wilhelm *et al*, 2022), and we also found that the deletion of the LIR domain in NIXΔ150‐171 abolished its ability to activate mitophagy (Fig EV3F). We conclude that that FBXL4 suppresses mitophagy by promoting the destabilisation of NIX and BNIP3 mitophagy receptors.

### MTDPS13 patient‐derived FBXL4 variants have impaired abilities to mediate NIX and BNIP3 turnover and restrict mitophagy

**Figure 4:**
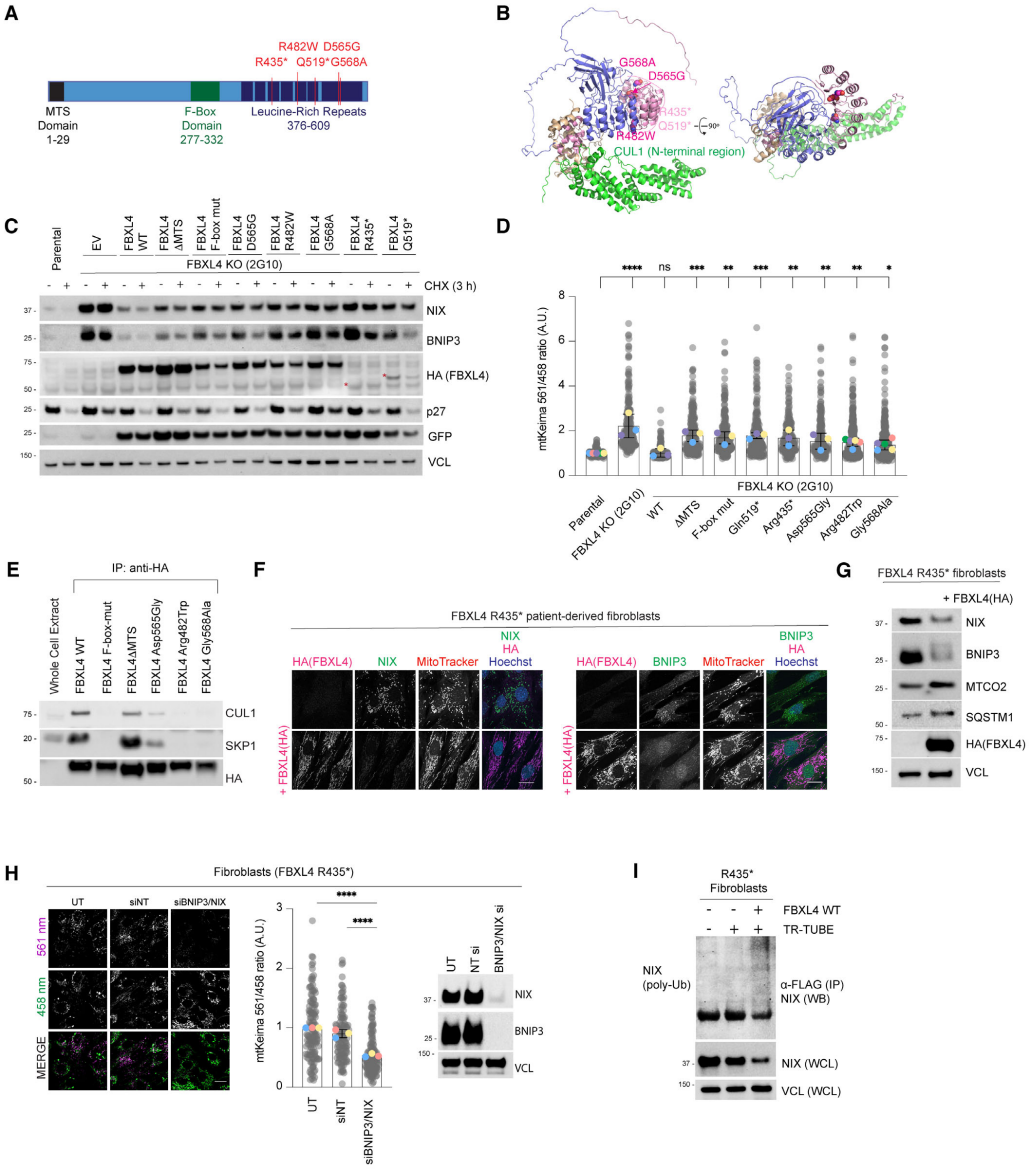
MTDPS13 patient‐derived FBXL4 variants do not efficiently assemble into an SCF complex and have impaired abilities to mediate NIX and BNIP3 turnover *Schematic representation of domain structure of FBXL4*. Pathological variants tested herein are shown in red.
*Alphafold2 structural modelling of FBXL4 and its complex formation with SCF components SKP1 and CUL1*. Pathogenic variants of FBXL4 indicated in magenta spheres. The pale pink section of the LRRs represents the region deleted by the truncation deletions (Arg435).
*FBXL4 patient‐derived variants exhibit reduced efficiency compared to wild‐type FBXL4 in mediating the downregulation and destabilisation of NIX and BNIP3.* U2OS FBXL4 KO (2G10) cells were rescued with constructs expressing wild‐type FBXL4‐HA, FBXL4(F‐box mut), FBXL4(ΔMTS) or specified patient variants. Cells were treated with cycloheximide for 3 h prior to harvesting. Samples were lysed, and immunoblotting was performed. GFP serves as a marker of transduction efficiency/transgene expression. EV = empty vector.
*FBXL4 patient‐derived variants exhibit reduced efficiency compared to wild‐type FBXL4 in suppressing mitophagy*. U2OS mt‐Keima cells (parental), U2OS mt‐Keima FBXL4 KO cells and U2OS mt‐Keima FBXL4 KO cells rescued with the specified FBXL4 constructs were visualised using live cell confocal microscopy. Mitophagy is represented as the ratio of mt‐Keima 561 nm fluorescence intensity divided by mt‐Keima 458 nm fluorescence intensity for individual cells normalised to untreated U2OS cells.
*FBXL4‐Arg482Trp and FBXL4‐Gly568Ala patient variants are less efficient than FBXL4 wild‐type at assembling into a complex with SKP1 and CUL1.* FBXL4‐KO cells expressing wild‐type FBXL4‐HA or FBXL4 variants were harvested and lysed. Whole‐cell extracts were subjected to immunoprecipitation (IP) with anti‐HA agarose beads and immunoblotting, as indicated.
*Expression of FBXL4‐HA into FBXL4‐deficient patient fibroblast cells causes down‐regulation of NIX and BNIP3 and extensive changes in mitochondrial morphology.* FBXL4‐deficient patient fibroblasts (derived from patients harbouring homozygous non‐sense mutation in FBXL4 at pArg435*) were transduced with FBXL4‐HA construct. Cells were stained with MitoTracker, fixed and co‐immunostained with antibodies to HA (to detect FBXL4) and either NIX or BNIP3.Steady‐state protein levels after expression of FBXL4‐HA‐C in FBXL4‐deficient patient fibroblasts analysed by Western blotting. FBXL4‐deficient patient fibroblasts were transduced with FBXL4‐HA‐C.
*NIX/BNIP3 depletion mediated by siRNA reduces mitophagy in FBXL4‐deficient patient fibroblast cells*. FBXL4‐deficient patient fibroblasts were transfected with siRNAs targeting both NIX and BNIP3 (NIX/BNIP3 si) or non‐targeting siRNA (NT si) (left). Live‐cell confocal microscopy was performed to visualise mitophagy, and quantification was performed as in D (middle). The siRNA‐mediated depletion of NIX and BNIP3 was evaluated by Western blotting (right).
*Ubiquitylation of endogenous NIX is restored upon expression of FBXL4 in patient‐derived cells*. Patient‐derived fibroblasts were transfected with FLAG‐tagged TR‐TUBE, as indicated. FLAG‐tagged TUBE protein was affinity purified from the corresponding lysates using FLAG‐beads, and the precipitates were analysed by immunoblotting using antibodies to endogenous NIX. Data information: In (D and H), translucent grey dots represent measurements from individual cells. Coloured circles represent the mean ratio from independent experiments. The centre lines and bars represent the mean of the independent replicates ± SD. In (D), *N* = 5 for Arg482Trp and Gly568Ala variant, *N* = 3 for other conditions; in H, *N* = 3. Over 100 cells were analysed per condition. *P* values were calculated based on the mean values using a one‐way ANOVA (*P < 0.05, **P < 0.005, ***P < 0.001, ****P < 0.0001). Scale bar = 20 μm. Source data are available online for this figure.

**Figure EV4:**
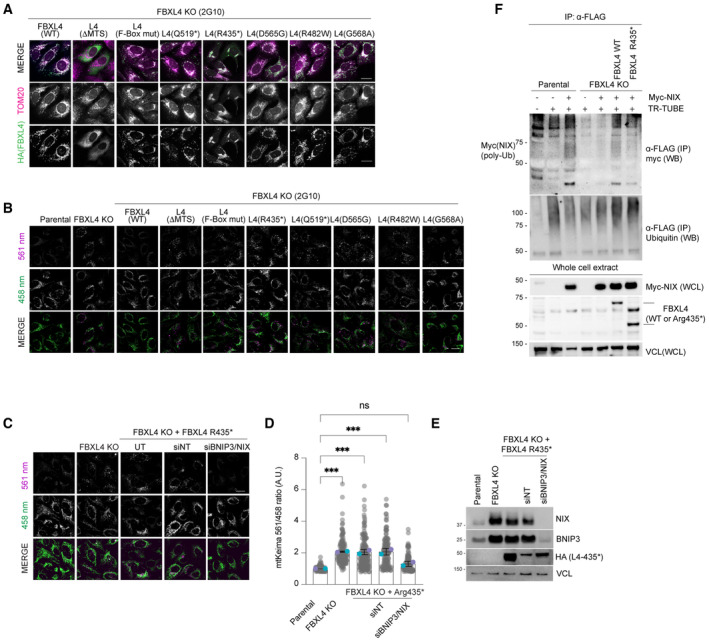
MTDPS13 patient‐derived FBXL4 variants do not efficiently assemble into an SCF complex and have impaired abilities to mediate NIX and BNIP3 turnover *Localisation of FBXL4 variants*. FBXL4 KO cells expressing FBXL4‐HA wild‐type or specified variants were fixed and stained for HA (to detect FBXL4 in green) or TOM20 (in magenta).
*FBXL4 patient‐derived variants exhibit reduced efficiency in mediating the suppression of mitophagy compared to wild‐type FBXL4*. U2OS mt‐Keima cells, U2OS mt‐Keima FBXL4 KO cells and U2OS mt‐Keima FBXL4 KO cells rescued with FBXL4 constructs were analysed by confocal microscopy. The emission signals obtained after excitation with the 458 nm laser (neutral pH) or 561 nm laser (acidic pH) are shown in green and magenta respectively. Quantification of these conditions is shown in Fig 4C.
Elevated mitophagy in FBXL4 KO cells expressing FBXL4Arg435* truncation variant is reduced when NIX/BNIP3 are depleted using siRNA. FBXL4 KO cells stably expressing FBXL4Arg435* were transfected with siRNAs targeting both NIX and BNIP3 (NIX/BNIP3 si) or non‐targeting siRNA (NT si). Live‐cell confocal microscopy was performed to visualise mitophagy.Quantification of mitophagy shown in panel (C). Translucent grey dots represent measurements from individual cells. Coloured circles represent the mean ratio from independent experiments (*N* = 2). The centre lines and bars represent the mean of the independent replicates ± SD. At least 50 cells were analysed per condition, and over 100 cells were analysed in total per condition. *P* values were calculated based on the mean values using a one‐way ANOVA (*P < 0.05, **P < 0.005, ***P < 0.001, ****P < 0.0001).Evaluation of the extent of NIX/BNIP3 depletion in (C, D) using Western blotting.
FBXL4Arg435* truncation variant is less efficient than wild‐type FBXL4 at promoting NIX ubiquitylation. Parental, FBXL4 KO cells, FBXL4 KO cells expressing either wild‐type FBXL4 or FBXL4‐Arg435* were transfected with TR‐TUBE and myc‐NIX, as indicated. Cell lysates obtained 48 h post‐transfection were immunoprecipitated with anti‐FLAG antibody, and the immunoprecipitates were analysed by immunoblotting using anti‐myc antibody (to detect ubiquitylated NIX or BNIP3). The line on the left marks a ladder of bands corresponding to polyubiquitylated myc‐BNIP3 or myc‐NIX. Data information: Scale bars = 20 μm. Source data are available online for this figure.

We tested whether the pathogenic *FBXL4* variants responsible for MTDPS13 (OMIM # 615471) interfere with FBXL4 function. FBXL4 possesses a typical F‐box domain that associates directly with SKP1, a C‐terminal LRR (leucine‐rich repeat) domain consisting of 12 repeats, and a unique N‐terminal β‐sheet domain with a nine‐stranded discoidin‐like fold (Fig 4A and B; Appendix Fig. S1). The N‐terminal domain of FBXL4 is not found in other FBXL family members and is predicted to form an intimate intramolecular interaction with the C‐terminal LRR domain (Fig 4B). Most of the pathogenic mis‐sense variations in *FBXL4* are in its C‐terminal LRR domain (the putative substrate‐binding region). To compare the efficacy of wild‐type FBXL4 and MTDPS13‐associated FBXL4 variants (Arg482Trp, Asp565Gly, Gly568Ala, Gln519* and Arg435*, based on RefSeqNM_001278716.2/NP_001265645.1) in promoting NIX and BNIP3 turnover, we conducted rescue experiments. Wild‐type FBXL4 effectively reduced the levels and half‐lives of NIX and BNIP3 to basal levels, while the disease‐associated FBXL4 variants were less effective (Fig 4C). The Gln519‐term and Arg435‐term truncation variants were expressed at significantly lower levels than wild‐type FBXL4; however, FBXL4 missense variants (Arg482Trp, Asp565Gly, Gly568Ala) were expressed at levels similar to wild‐type FBXL4, suggesting that the inability of these specific missense variants to degrade NIX and BNIP3 was not related to their expression levels. Despite their reduced function, the FBXL4 variants localised, like wild‐type FBXL4, to mitochondria (Fig EV4A).

The FBXL4‐deficient cells that were rescued with FBXL4 pathogenic variants displayed an increase in mitophagy compared to those rescued with wild‐type FBXL4, consistent with the elevated levels of NIX and BNIP3 observed (Figs 4D and EV4B). To further establish the correlation between mitophagy and elevated NIX/BNIP3 levels in cells expressing patient‐derived FBXL4 variants, we demonstrated that siRNA‐mediated depletion of NIX and BNIP3 led to a decrease in the elevated mitophagy in cells expressing FBXL4‐Arg435* (Fig EV4C–E). Furthermore, using the TR‐TUBE assay, we demonstrated that the FBXL4‐Arg435* variant is less efficient than wild‐type FBXL4 in mediating NIX ubiquitylation (Fig EV4F).

To explore how the MTSDP13‐associated FBXL4 variants affect NIX and BNIP3 degradation, we next examined the ability of FBXL4 variants to bind to SKP1 and CUL1, core members of the SCF complex. Although the disease‐associated variants that we assessed are in the LRR region outside the F‐box domain, we found that Arg482Trp, Gly568Ala, and to a lesser extent Asp565Gly variants were less proficient than wild‐type FBXL4 at binding to SKP1 and CUL1 (Fig 4E). Mutations that affect substrate binding could impair SCF‐FBXL4 complex assembly, as is the case for FBXL3 which only assembles an SCF complex in the presence of substrate (Yumimoto *et al*, 2013). Alternatively, the mutations could broadly affect protein folding and in that way impede SCF assembly.

Fibroblasts derived from a patient homozygous for the p.Arg435* *FBXL4* variant have previously been reported to have elevated mitophagy, and therefore, we examined NIX and BNIP3 levels in these cells (Bonnen *et al*, 2013; Alsina *et al*, 2020). We observed that NIX and BNIP3 levels were easily detectable in this cell line under basal conditions but were downregulated when wild‐type FBXL4‐HA was expressed (Fig 4F and G). In many of these cells, the expression of FBXL4 also caused extensive changes in mitochondrial network morphology, changing from fragmented in the patient‐derived cells to tubular in the cells that were complemented with FBXL4 (Fig 4F). FBXL4 expression in the patient‐derived cells also caused an increase in the levels of MT‐CO2 (suggestive of decreased mitochondrial degradation; Fig 4G). Suggesting that NIX and/or BNIP3 mediate the high mitophagy in the FBXL4 patient cells, siRNA‐mediated depletion of NIX and BNIP3 significantly reduced the high mitophagy observed in this patient cell line (Fig 4H). Lastly, using the TR‐TUBE assay, we demonstrated that we could only detect ubiquitylation of NIX in patient‐derived cells complemented with FBXL4‐HA (Fig 4I). Taken together, our results suggest that MTDPS13‐associated pathogenic FBXL4 variants have impaired abilities to mediate the degradation of NIX and BNIP3 mitophagy receptors, resulting in their accumulation and consequent increased mitophagy.

## Discussion

The cellular triggers promoting basal mitophagy are poorly understood. Our study demonstrates that FBXL4 plays a crucial role in suppressing mitophagy under basal conditions by restricting the abundance of NIX and BNIP3 mitophagy receptors. Thus, NIX and BNIP3 are negatively regulated by two different CRLs: (i) the CRL2‐VHL complex which mediates the turnover of HIF1α and thereby inhibits NIX and BNIP3 transcription and (ii) the SCF‐FBXL4 complex at the mitochondrial outer membrane. Our data reveal that mitophagy is actively suppressed by the continuous degradation of NIX and BNIP3, which was previously known to be upregulated at the level of transcription. The multiple mechanisms converging to regulate the abundance of NIX and BNIP3 have presumably evolved to ensure tight regulation of mitophagy levels which enables cells to respond precisely and rapidly to changes in metabolic signals.

Dysregulation of FBXL4 function results in encephalopathic mtDNA depletion syndrome 13 (MTDPS13). Despite the serious consequences of *FBXL4* mutations (Bonnen *et al*, 2013; Gai *et al*, 2013; Alsina *et al*, 2020), the molecular functions of the FBXL4 protein have remained elusive as no mitochondrial substrates for FBXL4 have been identified to date. Our data uncover a mechanistic link between FBXL4 and mitophagy in MTDPS13, demonstrating that MTDPS13‐derived FBXL4 variants are defective in mediating the turnover NIX and BNIP3 mitophagy receptors.

How FBXL4 activity is regulated remains to be elucidated. It is an interesting prospect that FBXL4 localisation or activity could be inhibited on specific mitochondria selected for mitophagy to allow NIX and BNIP3 accumulation. Unlike the transcriptional regulation of NIX and BNIP3, such local regulation of FBXL4 (and thus NIX and BNIP3) would represent a mechanism to control turnover of selected mitochondria, rather than pools of mitochondria that are removed by mitophagy for metabolic re‐programming in response to hypoxia (Zhang *et al*, 2008). Similarly, post‐translational modifications on NIX and BNIP3 that stabilise them through disrupting their recognition by SCF^FBXL4^ may occur on specific mitochondria, allowing selective targeting.

It should be noted that the accumulation of NIX and BNIP3 on the outer membrane of all mitochondria occurs non‐selectively upon loss of FBXL4. Despite the stabilisation of NIX and BNIP3, only a proportion of tagged mitochondria undergo mitophagy despite the stabilisation of NIX and BNIP3, implying that additional signalling or stochastic events contribute to mitophagy induction. How mitophagy receptor stabilisation cooperates with other signalling mechanisms and the fission–fusion machinery to facilitate mitophagy is unclear. Multiple mechanisms have been reported to facilitate mitophagy induction via mitophagy receptors, including phosphorylation (Liu *et al*, 2012; Chen *et al*, 2014; Wu *et al*, 2014; Rogov *et al*, 2017) and dimerisation (Marinkovic *et al*, 2021).

The binding interface through which FBXL4 engages NIX and BNIP3 remains unknown, although we were able to identify regions within NIX and BNIP3 that when deleted, resulted in stabilisation of these proteins and consequently increased mitophagy in the absence of overt mitochondrial stress. It is possible that the C‐terminal regions represent sites for post‐translational modifications that facilitate recognition by the ligase or recruitment of other required factors. Future investigations will focus on understanding the precise mechanisms by which FBXL4 recognises NIX and BNIP3.

## Materials and Methods

### Antibodies

Mouse monoclonal anti‐TOM20 (clone 29; 612278) and mouse monoclonal anti‐p27 (clone 57/Kip1/p27; 610242) were obtained from BD Biosciences. Mouse monoclonal anti‐TIM50 (clone C‐9; sc‐393678), mouse monoclonal anti‐BNIP3 (clone ANa40; sc‐56167: IF and WB), mouse monoclonal anti‐NIX (clone H‐8; sc‐166332: IF and WB), mouse monoclonal anti‐vinculin (VCL, clone G‐11; sc‐55465), mouse monoclonal anti‐γ‐Tubulin (clone C‐11; sc‐17787), rabbit polyclonal anti‐HDAC6 (sc‐11420) and mouse monoclonal anti‐GFP (B‐2, sc‐9996) were obtained from Santa Cruz Biotechnology. Mouse monoclonal anti‐HA (clone 16B12; 901513) was obtained from BioLegend. Rabbit monoclonal anti‐BNIP3 (clone EPR4034; ab109362: WB) was obtained from Abcam. Mouse monoclonal anti‐Myc (clone 9B11; 2276S), mouse monoclonal anti‐HA Alexa Fluor™ 488 conjugate (clone 6E2; 2350S), rabbit monoclonal anti‐NIX (clone D4R4B;12396: IF and WB), rabbit monoclonal anti‐HA (clone C29F4; 3724S), rabbit monoclonal anti‐LC3B (clone D11; 3868S), rabbit SQSTM1 (D5E2; 8025) and rabbit monoclonal anti‐HIF1α (clone D1S7W; 36169S) were obtained from Cell Signaling Technology. Rabbit polyclonal anti‐CUL1 (718700) and anti‐Ubiquitin (PA1‐187) were obtained from Thermo Fisher Scientific. Mouse monoclonal anti‐FLAG (clone M2; F3165) and rabbit polyclonal anti‐FLAG (SAB4301135) were obtained from Sigma‐Aldrich. Rabbit anti‐SKP1 was generated in the Pagano laboratory (Pagan *et al*, 2015). Secondary donkey anti‐mouse IgG Alexa Fluor™ 488 (A21202), donkey anti‐mouse IgG Alexa Fluor™ 555 (A31570), donkey anti‐mouse IgG Alexa Fluor™ 594 (A21203), donkey anti‐mouse IgG Alexa Fluor™ 647 (A31571), donkey anti‐rabbit IgG Alexa Fluor™ 488 (A21026), donkey anti‐rabbit IgG Alexa Fluor™ 555 (A31572) and donkey anti‐rabbit IgG Alexa Fluor™ 647 (A31573) were obtained from Thermo Fisher Scientific. Goat anti‐rabbit IgG Atto 647N (40839) was purchased from Sigma‐Aldrich.

### DNA constructs

pCHAC‐mt‐mKeima was a gift from R. Youle (RRID: Addgene 72342) (Lazarou *et al*, 2015). pLIX_402 was a gift from David Root (Addgene plasmid # 41394). MAC (BirA‐Ha‐Strep‐tag II)‐N was a gift from Markku Varjosalo (Addgene plasmid # 108078). FLAG‐tagged TR‐TUBE has been previously published (Yoshida *et al*, 2015) and was provided by the RIKEN BRC through the National Bio‐Resources Project of the MEXT/AMED, Japan. pcDNA5/FRT/TO/FLAG‐S‐tag has been previously published (Pagan *et al*, 2015). Dominant‐negative Cullin constructs, including pcDNA3‐Flag‐HA‐DN‐CULLIN1 (1–252), pcDNA3‐Flag‐HA‐DN‐CULLIN3 (1–240), pcDNA3‐Flag‐HA‐DN‐CULLIN4 (1–237) and pcDNA3‐Flag‐HA‐DN‐CULLIN5 (1–228), were generated by site‐directed mutagenesis. The pcDNA3.1(+)‐N‐Myc‐BNIP3 (EAW49143.1, note that this sequence encodes a 259 amino acid BNIP3 protein, and has been subsequently replaced with a sequence encoding a 194 amino acid BNIP3 protein), pcDNA3.1(+)‐N‐Myc‐NIX (NM_004331.3), pDONR‐N‐FLAG‐BNIP3, pDONR‐N‐FLAG‐BNIP3Δ141‐160, pDONR‐N‐FLAG‐BNIP3Δ161‐192, pDONR‐N‐FLAG‐BNIP3Δ193‐225, pDONR‐N‐FLAG‐BNIP3Δ181‐203, pDONR‐N‐FLAG‐NIX, pDONR‐N‐FLAG‐NIXΔ120‐150, pDONR‐N‐FLAG‐NIXΔ151‐170, pDONR‐N‐FLAG‐NIXΔ151‐170+ΔLIR(35WVEL38‐35AAAA‐38), pDONR‐N‐FLAG‐NIXΔ171‐184, pcDNA3.1(+)‐C‐HA‐FBXL4(encoding NP_036292.2), pcDNA3.1‐C‐eGFP‐FBXL4 and pDONR‐C‐HA‐FBXL4 were generated by Genscript^®^. pLV‐FBXL4‐C‐HA:IRES:EGFP, pLV‐FBXL4‐C‐HA‐F‐BOX mut(LP283AA;LP297AA):IRES:EGFP, pLV‐FBXL4‐C‐HA‐ ΔMTS(Δ1‐29):IRES:EGFP, pLV‐FBXL4‐C‐HA(Asp565Gly):IRES:EGFP, pLV‐FBXL4‐C‐HA(Arg482Trp):IRES:EGFP, pLV‐FBXL4‐C‐HA(Gly568Ala):IRES:EGFP, pLV‐FBXL4‐C‐HA(Gly519 term):IRES:EGFP and pLV‐FBXL4‐C‐HA(Arg435 term):IRES:EGFP were generated by VectorBuilder. The Gateway cloning system (Thermo Fisher Scientific) was used to generate pcDNA5/FRT/TO/FLAG and pLIX‐402 based constructs.

### CRISPR/Cas9‐mediated genome editing

The pSpCas9 BB‐2A‐Puro (PX459) plasmid backbone was used to create the following guide RNA (gRNA) plasmids (created by Genscript^®^): BNIP3 CRISPR gRNA plasmid (gRNA targeting sequence: TCTTGTGGTGTCTGCGAGCG), NIX CRISPR gRNA plasmid (gRNA targeting sequence: TAGCTCTCAGGTGTGTCGGG) and FBXL4 CRISPR gRNA plasmids (gRNA targeting sequence: CAATTCAAGGCGTACTAATT; gRNA targeting sequence 2: CCCCACAAATCTTATACGAC).

To generate CRISPR/Cas9 knockout (KO) cell lines, cells were transiently transfected with the CRISPR gRNA plasmids targeting the gene(s) of interest. Twenty‐four hours post‐transfection, cells were selected with puromycin (Sigma) for 72 h. They were then diluted as one cell per well into 96‐well plates until single colonies formed. Successful editing was screened for by immunoblot analysis and/or indirect immunofluorescence microscopy. Sanger sequencing was used to confirm the presence of frameshift indels in the potential KO clones first identified by immunoblotting or immunofluorescence screening. For this, genomic DNA was isolated using the salting out method (Miller *et al*, 1988). In brief, cells were lysed in lysis buffer (50 mM Tris–HCL, SDS 1%) and genomic DNA was precipitated following the adding of 5 M NaCl, proteinase K and absolute ethanol. Then, PCR was performed to amplify the targeted regions. The PCR product was subcloned into pCR™‐BluntII‐TOPO^®^ vector (Zero Blunt^®^ TOPO^®^ PCR cloning Kit, Invitrogen™) and sequenced with M13 forward primer to character the indels (which are described in Table EV1).

To validate BNIP3 knockout clones, a set of primers including BNIP3 forward (FWD) (5′‐GAGGAAGAGTTTGGCTCTGGCAGG‐3′) and BNIP3 reverse (RVS) (5′‐CGGTGTATCCCTGATGGCAG‐3′) was used. To validate NIX KO clones, a set of primers including NIX FWD (5′‐AGTGCAGAACATTTTGGGAGT‐3′) and NIX RVS (5′‐AAATCACCCGTCTTCTGCGT‐3′) was used. To validate FBXL4 KO clones, two sets of primers including FBXL4 FWD (Guide1—5′‐TTTTAGCCTAACCATTCATATTTCA‐3′ or Guide2—5′‐CCTTAAGGGACCAGTAGATCTCA‐3′) and FBXL4 RVS (Guide1 5′‐CTGCCAGCATTTTGGCTTAC‐3′ or Guide2 5′‐CAATGCTCAATTACCGATGC‐3′) were used.

### Cell culture and chemicals

Cell lines were grown at 37°C in a humidified incubator containing 5% CO_2_. HeLa cells (ATCC CCL‐2), U2OS (ATCC HTB‐96) and HEK293T (ATCC CRL‐3216) cells were maintained in Dulbecco's modified Eagle's medium/nutrient mixture F‐12 GlutaMAX™ (DMEM/F‐12; Thermo Fisher Scientific) supplemented with 10% foetal bovine serum. Fibroblast cells derived from a patient with homozygous p.Arg435* *FBXL4* have been previously published (Bonnen *et al*, 2013) (Alsina *et al*, 2020) and were cultured in DMEM/F‐12 GlutaMAX™ with 20% FBS and 5 mg/ml penicillin and streptomycin (Thermo Fisher Scientific). All cell lines were regularly screened for mycoplasma contamination. Where indicated, cells were treated with cycloheximide (CHX; 100 μg/ml; 66‐81‐9), deferiprone (DFP; 1 mM; 379409), DMOG (0.5 mM; D3695) and echinomycin (10 nM; SML0477), which were purchased from Sigma. MLN4924 (0.5 μM; 85923S) was obtained from Cell Signaling Technology. MG132 (10 μM; 474787) was purchased from Merck.

### Cell line generation

FLAG‐S tag version of BNIP3(WT), BNIP3(Δ141‐160), BNIP3(Δ161‐192), BNIP3(Δ193‐225), BNIP3(Δ181‐203), NIX(WT), NIX(Δ120‐150), NIX(Δ151‐170) and NIX(Δ171‐184) was generated using pcDNA5/FRT/TO (Thermo Fisher). MAC‐N, MAC‐BNIP3 and MAC‐NIX were generated using the MAC‐tag‐N, which was a gift from Markku Varjosalo (Addgene plasmid # 108078; http://n2t.net/addgene:108078; RRID:Addgene_108078). Constructs were co‐transfected with pOG44 into HeLa‐T‐rex Flp‐in cells to generate inducible cell lines using Flippase (Flp) recombination target (FRT)/Flp‐mediated recombination technology in HeLa‐T‐rex Flp‐in cells, as previously described (Pagan *et al*, 2015). Twenty‐four hours post‐transfection, cells were selected with Hygromycin B (400 μg/ml) for approximately 10 days. HeLa‐T‐rex Flp‐in cell lines were subsequently maintained in Hygromycin B (200 μg/ml). To induce expression, cells were treated with 0.5 μg/ml doxycycline (Sigma; 10592‐13‐9).

To generate stably transfected cell lines, retrovirus (pCHAC‐mt‐mKeima) and lentiviruses (pLV constructs) were packaged in HEK293T cells. HeLa or U2OS cells were transduced with virus for 48 h with 10 μg/ml polybrene (Sigma), then optimised for protein expression via fluorescence sorting or puromycin selection.

### mRNA analysis

RNA was extracted using the RNeasy Kit (Qiagen) and Superscript IV first strand cDNA synthesis kit (Thermo Fisher Scientific). Quantitative PCR analysis with SYBR Green PCR Master Mix (Applied Biosystems) was performed according to standard procedures. Primer sequences were: ACTB FWD 5′‐CTCACCGAGCGCGGCTACAG‐3′, ACTB RVS 5′‐CAGGCAGCTCGTAGCTCTTCTC‐3′, FBXL4 FWD 5′‐TTTAGCAGTGCTGTCCTCGG‐3′, and FBXL4 RVS 5′‐TGAGCAGTGCTGTTTGCTCTA‐3′.

### Transfection

Plasmid transfections were performed using Lipofectamine 2000 (Thermo Fisher Scientific) and siRNA transfections were performed using Lipofectamine RNAiMAX (Thermo Fisher Scientific), as per manufacturer's instructions. ON‐TARGETplus Non‐targeting Control Pool (Dharmacon; D‐001810‐01) was used as the siRNA control. ON‐TARGETplus siCUL1 pool (L‐004086‐00), siCUL2 pool (L‐007277‐00), siCUL3 pool (L‐010224‐00), siCUL4 pool (L‐012610‐00), siCUL5 pool (L‐019553‐00), siFBXL4 pool (L‐013564‐00), siHIF1α pool (L‐004018‐00), siFBXL5 pool (L‐012424‐00), siFBXO38 pool (L‐018163‐00), siFBXW12 pool (L‐032001‐00), siBNIP3 pool (M‐004636‐01‐0005) and siNIX pool (M‐11815‐01‐0005) were purchased from Dharmacon™ (Horizon Discovery).

### Immunoblotting

Immunoblotting was performed as previously described (Pagan *et al*, 2015). In brief, cells were harvested and subsequently lysed in SDS lysis buffer (50 mM Tris and 2% SDS) at 97°C for 15 min. Protein extracts were quantified using Direct Detect^®^ Assay‐free Cards (Merck; DDAC00010) or Pierce Bicinchoninic Acid (BCA) assay (Thermo Fisher Scientific; 23250) and prepared for gel electrophoresis in Bolt™ LDS Sample Buffer (Invitrogen™; B0008). Equal amounts of protein samples were resolved on SDS‐PAGE (BOLT pre‐cast 4–12% gradient gels, Invitrogen™) and transferred onto methanol‐activated Immobilon^®^‐P PVDF Membrane (0.45 μm pore size) (Merck; IPVH00010) using BOLT gel transfer cassettes and BOLT transfer buffer (Invitrogen™; BT0006), according to the manufacturer's instructions. The membranes were blocked in 5% skim milk for 1 h at room temperature and then incubated with indicated primary antibodies at 4°C overnight and secondary peroxidase‐conjugated goat anti‐rabbit or goat anti‐mouse antibodies for 1 h at room temperature. The chemiluminescence signal was acquired using Pierce ECL Western blotting substrate (Thermo Fisher Scientific; 32106) or Pierce SuperSignal West Femto Substrate (Thermo Fisher Scientific; 34094) and ChemiDoc™ Imaging System (Bio‐Rad).

### Co‐immunoprecipitation assays

Cells were lysed in a Tris‐Triton lysis buffer (50 mM Tris‐Cl pH 7.5, 150 mM NaCl, 10% glycerol, 1 mM EDTA, 1 mM EGTA, 5 mM MgCl_2,_ 1 mM β‐glycerophosphate and 1% Triton) containing protease inhibitor cocktail (Rowe Scientific; CP2778) and PhosSTOP EASYpack Phosphatase Inhibitor Cocktail (Roche; 4906837001) on ice for 30 min. Cell lysates were collected by centrifugation at 21,130 *g* for 10 min at 4°C. To immunoprecipitate exogenously expressed FLAG‐tagged or HA‐tagged proteins, cell lysates were incubated in a rotating incubator for 1 h at 4°C with bead‐conjugated FLAG (Sigma; A2220) and bead‐conjugated HA (Thermo Fisher Scientific; 88837) respectively. The immunoprecipitates were washed with Tris‐Triton lysis buffer five times prior to elution with Bolt™ LDS Sample Buffer and Western blotting.

### BioID pulldown

Stable cells expressing doxycycline‐inducible MAC(BirA‐HA‐Strep‐tagII)‐BNIP3, MAC‐NIX or MAC‐N were generated and subsequently transduced with pLV‐FBXL4‐C‐HA. Cells grown in 10 cm dishes were treated with 50 μM Biotin for 24 h. Cell pellets were lysed in RIPA lysis buffer (50 mM Tris–HCl pH 7.5, 150 mM NaCl, 1% NP‐40, 1 mM EDTA, 1 mM EGTA, 0.1% SDS, protease inhibitors and 0.5% sodium deoxycholate) at 4°C for 1 h on a rotator. Lysates were sonicated (2 × 10 s bursts with 2 s rest in between) on ice at 50% amplitude.

Lysates were then centrifuged for 30 min at 21,300 *g* at 4°C. Biotinylated proteins were captured using Pierce Streptavidin Magnetic Beads (Thermo Fisher Scientific, 88817) at 4°C on a rotator for 3 h. Magnetic beads collected on magnet for 1 minute between wash steps. The magnetic beads were washed with RIPA buffer (minus deoxycholate) 4 times prior to elution with 25 mM biotin at 95°C.

### TUBE assay to detect polyubiquitylated proteins

Cells grown in 10 cm dishes were transiently transfected with 5 μg of FLAG‐tagged TR‐TUBE (Yoshida *et al*, 2015) and 5 μg of myc‐tagged BNIP3 or myc‐tagged NIX. For immunoaffinity purification of ubiquitylated proteins, cells were lysed in Tris‐Triton lysis buffer (50 mM Tris‐Cl pH 7.5, 150 mM NaCl, 10% glycerol, 1 mM EDTA, 1 mM EGTA, 5 mM MgCl_2,_ 1 mM β‐glycerophosphate, 100 mM iodoacetamide and 1% triton) and harvested 48 h post‐transfection. Whole‐cell lysates were incubated for 1 h with ANTI‐FLAG^®^ M2 Affinity Gel (Sigma; A2220) followed by extensive washing. Bead‐bound proteins were eluted using Bolt™ LDS Sample Buffer.

### Indirect immunofluorescence

Adherent cells on coverslips were fixed in ice‐cold methanol for 10 min at −20°C (for most antibodies) or fixed in 4% PFA for 1 h (for BNIP3‐ANa40 antibody). Fixed cell monolayers were blocked with 2% BSA in PBS for 30 min to reduce non‐specific binding. Cells were then sequentially labelled with diluted primary antibodies and corresponding secondary antibodies for 1 h at room temperature. Coverslips were mounted on glass microscope slides using Fluorescent Mounting Medium (Dako; S3023) or Prolong Diamond Antifade Mountant (Thermo Fisher Scientific; P36965). Images in Figs 2E and EV2E were acquired at room temperature using a DeltaVision Elite inverted microscope system (GE Healthcare) using a ×100/1.4NA Oil PSF Objective from Olympus. Optical sections were processed using the SoftWorx deconvolution algorithm. Images in Fig EV1B and E were acquired using a Leica DMi8 SP8 Inverted confocal microscope equipped with 63× Plan Apochromatic objective. Images in Figs 1D, 2B, 3C, 4F and EV4A were acquired using a Zeiss LSM900 Fast AiryScan2 Confocal microscope with a 63× C‐Plan Apo NA 1.4 oil‐immersion objective. Image deconvolution was performed using ZEN Blue 3D software (version 3.4).

### Protein structural prediction, modelling and visualisation

The structural predictions of human FBXL4 (Q9UKA2) were performed using the AlphaFold2 neural‐network (Jumper *et al*, 2021) implemented within the freely accessible ColabFold pipeline (Mirdita *et al*, 2022). The following identifiers were used: Human FBXL1‐SKP1‐CUL1‐Rbx1‐ARIH1‐Ub‐CKS1B cryoEM structure (PDB ID 7B5M), Human SKP1 sequence (P63208) and Human CUL1 sequence (Q13616). For each modelling experiment, ColabFold was executed using default settings where multiple sequence alignments were generated with MMseqs2 (Mirdita *et al*, 2019) to produce five separate models per structure that were then subjected to energy minimisation with Amber (Eastman *et al*, 2017). In this instance, we verified that AlphaFold2 would produce a reliable predicted complex of the FBXL4 adaptor bound to SKP1 and the N‐terminal region of CUL1. For producing images, structures were rendered with Pymol (Schrodinger, USA; https://pymol.org/2/).

### mt‐Keima assay

The mt‐Keima assay was performed as previously described (Sun *et al*, 2017). Dual‐excitation (561/458 nm) images were acquired using a Leica DMi8 SP8 Inverted confocal microscope equipped with a 63× Plan Apochromatic objective and environmental chamber (set to 5% CO_2_ and 37°C). Quantitative analysis of mitophagy with mt‐Keima was performed with Image J/Fiji software. Single cells were segregated from fields of view by generating regions of interest (ROI). The selected ROI was cropped and split into separate channels, prior to threshold processing. The fluorescence intensity of mt‐Keima 561 nm (lysosomal signal) and mt‐Keima 458 nm (mitochondrial signal) at the single‐cell level was measured and the ratio 561 nm/458 nm was calculated.

### Statistical analysis

Statistical comparisons were conducted using GraphPad Prism 9.0 software. The centre line and error bars on graphs represent the averaged biologically independent replicates ± SD. Data from three or more biologically independent experimental replicates were used for all statistical comparisons (except where specified in the figure legends). Experiments were conducted without blinding or randomisation. No statistical tests were used to pre‐determined sample sizes. Similar data variances were observed between groups. For data representing a single time‐point and condition, one‐way ANOVA was used for statistical comparisons between means, followed by *post hoc* testing using Fisher's LSD test. *P* values greater than 0.05 were considered non‐significant.

## Author contributions

**Julia Pagan:** Conceptualization; resources; supervision; funding acquisition; validation; writing – original draft; project administration; writing – review and editing. **Giang Nguyen‐Dien:** Investigation; writing – review and editing. **Keri‐Lyn Kozul:** Investigation; writing – review and editing. **Yi Cui:** Investigation. **Brendan Townsend:** Investigation. **Soo Siang Ooi:** Investigation. **Michele Pagano:** Writing – review and editing. **Michael Lazarou:** Investigation; writing – review and editing. **Robert Taylor:** Resources. **Brett M Collins:** Visualization; writing – review and editing. **Robert G Parton:** Investigation; writing – review and editing. **Prajakta Kulkarni:** Investigation. **Nissa Carrodus:** Investigation. **Steven Zuryn:** Writing – review and editing. **Sean Millard:** Supervision; writing – review and editing. **Antonio Marzio:** Investigation. **Mathew Jones:** Supervision; investigation; writing – review and editing.

## Disclosure and competing interests statement

M.P. is a scientific cofounder of SEED Therapeutics; receives research funding from and is a shareholder in Kymera Therapeutics; and is a consultant for, a member of the scientific advisory board of, and has financial interests in CullGen, SEED Therapeutics, Triana Biomedicines, and Umbra Therapeutics. However, no research funds were received from these entities, and the findings presented in this manuscript were not discussed with any person in these companies. The other authors have no competing interests to declare.

## Supporting information



Expanded View Figures PDF

Table EV1

Source Data for Expanded View

PDF+

Source Data for Figure 1

Source Data for Figure 2

Source Data for Figure 3

Source Data for Figure 4

Appendix

## Data Availability

This study includes no data deposited in external repositories.
