# Supplementary material for: FBXL4 suppresses mitophagy by restricting the accumulation of NIX and BNIP3 mitophagy receptors
Source: EMBO J. 2023 May 10;42(13):e112767. doi: 10.15252/embj.2022112767 (PMC10308361; doi:10.15252/embj.2022112767)
Supplement: Supplementary file 1 — Expanded View Figures PDF [file EMBJ-42-e112767-s004.pdf]

Expanded View Figures

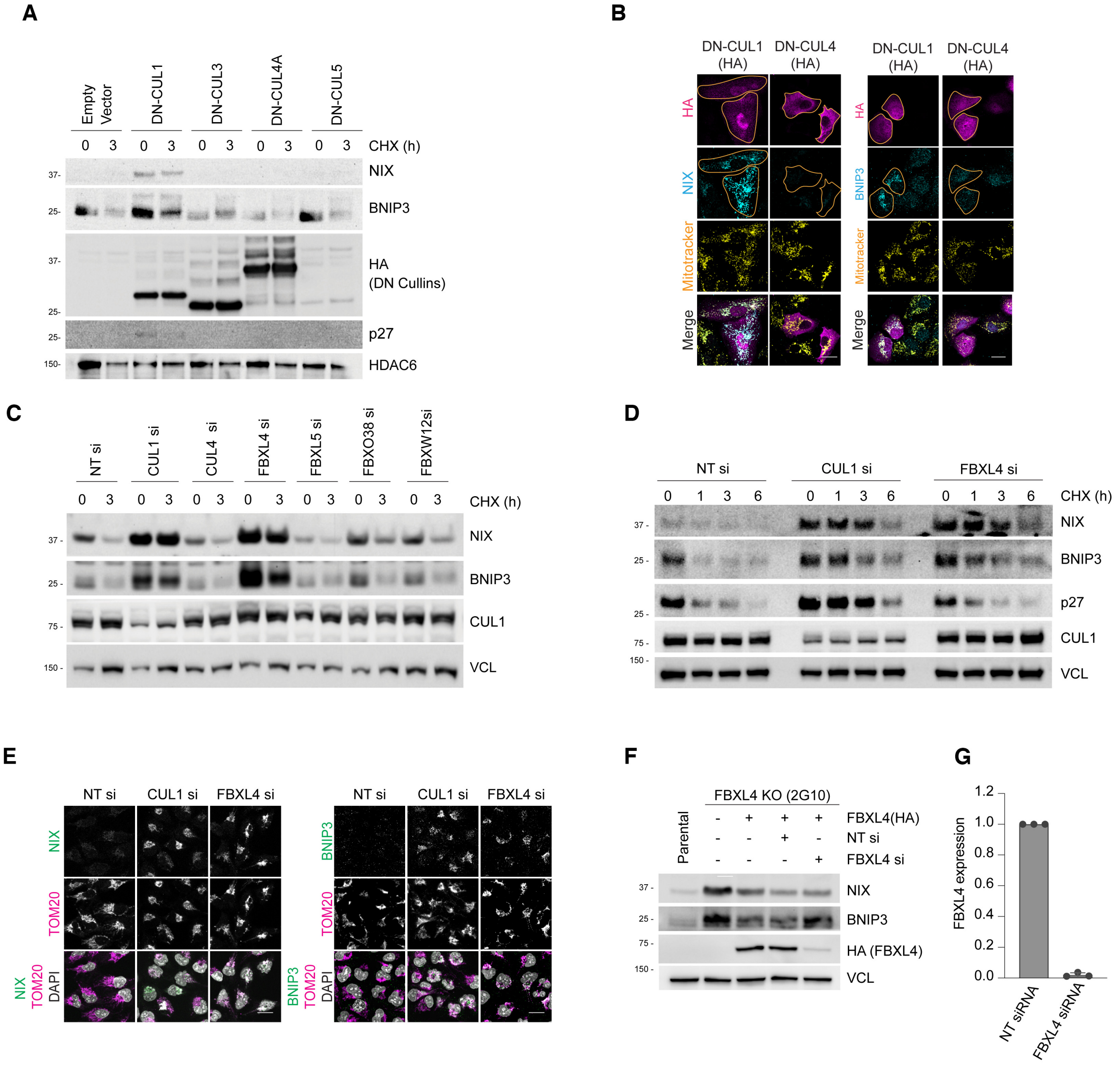

Figure EV1.

© 2023 The Authors

Figure EV1. Identification of SCF-FBXL4 as a negative regulator of NIX and BNIP3 stability.

A *Expression of dominant-negative* (*DN*) *Cullin 1 results in an increase in the levels and half-life of NIX and BNIP3*. HeLa-T-REx-Flp-in cells were transfected with FLAG-HA-tagged dominant-negative CUL1, CUL3, CUL4A and CUL5 or an empty vector. Cells were treated with cycloheximide for 3 h followed by immunoblotting with the indicated antibodies.

B *Expression of dominant-negative* (*DN*) *Cullin 1 results in the accumulation of NIX and BNIP3 at mitochondria*. U2OS cells were transfected with FLAG-HA-tagged DN-CUL1 or FLAG-HA-tagged DN-CUL4 and immunostained for both HA and either NIX or BNIP3. An orange line marks the edge of the individual cells expressing the dominant-negative cullin protein.

C *Screen for F-box proteins required for turnover of NIX and BNIP3*. U2OS cells were transfected with the indicated siRNAs. Total-cell lysates were subject to immunoblotting as shown. NIX and BNIP3 are stabilised by depletion of CUL1 and FBXL4 (but not other F-box proteins). NT = non-targeting.

D *NIX and BNIP3 are upregulated and stabilised by depletion of FBXL4 and CUL1*. U2OS cells were transfected with non-targeting siRNA, CUL1 siRNA or FBXL4 siRNA. Cells were treated with cycloheximide for the indicated times prior to immunoblotting with the specified antibodies.

E *Depletion of FBXL4 and CUL1 results in NIX and BNIP3 accumulation at mitochondria*. U2OS cells were transfected with non-targeting siRNA, CUL1 siRNA or FBXL4 siRNA. Cells were fixed and stained with the indicated antibodies.

F *Evaluation of the efficacy of FBXL4 siRNA to reduce exogenous HA-tagged FBXL4 protein levels*. U2OS FBXL4 knockout cells (which are described in Fig 2 and Table EV1) constitutively expressing FBXL4-HA (FBXL4 tagged with C-terminal HA) were transfected with non-targeting siRNA or FBXL4 siRNA. Immunoblotting was performed as indicated.

G U2OS cells were transfected with non-targeting siRNA or FBXL4 siRNA and the efficiency of FBXL4 siRNA was evaluated using quantitative-PCR. Bars represent the mean ± SD from three independent transfections.

Data information: Scale bars = 20 µm.

© 2023 The Authors

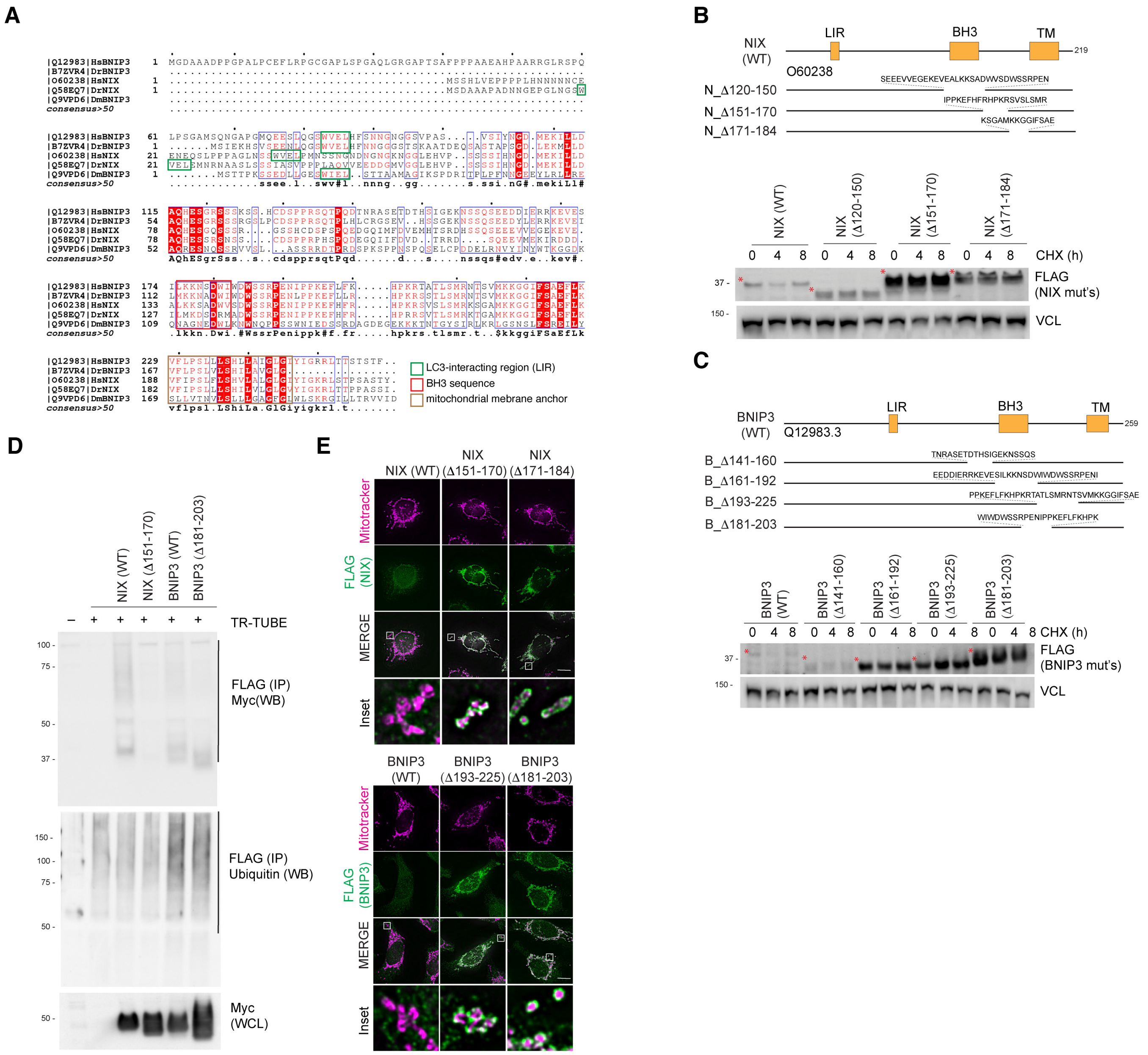

Figure EV2. Mapping the instability regions of NIX and BNIP3 to generate hyper-stable NIX and BNIP3.

A *Alignment of NIX and BNIP3 orthologues outlining conserved residues*. The LC3-interacting region, non-canonical BH3 region and the trans-membrane domains are shown. The blue boxes represent regions of conservation. White letters on a red background are strictly conserved, and red letters are highly conserved.

B *Deletion of regions from the C-terminus of NIX results in its stabilisation compared with wild-type NIX*. Hela-Flp-In NIX knockout cells expressing inducible FLAG-NIX-WT, FLAG-NIXD120-150, FLAG-NIXD151-170 and FLAG-NIXD171-184 were treated with cycloheximide (CHX) for the indicated times. Cells were then lysed and analysed by immunoblotting. Red asterisks denote the size of NIX or its deletion mutants.

C *Deletion of regions from the C-terminus of BNIP3 results in its stabilisation compared with wild-type BNIP3*. HeLa Flp-in BNIP3 knockout cells expressing inducible FLAG-BNIP3-WT or the specified FLAG-BNIP3 deletions mutants were treated with CHX. Red asterisks denote the size of BNIP3 or its deletion mutants.

D *Hyper-stable NIX and BNIP3 are ubiquitylated to a lesser extent than wild-type NIX and BNIP3*. HeLa NIX/BNIP3 DKO cells were co-transfected with FLAG-tagged TR-TUBE and myc-tagged NIX/BNIP3 constructs: NIX(WT), hyper-stable NIXΔ151-170, BNIP3(WT) or hyper-stable BNIP3(Δ181-203). FLAG-tagged TUBE protein was affinity purified from the corresponding lysates using FLAG-beads, and the precipitates were analysed by immunoblotting using antibodies to detect myc. The ladder of signal in the wild-type NIX and BNIP3 lanes corresponds to polyubiquitylated myc-BNIP3 or myc-NIX.

E *FLAG-NIX/BNIP3 and deletion mutants localise to the mitochondria*. HeLa-Flp-in cells expressing inducible FLAG-NIX/BNIP3-WT or deletion mutants were stained for MitoTracker (magenta) and FLAG antibodies (green). Scale bars = 20 mm.

 © 2023 The Authors

Figure EV3. FBXL4 deficiency promotes mitophagy through NIX/BNIP3 stabilisation.

A FBXL4-deficient cells are ultra-sensitive to DFP-induced mitophagy. U2OS mt-Keima cells and U2OS mt-Keima FBXL4 KO cells were treated with DFP at specified concentrations for 24 h and analysed by live-cell confocal microscopy. The emission signals obtained after excitation with the 458 nm laser (neutral pH) or 561 nm laser (acidic pH) are shown in green and magenta respectively.

B Quantification of mitophagy shown in panel (A). Mitophagy is represented as the ratio of mt-Keima 561 nm fluorescence intensity divided by mt-Keima 458 nm fluorescence intensity for individual cells normalised to the parental condition.

C Corresponding conditions from (A, B) were harvested for immunoblotting to analyse levels of NIX and BNIP.

D Analysis of NIX and BNIP localisation in parental cells or FBXL4 KO cells treated with specified concentration of DFP.

E Quantification of the MT-CO2 protein levels in parental or FBXL4 KO cells treated with DFP. Western blotting was used to assess MT-CO2 levels remaining relative to parental untreated cells.

F *Mitophagy induced by hyper-stable NIX (Δ151-170) requires NIX's LC3 interaction domain.* Hela Flp-in Keima cells stably expressing inducible NIX(WT), NIX(Δ151-170) or NIX (151–170, LIRmut) were treated with doxycycline for 48 h. Mitophagy was quantified as in (B).

Data information: In (B and F), translucent grey dots represent measurements from individual cells. In (B), coloured circles represent the mean ratio from independent experiments (N = 4 for Veh Ctrl conditions and N = 3 for other conditions) and the centre lines and bars represent the mean of the independent replicate means ± SD. In (E), the coloured circles represent the = densitometry values of MT-CO2 normalised to VCL loading control from independent experiments. In (F), the centre lines and bars represent the mean of the individual cells ± SD (N = 1, > 60 cells analysed). For (B), P values were calculated from the mean values from independent experiments using one-way ANOVA; for (F), P values were calculated from the ratios of the individual cells (*$P < 0.05$, **$P < 0.005$, ***$P < 0.001$, ****$P < 0.0001$). Scale bars = 20 μm. Source data are available online for this figure.

© 2023 The Authors

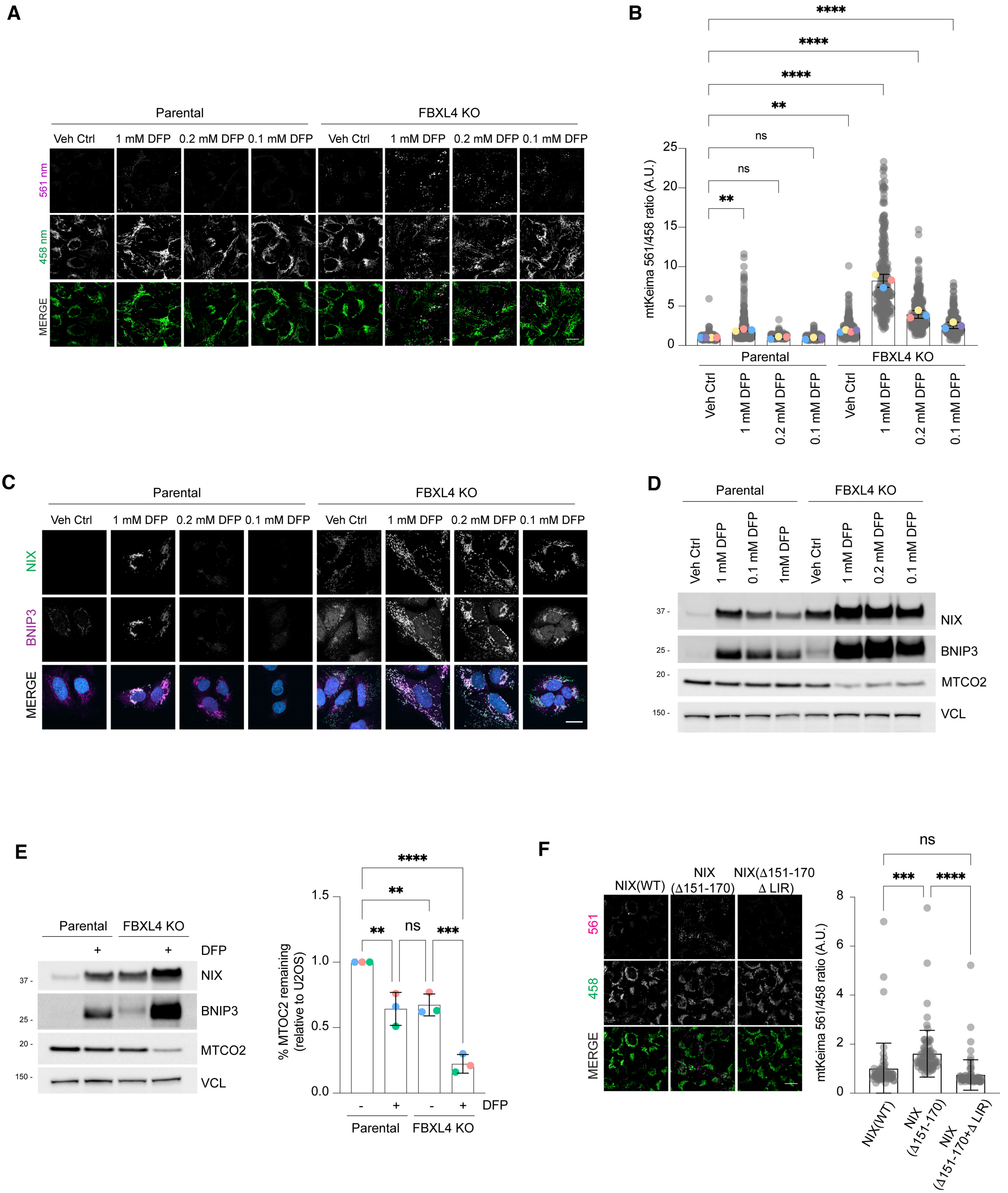

Figure EV3.

 © 2023 The Authors

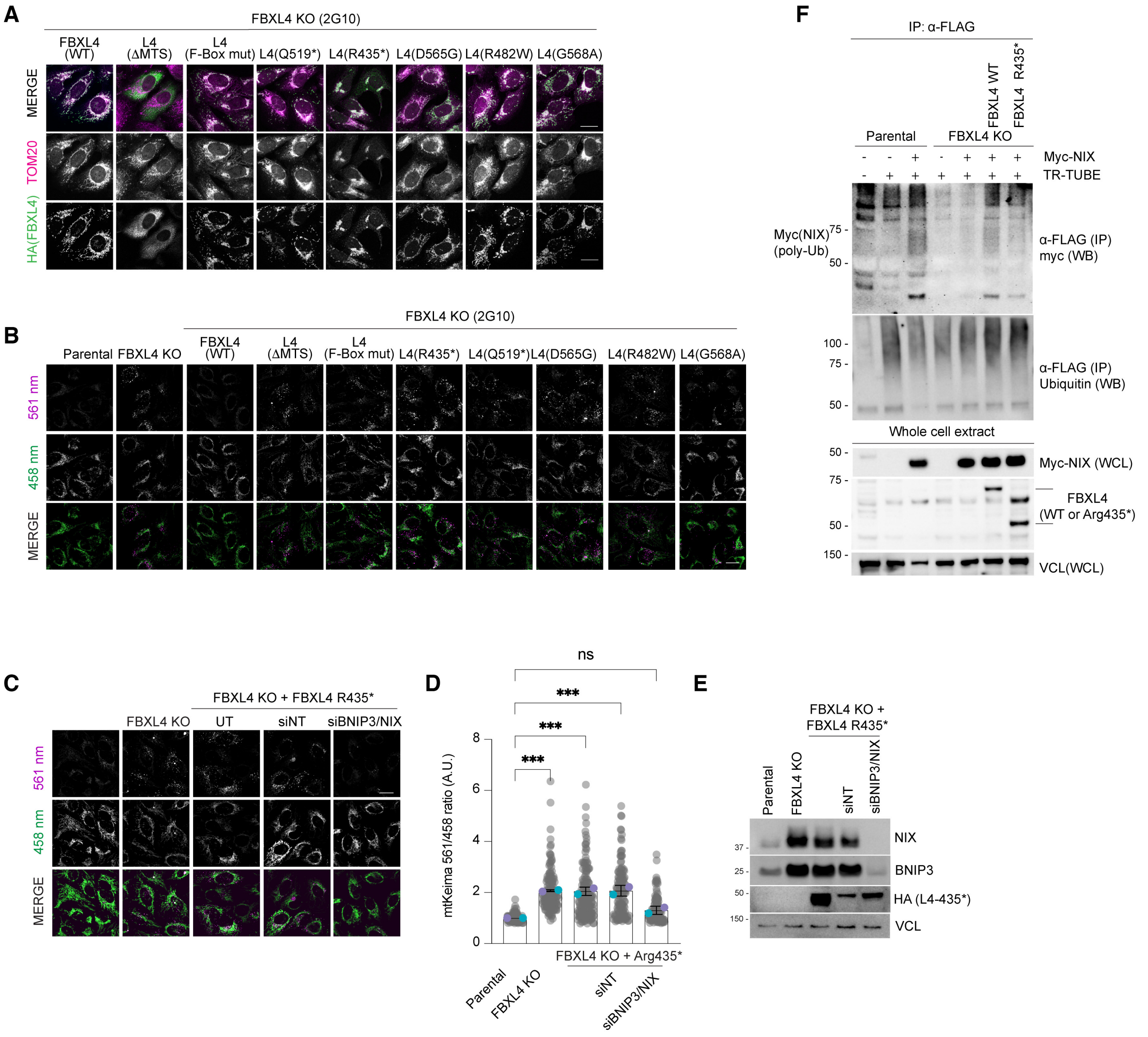
A
FBXL4 KO (2G10)
FBXL4 (WT)
L4 (ΔMTS)
L4 (F-Box mut)
L4(Q519*)
L4(R435*)
L4(D565G)
L4(R482W)
L4(G568A)
MERGE
TOM20
HA(FBXL4)
B
FBXL4 KO (2G10)
Parental
FBXL4 KO
FBXL4 (WT)
L4 (ΔMTS)
L4 (F-Box mut)
L4(R435*)
L4(Q519*)
L4(D565G)
L4(R482W)
L4(G568A)
561 nm
458 nm
MERGE
C
FBXL4 KO + FBXL4 R435*
FBXL4 KO
UT
siNT
siBNIP3/NIX
561 nm
458 nm
MERGE
D
ns
***
***
***
mtKeima 561/458 ratio (A.U.)
0
2
4
6
8
FBXL4 KO + Arg435*
Parental
FBXL4 KO
siNT
siBNIP3/NIX
E
FBXL4 KO + FBXL4 R435*
Parental
FBXL4 KO
siNT
siBNIP3/NIX
37 -
25 -
50 -
150 -
NIX
BNIP3
HA (L4-435*)
VCL
F
IP: α-FLAG
FBXL4 WT
FBXL4 R435*
Parental
FBXL4 KO
Myc-NIX
TR-TUBE
Myc(NIX)
(poly-Ub)
75 -
50 -
α-FLAG (IP)
myc (WB)
100 -
75 -
50 -
α-FLAG (IP)
Ubiquitin (WB)
Whole cell extract
50 -
Myc-NIX (WCL)
75 -
50 -
FBXL4
(WT or Arg435*)
150 -
VCL(WCL)

Figure EV4.

◀ **Figure EV4. MTDPS13 patient-derived FBXL4 variants do not efficiently assemble into an SCF complex and have impaired abilities to mediate NIX and BNIP3 turnover.**

A *Localisation of FBXL4 variants*. FBXL4 KO cells expressing FBXL4-HA wild-type or specified variants were fixed and stained for HA (to detect FBXL4 in green) or TOM20 (in magenta).

B *FBXL4 patient-derived variants exhibit reduced efficiency in mediating the suppression of mitophagy compared to wild-type FBXL4*. U2OS mt-Keima cells, U2OS mt-Keima FBXL4 KO cells and U2OS mt-Keima FBXL4 KO cells rescued with FBXL4 constructs were analysed by confocal microscopy. The emission signals obtained after excitation with the 458 nm laser (neutral pH) or 561 nm laser (acidic pH) are shown in green and magenta respectively. Quantification of these conditions is shown in Fig 4C.

C *Elevated mitophagy in FBXL4 KO cells expressing FBXL4Arg435* truncation variant is reduced when NIX/BNIP3 are depleted using siRNA*. FBXL4 KO cells stably expressing FBXL4Arg435* were transfected with siRNAs targeting both NIX and BNIP3 (NIX/BNIP3 si) or non-targeting siRNA (NT si). Live-cell confocal microscopy was performed to visualise mitophagy.

D Quantification of mitophagy shown in panel (C). Translucent grey dots represent measurements from individual cells. Coloured circles represent the mean ratio from independent experiments ($N = 2$). The centre lines and bars represent the mean of the independent replicates ± SD. At least 50 cells were analysed per condition, and over 100 cells were analysed in total per condition. *P* values were calculated based on the mean values using a one-way ANOVA (*$P < 0.05$, **$P < 0.005$, ***$P < 0.001$, ****$P < 0.0001$).

E Evaluation of the extent of NIX/BNIP3 depletion in (C, D) using Western blotting.

F *FBXL4Arg435* truncation variant is less efficient than wild-type FBXL4 at promoting NIX ubiquitylation*. Parental, FBXL4 KO cells, FBXL4 KO cells expressing either wild-type FBXL4 or FBXL4-Arg435* were transfected with TR-TUBE and myc-NIX, as indicated. Cell lysates obtained 48 h post-transfection were immunoprecipitated with anti-FLAG antibody, and the immunoprecipitates were analysed by immunoblotting using anti-myc antibody (to detect ubiquitylated NIX or BNIP3). The line on the left marks a ladder of bands corresponding to polyubiquitylated myc-BNIP3 or myc-NIX.

Data information: Scale bars = 20 μm.
Source data are available online for this figure.

 © 2023 The Authors
