# Supplementary material for: FBXL4 suppresses mitophagy by restricting the accumulation of NIX and BNIP3 mitophagy receptors
Source: EMBO J. 2023 May 10;42(13):e112767. doi: 10.15252/embj.2022112767 (PMC10308361; doi:10.15252/embj.2022112767)
Supplement: Supplementary file 9 — Appendix [file EMBJ-42-e112767-s002.pdf]

Appendix Figure S1

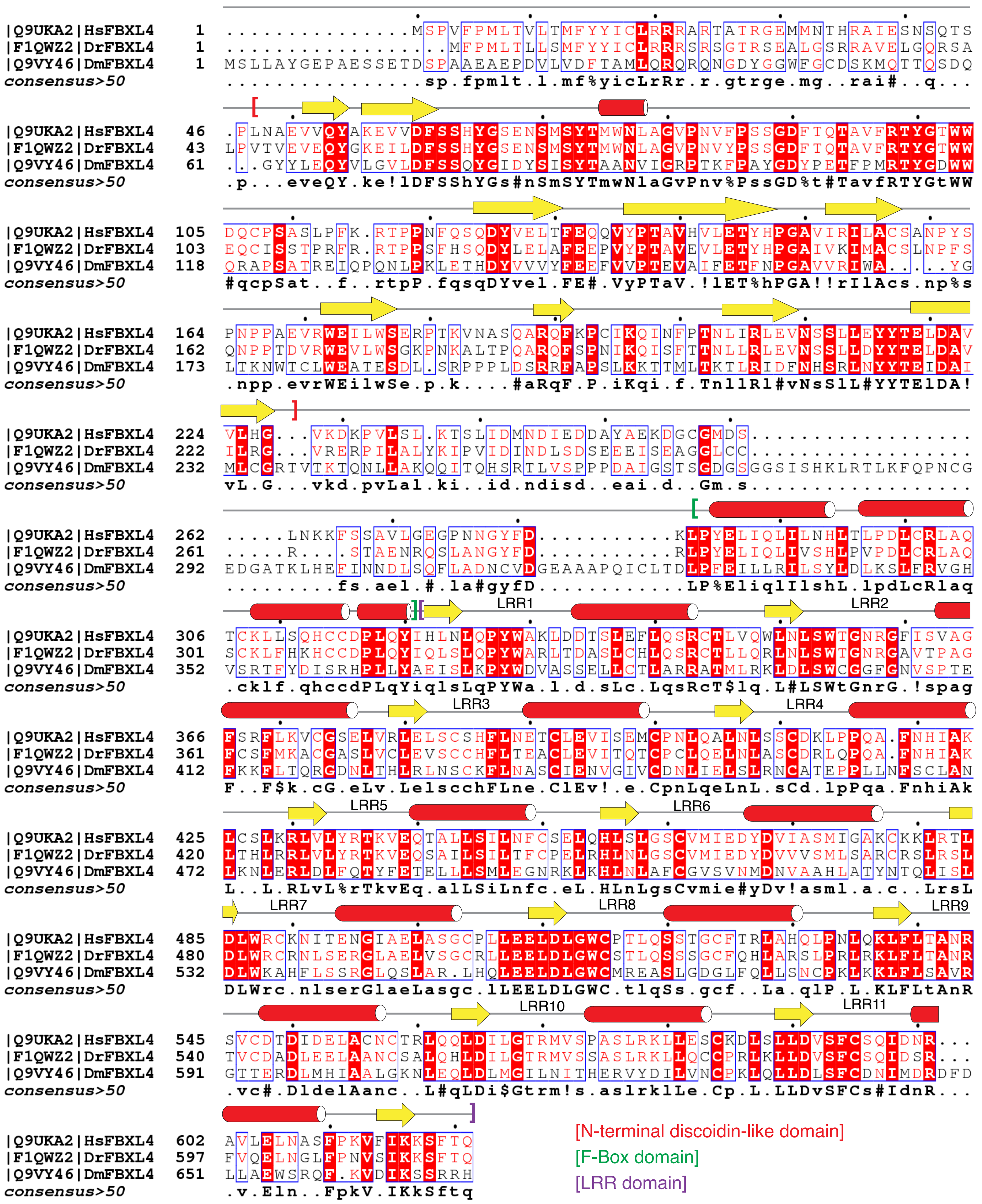

Appendix Figure S1. Alignment of FBXL4 orthologues outlining conserved residues and secondary structures. Red and white letters are strictly conserved, and red letters are highly conserved.
