# Supplementary material for: FBXL4 suppresses mitophagy by restricting the accumulation of NIX and BNIP3 mitophagy receptors
Source: EMBO J. 2023 May 10;42(13):e112767. doi: 10.15252/embj.2022112767 (PMC10308361; doi:10.15252/embj.2022112767)
Supplement: Supplementary file 10 [file EMBJ-42-e112767-s009.pdf]

THE EMBO JOURNAL

FBXL4 suppresses mitophagy by restricting the accumulation of NIX and BNIP3 mitophagy receptors

Julia Pagan, Giang Nguyen-Dien, Keri-Lyn Kozul, Yi Cui, Brendan Townsend, Soo Siang Ooi, Michele Pagano, Michael Lazarou, Robert Taylor, Brett Collins, Robert Parton, Prajakta Kulkarni, Nissa Carrodus, Steven Zuryn, Sean Millard, Antonio Marzio, and Mathew Jones
DOI: 10.15252/embj.2022112767

Corresponding author(s): Julia Pagan (j.pagan@uq.edu.au)

Review Timeline:

Submission Date:	11th Oct 22
Editorial Decision:	24th Nov 22
Revision Received:	23rd Mar 23
Editorial Decision:	17th Apr 23
Revision Received:	19th Apr 23
Accepted:	20th Apr 23

Editor: Hartmut Vodermaier

Transaction Report:

(Note: With the exception of the correction of typographical or spelling errors that could be a source of ambiguity, letters and reports are not edited. Depending on transfer agreements, referee reports obtained elsewhere may or may not be included in this compilation. Referee reports are anonymous unless the Referee chooses to sign their reports.)

Dr. Julia Pagan
University of Queensland
Otto Building
QLD 4067
Australia

24th Nov 2022

Re: EMBOJ-2022-112767
FBXL4 suppresses mitophagy by restricting the accumulation of NIX and BNIP3 mitophagy receptors

Dear Julia,

Thank you again for submitting your study on FBXL4 as suppressor of basal mitophagy for our consideration. I have now heard back from three expert referees, whose reports you will find copied below. Since all referees consider your findings interesting and important, we shall be happy to offer publication of an adequately revised version in The EMBO Journal.

As you will see, while referees 1 and 2 both request a limited number of experiments to strengthen the support for the conclusions of the study, referee 3 raises a somewhat longer list of concerns. I realize that not all of these issues would absolutely need to be addressed and that some may also fall beyond the scope of the present study. However, given that it is our policy to consider only a single round of major revision, it would be helpful to discuss what could and what could not be done already during the early stages of the revision period. I would thus invite you to email me a tentative point-by-point response as soon as you will have had a chance to go through the reports together with your coworkers - we could then discuss this revision proposal further via email of online call to define the key aspects of the revision work.

I should add that we could also offer extension of the default three-months revision period if needed, and that competing/overlapping work appearing here or elsewhere in the meantime will have no negative impact on our final decision on your study.

Detailed information on preparing, formatting and uploading a revised manuscript can be found below and in our Guide to Authors. Thank you again for the opportunity to consider this work for The EMBO Journal, and I look forward to hearing from you in due time.

With kind regards,

Hartmut

Hartmut Vodermaier, PhD
Senior Editor, The EMBO Journal
h.vodermaier@embojournal.org

*** PLEASE NOTE: All revised manuscript are subject to initial checks for completeness and adherence to our formatting guidelines. Revisions may be returned to the authors and delayed in their editorial re-evaluation if they fail to comply to the following requirements (see also our Guide to Authors for further information):

1) Every manuscript requires a Data Availability section (even if only stating that no deposited datasets are included). Primary datasets or computer code produced in the current study have to be deposited in appropriate public repositories prior to resubmission, and reviewer access details provided in case that public access is not yet allowed. Further information: embopress.org/page/journal/14602075/authorguide#dataavailability

2) Each figure legend must specify
- size of the scale bars that are mandatory for all micrograph panels
- the statistical test used to generate error bars and P-values
- the type error bars (e.g., S.E.M., S.D.)
- the number (n) and nature (biological or technical replicate) of independent experiments underlying each data point
- Figures may not include error bars for experiments with n<3; scatter plots showing individual data points should be used instead.

3) Revised manuscript text (including main tables, and figure legends for main and EV figures) has to be submitted as editable

text file (e.g., .docx format). We encourage highlighting of changes (e.g., via text color) for the referees' reference.

4) Each main and each Expanded View (EV) figure should be uploaded as individual production-quality files (preferably in .eps, .tif, .jpg formats). For suggestions on figure preparation/layout, please refer to our Figure Preparation Guidelines: http://bit.ly/EMBOPressFigurePreparationGuideline

5) Point-by-point response letters should include the original referee comments in full together with your detailed responses to them (and to specific editor requests if applicable), and also be uploaded as editable (e.g., .docx) text files.

6) Please complete our Author Checklist, and make sure that information entered into the checklist is also reflected in the manuscript; the checklist will be available to readers as part of the Review Process File. A download link is found at the top of our Guide to Authors: embopress.org/page/journal/14602075/authorguide

7) All authors listed as (co-)corresponding need to deposit, in their respective author profiles in our submission system, a unique ORCiD identifier linked to their name. Please see our Guide to Authors for detailed instructions.

8) Please note that supplementary information at EMBO Press has been superseded by the 'Expanded View' for inclusion of additional figures, tables, movies or datasets; with up to five EV Figures being typeset and directly accessible in the HTML version of the article. For details and guidance, please refer to: embopress.org/page/journal/14602075/authorguide#expandedview

9) Digital image enhancement is acceptable practice, as long as it accurately represents the original data and conforms to community standards. If a figure has been subjected to significant electronic manipulation, this must be clearly noted in the figure legend and/or the 'Materials and Methods' section. The editors reserve the right to request original versions of figures and the original images that were used to assemble the figure. Finally, we generally encourage uploading of numerical as well as gel/blot image source data; for details see: embopress.org/page/journal/14602075/authorguide#sourcedata

At EMBO Press, we ask authors to provide source data for the main manuscript figures. Our source data coordinator will contact you to discuss which figure panels we would need source data for and will also provide you with helpful tips on how to upload and organize the files.

Further information is available in our Guide For Authors: https://www.embopress.org/page/journal/14602075/authorguide

In the interest of ensuring the conceptual advance provided by the work, we recommend submitting a revision within 3 months (22nd Feb 2023). Please discuss the revision progress ahead of this time with the editor if you require more time to complete the revisions. Use the link below to submit your revision:

Link Not Available

Referee #1:

This a review of the manuscript by Nguyen-Dien, Kozul, Cui et al., however, another manuscript by different authors was also co-submitted and reviewed. Although both manuscripts will be reviewed separately, the rationale and significance are the same for both. The manuscripts show that FBXL4 regulates mitophagy via turnover of the mitochondrial membrane proteins NIX and BNIP3. Both NIX and BNIP3 are well known regulators of mitophagy, and FBXL4 has also been shown to negatively regulate this process, yet the link between FBXL4 mitophagy and BNIP3/NIX had not been previously made. This discovery is important for two reasons as BNIP3/NIX-dependent mitophagy was previously thought to be regulated largely by transcriptional upregulation but also that fact that FBXL1 is mutated in a mitochondrial-related disease and the discovery of a BNIP3/NIX involvement suggests a potential therapeutic pathway. Therefore, these works are exciting and publishable.

The Pagan group have produced a very strong manuscript that uncovers mechanistic detail on how FBXL4 downregulates BNIP3 and NIX to supress mitophagy. The data are clear and convincing, and I only have some minor suggestions.

Points to consider
1) Do the NIX/BNIP3 hyperstable mutants (Fig. EV3) have reduced ubiquitylation (e.g., via the TUBE assay) and/or reduced interaction with FBXL4?
2) In Figure 4F, the authors use patient-derived fibroblasts to show NIX/BNIP3 levels. However, it would be helpful to compare levels with fibroblasts from "healthy" individuals. Given the heterogeneity in expression with primary samples, are the levels of

NIX/BNIP3 abnormally high in the patient samples?
3) Does the expression of the disease-associated FBXL4 variants (Figure 4) affect NIX/BNIP3 binding and/or ubiquitylation?
4) The authors use DMOG in Fig EV1 but do not discuss this in the text. Also, figs EV1D-F do not appear to be discussed in the text?
5) Are the authors able to detect endogenous FBXL4 with an antibody? If not, perhaps this should be stated.
6) Scale bars are missing in some images (or mislocalised - Fig1H) and should be added.

Referee #2:

In this manuscript, Nguyen-Dien et al. report the role of FBXL4, a mitochondria-localized F-box protein, in negatively regulating mitochondria-specific autophagy (mitophagy). Previous findings reveal that mammalian cells undergo mitophagy in response to a variety of intra- and extracellular cues including hypoxia, iron depletion, and mitochondrial depolarization. In contrast to these inducible events whose molecular mechanisms have extensively been investigated, it remains largely unclear whether mitophagy occurs under basal conditions, and if so, how the appropriate level is established. In this study, the authors focused on NIX and BNIP3, two closely related BH3-only family proteins acting in hypoxia- and iron-depletion-induced mitophagy, and found that under normal conditions, the levels of these mitochondrial outer membrane-anchored proteins are increased in cells down-regulating or lacking FBXL4, a component of the SCF (SKP1-CUL1-F-box protein) ubiquitin E3 ligase complex. This FBXL4-mediated regulation to NIX and BNIP3 depends on its mitochondrial localization and F-box domain (required for its interaction with SKP1 and CUL1), further supporting the idea that the SCF complex containing FBXL4 promotes their turnover by the ubiquitin-proteasome system. The authors further demonstrated that NIX and BNIP3 are ubiquitylated in a manner dependent on FBXL4, and that loss of FBXL4 or stabilization of NIX/BNIP3 leads to acceleration of mitophagy. Finally, they analyzed pathogenic FBXL4 mutants associated with mitochondrial DNA depletion syndrome 13 (MTDPS13) and found that the MTDPS13-derived variants do not stably form a complex with SKP1 and CUL1, resulting in inefficient suppression of NIX/BNIP3 accumulation and mitophagy. Collectively, these data raise the possibility that FBXL4 contributes to steady-state mitophagy restraint via NIX/BNIP3 degradation, which could be physiologically relevant for mitochondrial homeostasis.

The authors convincingly identified for the first time NIX and BNIP3 as mitochondrial targets of the FBXL4-containing SCF complex. The findings in this study are potentially interesting and could provide new insights into the molecular mechanisms negatively regulating NIX/BNIP3-mediated mitophagy in mammals. However, it remains unclear if the FBXL4-containing SCF complex directly ubiquitylates NIX and BNIP3 (ideally analyzed by ubiquitylation assays using purified proteins in vitro and mass spectrometry for identification of ubiquitylation sites). In addition, there is no evidence suggesting that dysregulated acceleration of NIX/BNIP3-mediated mitophagy is the primary cause of FBXL4-related MTDPS13 symptoms. In conclusion, this study would significantly be strengthened if the authors clarify these issues and address the following points.

Major points:
1. In Figure 1C, to ask whether NIX and BNIP3 are degraded by the ubiquitin-proteasome system, the authors should perform the same western blot analysis for cells treated with Echinomycin + MG132.

2. In Figure 2G, to confirm if NIX and BNIP3 are indeed ubiquitylated, the authors should perform western blotting of anti-FLAG-immunoprecipitated Myc-NIX and Myc-BNIP3 with anti-ubiquitin.

3. In Figure EV3, to clarify whether hyper-stable NIX and BNIP3 variants are poorly captured or ubiquitylated by the FBXL4-containing SCF complex, the authors should subject these mutants to the assays in Figure 2F and 2G.

4. In Figure 4D, to test if mitophagy acceleration in cells expressing MTDPS13-derived FBXL4 variants results from aberrant accumulation of NIX and BNIP3, the authors should perform the same assays with NIX/BNIP3 knock-down/knock-out.

Minor points:
1. In Figures 2E and EV2B, the authors should perform line-scan profiling of FBXL4-HA- and TOM20/TOM50-positive fluorescence signals.

2. The authors should correct the descriptions for figures in the text: line 126, Figure EV1D-F; line 147, Figure 1F; line 163, Figure EV2A; line 170, Figure EV3A; line 230, Figure EV3G-H.

Referee #3:

The manuscript by Nguyen-Dien et al describes novel regulation mechanism of BNIP3L/NIX- and BNIP3- receptor mediated mitophagy. The authors have found FBXL4, a F-box protein part of ubiquitin ligase complex, to be a negative regulator of selective mitophagy. They have demonstrated that upon FBXL4 depletion both BNIP3L/NIX and BNIP3 are accumulating in the

cells leading to increased mitophagy induction, suggesting that without proper ubiquitination both receptors do not get degraded thus leading to more extensive mitophagy. Further, authors demonstrate that pathogenic variant of FBXL4 that causes encephalopathic mtDNA depletion syndrome is unable to interact with E3 ligase complex thus unables adequate regulation of BNIP3L/NIX and BNIP3-mediated mitophagy. Although the study is highly interesting and brings novelty to receptor-mediated mitophagy field, I find the manuscript not ready for acceptance to this journal in the presented format.
Comments:
1) The authors produced either FBXL4 knockout (KO) or knockdown (KD) cells but did not show any paadditionallyhe KO/KD was successful. I find it essential to show that protein is indeed not being produced in these cells: anti-FBXL4 staining is needed.
2) The authors use mtKeima to monitor mitophagy that has been widely used, but LC3 at the mitochondria, under treatments performed throughout the manuscript, should be presented as independent method to screen BNP3L/NIX and BNIP3 dependent mitophagy.
3) Furthermore, the manuscript would be additionaly improved if not just the initiation of mitophagy, but the mitophagy flux (progression) would be monitored (e.g. BafA1 treatement).
4) In general, immunofluorescent images presented are of poor quality and is difficult to observe the differences under different conditions. The authors do present quantifications, however in many instances they are not complementing what is shown i representative ImF images (e.g. F1A, 3A, 3D, 3G,...).
5) In M&M two different BNIP3L/NIX antibodies are listed. It is not clear which one was used in which experiments (wb and imf). Since antibodies are raised against different epitopes and due to high sequence and structural homology to BNIP3 it is important to show that antibodies are not cross-reacting.
6) Authors should show that accumulation of BNIP3L/NIX and BNIP3 upon FBXL4 depletion is at the mitochondria - mitochondrial vs. cytosolic fractions should be presented.
7) In Fig 1A and 1C the same treatment should be shown - there are no single treatments in 1C.
8) Fig EV1D should show BNIP3L/NIX and BNIP3 staining (to prove the presence of the proteins on mitochondria (to complement wb in fig ev1f).
9) In F2F protein size marker is not indicated.
10) In F2G it looks like both Ub and BNIP3L/NIX and BNIP3 are Myc labelled. If so, different tags should be used. What is Flag-tagged?
11) Fig 3A and 3B should complement each other - in F3A rescue is not visible.
12) In Fig 3G BNIP3L/NIX and BNIP3 staining should be presented.
13) How do authors explain that BNIP3L/NIX delta mutants are of higher molecular weight than wt?
14) In EV3D there is low expression of BNIP3 wt and delta141-160 construct. This should be commented.
15) It would be very informative to test LC3 levels in patient fibroblast to measure mitophagy/autophagy levels.
16) Have authors examined LIR mutants/LIR phosphorylation mutants or even TM mutants?
17) Did authors performed experiments to block proteasome activity? What is the phenotype of BNIP3L/NIX and BNIP3 in FBXL4 KO when proteasome activity is blocked?
Additional comments:
Recent work by Wilhelm et al, 2022, EMBO journal, have shown that BNIP3L/NIX is needed for pexophagy (similar treatments were used) - it would be great addition to check if FBXL4 influences BNIP3L/NIX- mediated pexophagy in the similar manner.

Response to reviewers (Manuscript EMBOJ-2022-112767)

Author responses in blue text.

We thank each reviewer for their time and insightful suggestions. We include the following new figures: 1D, 1G, EV1F-G, EV2D, EV3C, EV3E-F, 4G-I, EV4C-F.

Referee #1:

This a review of the manuscript by Nguyen-Dien, Kozul, Cui et al., however, another manuscript by different authors was also co-submitted and reviewed. Although both manuscripts will be reviewed separately, the rationale and significance are the same for both. The manuscripts show that FBXL4 regulates mitophagy via turnover of the mitochondrial membrane proteins NIX and BNIP3. Both NIX and BNIP3 are well known regulators of mitophagy, and FBXL4 has also been shown to negatively regulate this process, yet the link between FBXL4 mitophagy and BNIP3/NIX had not been previously made. This discovery is important for two reasons as BNIP3/NIX-dependent mitophagy was previously thought to be regulated largely by transcriptional upregulation but also that fact that FBXL1 is mutated in a mitochondrial-related disease and the discovery of a BNIP3/NIX involvement suggests a potential therapeutic pathway. Therefore, these works are exciting and publishable.

The Pagan group have produced a very strong manuscript that uncovers mechanistic detail on how FBXL4 downregulates BNIP3 and NIX to supress mitophagy. The data are clear and convincing, and I only have some minor suggestions.

1) Do the NIX/BNIP3 hyperstable mutants (Fig. EV3) have reduced ubiquitylation (e.g., via the TUBE assay) and/or reduced interaction with FBXL4?

RESPONSE: These are important questions raised by the reviewer. Accordingly, we conducted the TR-TUBE assay on the hyper-stable mutants of NIX (NIXΔ151-170) and BNIP3 (BNIP3Δ181-203) to investigate whether they have reduced ubiquitylation compared to their wild-type counterparts. Our results demonstrate that the levels of ubiquitylation of NIXΔ151-170 and BNIP3Δ181-203 were lower than those of their respective wild-type proteins, despite being expressed at higher levels (Figure EV2D). Therefore, we conclude that the increased stability of NIXΔ151-170 and BNIP3Δ181-203 is due to their impaired ubiquitylation. It is worth noting that the deleted regions in NIX and BNIP3 contain several lysine residues, including one that have previously been reported to be modified by ubiquitin, such as Lys154 of NIX (detected after MG132 treatment, Phosphosite).

There are several potential causes for the increased stability of the NIX and BNIP3 deletion mutants: 1) loss of ubiquitylation sites, and/or 2) loss of FBXL4 binding site, and/or 3) loss of post-translational modifications that support recruitment of FBXL4 to the ubiquitylation sites. The evidence of reduced ubiquitylation of the hyper-stable BNIP3/NIX mutants compared to wild type supports the explanation for the increased stability of these mutants. We did not attempt to demonstrate that the hyper-stable mutants have reduced binding to FBXL4 due to the time required to optimize affinity binding assays, and we felt that this experiment was beyond the scope of the current manuscript. However, we are currently collaborating with others to understand the binding interface and possible regulatory mechanisms that control the interaction.

2) In Figure 4F, the authors use patient-derived fibroblasts to show NIX/BNIP3 levels. However, it would be helpful to compare levels with fibroblasts from "healthy" individuals. Given the heterogeneity in expression with primary samples, are the levels of NIX/BNIP3 abnormally high in the patient samples?

We acknowledge the reviewer's suggestion to include a comparison between control fibroblast lines and patient-derived fibroblasts, and we would have ideally preferred to include such a comparison with a panel of control fibroblast lines (>3 lines) and a panel of patient-derived fibroblasts (>3 lines) to account for the known heterogeneity of expression levels between primary samples. However, we were unable to obtain research-consented appropriate age-matched/gender-matched control fibroblasts in a timely manner, or other FBXL4 lines for comparison.

To address whether elevated BNIP3 and NIX are responsible for the increased mitophagy in the FBXL4 patient cell lines, we have included data demonstrating that mitophagy is reduced in these patient cells after co-depletion of BNIP3 and NIX by siRNA (new **Figures 4H**), as suggested by reviewer 2, point 4. Therefore, we conclude that the increased mitophagy in FBXL4 patient cells is mediated via NIX and BNIP3. Although we could not compare with control fibroblasts due to difficulties obtaining appropriate control fibroblasts, our findings indicate that the enhanced mitophagy in the FBXL4 deficient cells can be rescued either by restoration of FBXL4 or co-depletion of NIX/BNIP3.

3) Does the expression of the disease-associated FBXL4 variants (Figure 4) affect NIX/BNIP3 binding and/or ubiquitylation?

RESPONSE: We demonstrated that FBXL4 pathological variants are inefficient at suppressing mitophagy and mediating BNIP3/NIX turnover. To show that the ubiquitylation of NIX/BNIP3 is diminished in the context of MTDPS13, we performed a TR-TUBE assay in fibroblasts derived from the FBXL4-variant patient. We found that the ubiquitylation smear of NIX was only detectable in the patient-derived fibroblasts that had been transduced with wild-type FBXL4 to restore FBXL4 function (**Figure 4I**). This suggests that the higher levels of NIX in the patient cells is likely due to its reduced ubiquitylation. Additionally, we performed the TR-TUBE rescue experiments using the FXBL4R435* variant, finding partial reduction in the ubiquitylation of myc-tagged NIX, further suggesting that the patient-derived variant has reduced ability to restore NIX ubiquitylation (**Figure EV4F**).

As mentioned in response to point 1, we are working with collaborators to understand the binding interface and possible regulatory mechanisms controlling the ligase-substrate interaction, consequently we have not broached this interaction in the current manuscript.

4) The authors use DMOG in Fig EV1 but do not discuss this in the text. Also, figs EV1D-F do not appear to be discussed in the text?

To better explain these results, we have edited the initial results section of the text and rearranged the figures as follows:

- Moved EV Figure 1 D-F to main figure 1 A-D (including immunofluorescence to show accumulation of NIX and BNIP3 at mitochondria as requested by reviewer 3).
- Clarified text of the final paragraph on page 4, as follows:

Firstly, we inhibited the entire CRL-ubiquitin ligase family using MLN4924, an inhibitor of Cullin neddylation (Soucy *et al*, 2009). We observed robust mitophagy (Figure 1A-B), along with an increase in NIX and BNIP3 levels (Figure C-D), similar to the effects seen with other HIF1α stabilisers like the iron chelator deferiprone (DFP) and prolyl hydroxylase inhibitor dimethyloxalylglycine (DMOG) (Allen *et al.*, 2013) (Figure 1A-D). We used CRISPR-Cas9 to create NIX/BNIP3 double knockout cells to investigate whether NIX and BNIP3 are essential for mitophagy in response to MLN4924, as is the case for mitophagy triggered by DFP (Wilhelm *et al*, 2022; Zhao *et al.*, 2020). We observed that mitophagy was diminished in the absence of NIX and BNIP3 in response to MLN4924, as well as the other HIF1α activators DMOG and DFP (Figure 1A-B).

5) Are the authors able to detect endogenous FBXL4 with an antibody? If not, perhaps this should be stated.

To address this point, we include a statement that there are currently no FBXL4 antibodies able to detect endogenous FBXL4 (page 6, line 156).

We tested multiple commercial FBXL4 antibodies (Sigma SAB2701256, Santa Cruz sc-376102, and Thermo Fisher pa530510), as well as our own in-house antibody generated against FBXL4 (Yenzym), but none of them were able to detect endogenous FBXL4. It is likely that FBXL4 is expressed at low levels in cells, particularly under basal conditions.

However, we were able to confirm the presence of endogenous FBXL4 at the protein level in our cells using BioID-mass spectrometry for NIX, finding FBXL4 in the interacting list for BirA-tagged NIX (personal communication, unpublished data).

6) Scale bars are missing in some images (or mislocalised - Fig1H) and should be added.

We apologise for these oversights and have corrected the missing or mis-localised scale bars.

Referee #2:

In this manuscript, Nguyen-Dien et al. report the role of FBXL4, a mitochondria-localized F-box protein, in negatively regulating mitochondria-specific autophagy (mitophagy). Previous findings reveal that mammalian cells undergo mitophagy in response to a variety of intra- and extracellular cues including hypoxia, iron depletion, and mitochondrial depolarization. In contrast to these inducible events whose molecular mechanisms have extensively been investigated, it remains largely unclear whether mitophagy occurs under basal conditions, and if so, how the appropriate level is established. In this study, the authors focused on NIX and BNIP3, two closely related BH3-only family proteins acting in hypoxia- and iron-depletion-induced mitophagy, and found that under normal conditions, the levels of these mitochondrial outer membrane-anchored proteins are increased in cells down-regulating or lacking FBXL4, a component of the SCF (SKP1-CUL1-F-box protein) ubiquitin E3 ligase complex. This FBXL4-mediated regulation to NIX and BNIP3 depends on its mitochondrial localization and F-box domain (required for its interaction with SKP1 and CUL1), further supporting the idea that the SCF complex containing FBXL4 promotes their turnover by the ubiquitin-proteasome system. The authors further demonstrated that NIX and BNIP3 are ubiquitylated in a manner dependent on FBXL4, and that loss of FBXL4 or stabilization of NIX/BNIP3 leads to acceleration of mitophagy. Finally, they analyzed pathogenic FBXL4 mutants associated with mitochondrial DNA depletion syndrome 13

(MTDPS13) and found that the MTDPS13-derived variants do not stably form a complex with SKP1 and CUL1, resulting in inefficient suppression of NIX/BNIP3 accumulation and mitophagy. Collectively, these data raise the possibility that FBXL4 contributes to steady-state mitophagy restraint via NIX/BNIP3 degradation, which could be physiologically relevant for mitochondrial homeostasis.

The authors convincingly identified for the first time NIX and BNIP3 as mitochondrial targets of the FBXL4-containing SCF complex. The findings in this study are potentially interesting and could provide new insights into the molecular mechanisms negatively regulating NIX/BNIP3-mediated mitophagy in mammals. However, it remains unclear if the FBXL4-containing SCF complex directly ubiquitylates NIX and BNIP3 (ideally analyzed by ubiquitylation assays using purified proteins in vitro and mass spectrometry for identification of ubiquitylation sites). In addition, there is no evidence suggesting that dysregulated acceleration of NIX/BNIP3-mediated mitophagy is the primary cause of FBXL4-related MTDPS13 symptoms. In conclusion, this study would significantly be strengthened if the authors clarify these issues and address the following points.

RESPONSE: We thank the reviewer for raising these important questions and address them in the relevant points below.

Major points:

1. In Figure 1C, to ask whether NIX and BNIP3 are degraded by the ubiquitin-proteasome system, the authors should perform the same western blot analysis for cells treated with Echinomycin + MG132.

RESPONSE: In response to the request, we conducted an experiment similar to the original Figure 1C (now Figure 1G) with MG132, however we had to exclude equivalent time points due to toxicity from MG132. The results showed that the levels of BNIP3/NIX, and HIF1α, increased upon treatment with MG132. To demonstrate that the accumulation of BNIP3 and NIX was independent of HIF1α (i.e., due to inhibition of FBXL4), we co-treated MG132 treated cells with echinomycin, a HIF1α inhibitor, and found that echinomycin did not reduce the levels of NIX and BNIP3 in response to MG132. Thus, MG132 (proteasome inhibitor) and MLN4924 (Cullin-RING ubiquitin ligase inhibitor) result in the accumulation of BNIP3 and NIX in the presence of echinomycin, suggesting that there is a Cullin-RING ligase involved in degradation of BNIP3 and NIX (as demonstrated in subsequent figures to be SCF-FBXL4).

While the findings support our conclusion, it is important to note that we cannot definitively conclude that HIF1α is not involved due to the assay's limited duration (3 hours). The short duration of MG132 treatment that we were able to use before observing cytotoxicity in U2OS cells is likely insufficient to induce significant BNIP3/NIX by HIF1α.

We would prefer to keep that figure in the "response to reviewer" file to simplify the text while still being available to interested readers. However, we are happy to move to the main article if preferred by the editor or the reviewer.

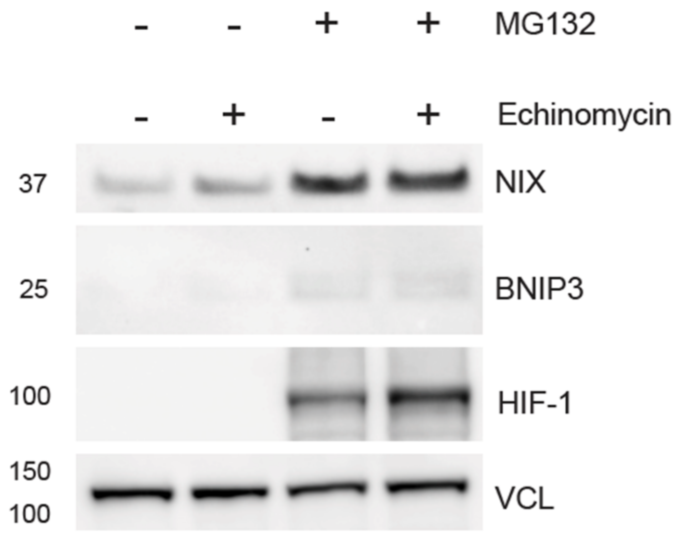

2. In Figure 2G, to confirm if NIX and BNIP3 are indeed ubiquitylated, the authors should perform western blotting of anti-FLAG-immunoprecipitated Myc-NIX and Myc-BNIP3 with anti-ubiquitin.

RESPONSE: The reviewer makes an excellent point, and we acknowledge that we have not demonstrated direct ubiquitylation of NIX/BNIP3 by FBXL4 using *in vitro* ubiquitylation experiments using purified components. The TR-TUBE (tandem-ubiquitin binding entity) assay used instead is a well-established method to detect ubiquitylated substrates involving the pull down of polyubiquitylated substrates in cells using TR-TUBE, followed by Western blotting with an antibody specific to the potentially ubiquitylated protein of interest (in this case, BNIP3 or NIX-myc-tagged). Although this is not an in vitro assay, it provides evidence that ubiquitylated NIX/BNIP3 in cells depends on the presence of FBXL4, supporting our hypothesis that FBXL4 is involved in the ubiquitylation and subsequent degradation of these proteins. In response to

advice from additional external readers, we have also revised our description of the TR-TUBE assay for clarity, since we did not explain it well in our original manuscript (page 7, last paragraph).

As requested, we have also added an anti-ubiquitin blot to several of our new TR-TUBE assays (Figures EV2D and EV4F), but it is important to note that this blot does not provide evidence of the specific ubiquitylation of myc-NIX and myc-BNIP3. Rather, it shows the loading input of ubiquitylated substrates captured by TR-TUBE.

To address the question of direct ubiquitylation by FBXL4, we performed pilot experiments attempting to reconstitute NIX/BNIP3 ubiquitylation in vitro however we were unsuccessful. These assays can be time-consuming to optimize and may require specific kinases or E2 enzymes for FBXL4 activity/binding, possibly requiring a baculovirus expression system, which we have not set up.

However, we were successful in demonstrating that the hyperstable variants of NIX and BNIP3 exhibited less ubiquitylation than their wild-type counterparts, suggesting that the ubiquitylation is required for their turnover (now shown in Figure EV2D). Notably, as mentioned in response to reviewer 1's comments, NIX and BNIP3 contain several lysine residues that have been reported to be modified with ubiquitin (Phosphosite). Furthermore, these residues fall within regions in BNIP3 and NIX that promote their turnover. We have referred to potential lysine residues in the text (page 8, line 232).

3. In Figure EV3, to clarify whether hyper-stable NIX and BNIP3 variants are poorly captured or ubiquitylated by the FBXL4-containing SCF complex, the authors should subject these mutants to the assays in Figure 2F and 2G.

RESPONSE: We now include data demonstrating that endogenous NIX is poorly ubiquitylated in the M11 Arg435*-derived patient fibroblasts, and that this ubiquitylation can be restored by complementation of wild-type FBXL4 (Figure EV4I). Furthermore, we include supporting data that the FBXL4-Arg435* truncation variant was not as proficient as wild-type FBXL4 at ubiquitylating NIX in rescue assays. Thus, we conclude that patient-derived variants in FBXL4 are poorly able to mediate NIX and BNIP3 ubiquitylation.

4. In Figure 4D, to test if mitophagy acceleration in cells expressing MTDPS13-derived FBXL4 variants results from aberrant accumulation of NIX and BNIP3, the authors should perform the same assays with NIX/BNIP3 knock-down/knock-out.

RESPONSE: We appreciate the reviewer's suggestion. In our initial submission, we presented evidence that depletion of NIX and BNIP3 by siRNA reduced the increased mitophagy observed in the FBXL4 knockout cell line. To confirm the necessity of NIX and BNIP3 for elevated mitophagy in the disease context, we have conducted additional experiments demonstrating that siRNA-mediated depletion of NIX/BNIP3 rescues the high mitophagy observed in patient-derived fibroblasts lacking FBXL4 (Figure 4H).

Minor points:
1. In Figures 2E and EV2B, the authors should perform line-scan profiling of FBXL4-HA- and TOM20/TOM50-positive fluorescence signals.

RESPONSE: We now include line profiling based on figure 2E, which reveals a distinct separation between the signals of TIM50 and FBXL4. In contrast, the signals of TOM20 and FBXL4 overlap.

2. The authors should correct the descriptions for figures in the text: line 126, Figure EV1D-F; line 147, Figure 1F; line 163, Figure EV2A; line 170, Figure EV3A; line 230, Figure EV3G-H.

RESPONSE: We have fixed these figure call-outs and apologise for any confusion caused.

Referee #3:

The manuscript by Nguyen-Dien et al describes novel regulation mechanism of BNIP3L/NIX- and BNIP3- receptor mediated mitophagy. The authors have found FBXL4, a F-box protein part of ubiquitin ligase complex, to be a negative regulator of selective mitophagy. They have demonstrated that upon FBXL4 depletion both BNIP3L/NIX and BNIP3 are accumulating in the cells leading to increased mitophagy induction, suggesting that without proper ubiquitination both receptors do not get degraded thus leading to more extensive mitophagy. Further, authors demonstrate that pathogenic variant of FBXL4 that causes encephalopathic mtDNA depletion syndrome is unable to interact with E3 ligase complex thus unables adequate regulation of BNIP3L/NIX and BNIP3-mediated mitophagy. Although the study is highly interesting and brings novelty to receptor-mediated mitophagy field, I find the manuscript not ready for acceptance to this journal in the presented format.

Comments:
1) The authors produced either FBXL4 knockout (KO) or knockdown (KD) cells but

did not show any additionally the KO/KD was successful. I find it essential to show that protein is indeed not being produced in these cells: anti-FBXL4 staining is needed.

RESPONSE: Regrettably, we have been unsuccessful in obtaining or creating an antibody suitable for detecting FBXL4 via either immunoblotting or immunofluorescence staining. We tested three commercially available FBXL4 antibodies (Sigma SAB2701256, Santa Cruz sc-376102, and Thermo Fisher pa530510), as well as an antibody we produced in-house, but none yielded positive results. It is possible that FBXL4 is expressed at low levels within cells. As suggested by Reviewer 1, we have added a statement to the manuscript indicating that there are presently no antibodies available for detecting endogenous FBXL4 (see page 6, line 15).

We have addressed the reviewer's question regarding the effectiveness of FBXL4 siRNA knockdown in depleting FBXL4 protein levels by transfecting FBXL4 siRNA into a stable cell line expressing exogenous FBXL4 tagged with HA (stable U2OS-FBXL4-HA-C). Our results demonstrate that transfection of FBXL4 siRNA efficiently downregulates exogenous FBXL4 protein levels (**Figure EV1F**). We also assessed the levels of FBXL4 transcript by q-PCR and found that transfection of FBXL4 siRNA significantly reduces FBXL4 transcript levels (Figure EV1G). Based on the similarity of the phenotypes induced by FBXL4 siRNA and CRISPR deletion, we are confident that both methods effectively deplete or knockout FBXL4 function in cells. This is supported by the increased levels of NIX and BNIP3 observed in both cases. Furthermore, to ensure that the FBXL4 gRNAs have no non-specific effects, we performed rescue experiments by complementing wild-type FBXL4 back into the FBXL4 CRISPR clones and were consistently able to rescue the levels of BNIP3 and NIX back down to base-line levels.

2) The authors use mtKeima to monitor mitophagy that has been widely used, but LC3 at the mitochondria, under treatments performed throughout the manuscript, should be presented as independent method to screen BNP3L/NIX and BNIP3 dependent mitophagy.

RESPONSE: The Mito-Keima fluorescence ratio is a sensitive and quantitative measure of mito-lysosomes in cells. Since we have not established a quantitative assay for LC3-mitochondria colocalisation, we instead now provide Western blot analysis to complement the mito-Keima assay and quantify mitochondrial protein degradation (MT-CO2 levels) as a read-out of mitophagy (Figure EV3E and 4F). Our findings are consistent with previous

studies, including by Alsina *et al* (10.15252/emmm.201911659), showing decreased levels of mitochondrial proteins in FBXL4-deficient cells, suggesting increased turnover.

3) Furthermore, the manuscript would be additionaly improved if not just the initiation of mitophagy, but the mitophagy flux (progression) would be monitored (e.g. BafA1 treatement).

RESPONSE: The mtKeima assay monitors mitochondrial delivery to lysosomes (keima in lysosomes is excited at acidic pH), thus is a read-out of end-stage mitophagy. We used BafA1 as a control when we were setting up the mtKeima assay in our group, demonstrating that the lysosomal mtKeima signal is largely abolished after BafA1 treatment. Thus, we are confident that we are measuring mitophagy flux using the mtKeima assay.

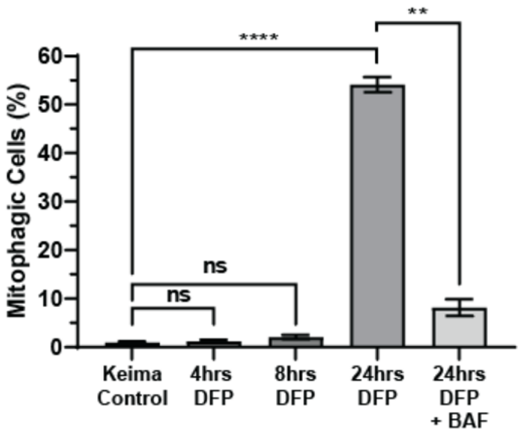

4) In general, immunofluorescent images presented are of poor quality and is difficult to observe the differences under different conditions. The authors do present quantifications, however in many instances they are not complementing what is shown i representative ImF images (e.g. F1A, 3A, 3D, 3G,...).

RESPONSE: We appreciate the reviewer's feedback and we have taken steps to improve the visualisation of mitophagy in our manuscript and changed the colour combinations. We have carefully reviewed our images and chosen representative cells and settings that ensure that the mitophagy is clearly distinguishable from the background. Furthermore, we have confirmed that the mitophagy is distinguishable in on various computer displays and when printed (we found it hard to visualise when printed). To facilitate better visualization, we have also included a document that presents various color

combinations for our microscopy images, which may aid certain individuals in discerning the mitophagy.

5) In M&M two different BNIP3L/NIX antibodies are listed. It is not clear which one was used in which experiments (wb and imf). Since antibodies are raised against different epitopes and due to high sequence and structural homology to BNIP3 it is important to show that antibodies are not cross-reacting.

We have conducted experiments to confirm that the BNIP3 and NIX antibodies used in our study do not cross-react with each other when applied in Western blotting and IF. Specifically, we used CRISPR KO clones for either BNIP3 or NIX, and tested the reciprocal antibody's ability to detect the other protein. Additionally, we provide equivalent data for immunofluorescence staining of NIX and BNIP3. The BNIP3 rabbit antibody recognises an extremely weak signal in the BNIP3 KO line but only after DFP treatment. Thus, the antibodies are very specific, and the conclusions of our manuscript are unchanged. We have specified in the applications in the M and M.

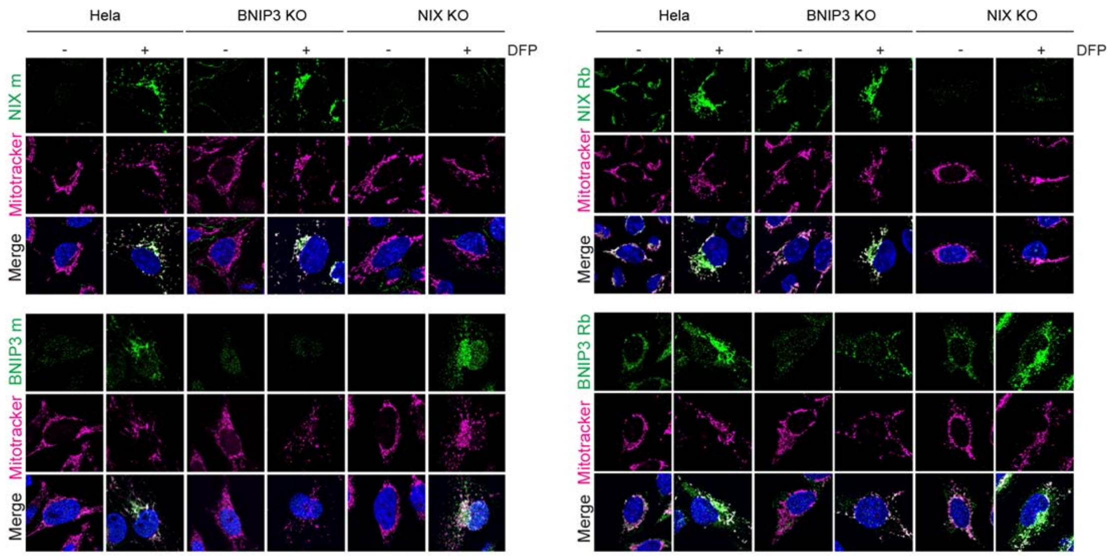

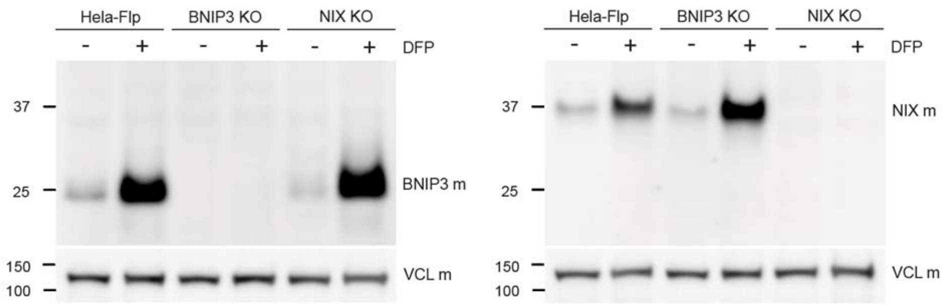

6) Authors should show that accumulation of BNIP3L/NIX and BNIP3 upon FBXL4 depletion is at the mitochondria - mitochondrial vs. cytosolic fractions should be presented.

Figures 2B, 2C, EV3C (FBXL4 CRISPR clones) and 1D (MLN4924 treatment) clearly demonstrate that loss of FBXL4 function leads to the accumulation of NIX and BNIP3 at mitochondria, as evidenced by co-staining with mitochondrial markers. While it is possible that NIX and BNIP3 may also accumulate in the cytosol, even fractionation experiments would not alter the conclusion that NIX/BNIP3 accumulate at mitochondria.

7) In Fig 1A and 1C the same treatment should be shown - there are no single treatments in 1C.

We have generated a Western blot figure to complement the microscopy with the same time points (now Figure 1G).

8) Fig EV1D should show BNIP3L/NIX and BNIP3 staining to prove the presence of the proteins on mitochondria (to complement wb in fig ev1f).

We have provided these images (now Figure 1D).

9) In F2F protein size marker is not indicated.

This has been corrected.

10) In F2G it looks like both Ub and BNIP3L/NIX and BNIP3 are Myc labelled. If so, different tags should be used. What is Flag- tagged?

RESPONSE: The TR-TUBE (Tandem Ubiquitin Binding Entity) protein used in our assay is FLAG-tagged and binds to poly-ubiquitylated substrates in cells, which includes all polyubiquitylated substrates. In this assay, FLAG-TR-TUBE is first precipitated using FLAG beads to pull-down polyubiquitylated substrates. The precipitation is then run on a gel and immunoblotting is performed to detect whether the protein of interest (in our case, myc-BNIP3 and myc-NIX) is ubiquitylated using an anti-myc antibody. The polyubiquitylation is visualized as a smear on the gel, which is detected by the myc antibody. To demonstrate the specificity of the signal, we show that the smear of ubiquitin (detected by myc) is reduced in FBXL4-deficient cells, indicating that FBXL4 regulates the ubiquitylation of these substrates. It is important to note that since TR-TUBE

pulls down all ubiquitylated proteins, anti-ubiquitin western blotting is not able to distinguish specific substrates. Additional details on the assay can be found in PMC4403176.

11) Fig 3A and 3B should complement each other - in F3A rescue is not visible.

This has been addressed as in point 4.

12) In Fig 3G BNIP3L/NIX and BNIP3 staining should be presented.

We have included these results in Figure EV3C.

13) How do authors explain that BNIP3L/NIX delta mutants are of higher molecular weight than the wt?

RESPONSE: It is possible that the slower-migrating deletion mutants in NIX are phosphorylated, as BNIP3 and NIX are known to be phosphoproteins and some phosphorylation sites promote mitophagy (Rogov *et al*). However, whether the phosphorylation is related to the stabilisation of the proteins or independently promotes mitophagy is currently unknown and beyond the scope of our manuscript. We are collaborating with others to investigate the regulatory modifications and binding interface between ligase and receptors.

14) In EV3D there is low expression of BNIP3 wt and delta141-160 construct. This should be commented.

RESPONSE: To clear up confusion about the experiment, we always observe that wild-type BNIP3 and NIX are expressed at low levels presumably since they are degraded by FBXL4-dependent mechanisms. The purpose of the experiments in EV3 (now EV2 and 3G-H) was to demonstrate that increasing the levels and stability of BNIP3 or NIX is enough to stimulate mitophagy in the absence of other signals, as opposed to wild type. The point was to investigate whether mitophagy in FBXL4 knockout cells could be replicated by the expression of hyper-stable mutants of BNIP3 and NIX. We have modified the text to eliminate ambiguity on this point.

Significantly, these experiments were carried out using the HeLa Flp-in system, which allowed us to evaluate the physiological levels of wild-type NIX and BNIP3 (i.e., wild-type BNIP3 and NIX levels were not significantly overexpressed compared to endogenous BNIP3 and NIX and do not induce

mitophagy in our system). This allowed us to directly compare the impact of stabilizing NIX and BNIP3 on mitophagy.

15) It would be very informative to test LC3 levels in patient fibroblast to measure mitophagy/autophagy levels.

RESPONSE: LC3 levels have previously been assessed in the same patient cell line in Alsina 2020 (Figure EV4). We found variable results when we performed Western blotting with patient fibroblasts therefore have not included it in the final figure. Instead, we look at MT-CO2 (mito degradation) and SQSTM (autophagy marker) in patient fibroblasts complemented with FBXL4 (**Figure 4G**).

16) Have authors examined LIR mutants/LIR phosphorylation mutants or even TM mutants?

RESPONSE: To address this request, we have included data demonstrating that the mitophagy driven by the expression of hyper-stable mutants of NIX is diminished when its LIR domain is mutated (Figure EV3G-H). Since this is not an unexpected result given other groups have shown similar results (Marinkovic 2021, Willhelm 2022) we only assessed NIX, but expect similar results for BNIP3.

17) Did authors performed experiments to block proteasome activity? What is the phenotype of BNIP3L/NIX and BNIP3 in FBXL4 KO when proteasome activity is blocked?

RESPONSE: MG132 has diverse effects on cells as it leads to the accumulation of numerous ubiquitylated targets that are no longer degraded. Our experiments showed that prolonged treatment of U2OS cells with MG132 caused cell death, therefore we were limited to short MG132 treatments. To examine the interplay between proteasome inhibition and FBXL4 deficiency, we treated FBXL4-deficient cells with MG132 for a maximum of 6 hours. As expected, we observed an increase in BNIP3 and NIX levels in U2OS cells in response to MG132. In the FBXL4 deficient cells, we found that the proteasome inhibition did not significantly elevate NIX levels and only slightly increased BNIP3 levels in FBXL4-deficient cells, suggesting that CRL2-VHL ligase's role in regulating BNIP3/NIX levels is limited in FBXL4-deficient cells. Nevertheless, we should exercise caution in interpreting these findings, as we could only treat U2OS cells with MG132 for a short time (6 h) before the cells died. Therefore, we include this information in the reviewers' comments, and

not in the final manuscript. However, we will gladly move to the main article if suggested.

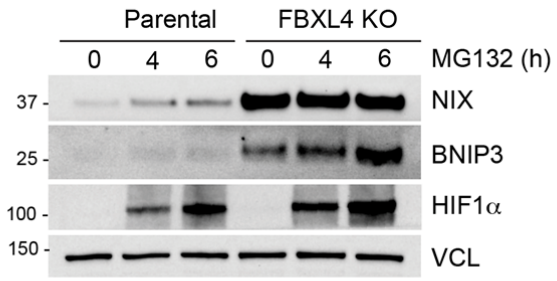

Additional comments:
Recent work by Wilhelm et al, 2022, EMBO journal, have shown that BNIP3L/NIX is needed for pexophagy (similar treatments were used) - it would be great addition to check if FBXL4 influences BNIP3L/NIX- mediated pexophagy in the similar manner.

RESPONSE: Although indeed interesting, we feel that these experiments are beyond the scope of current manuscript.

Dr. Julia Pagan
University of Queensland
Otto Building
QLD 4067
Australia

17th Apr 2023

Re: EMBOJ-2022-112767R
FBXL4 suppresses mitophagy by restricting the accumulation of NIX and BNIP3 mitophagy receptors

Dear Julia,

Thank you for submitting your revised manuscript for our consideration. It has now been assessed once more by two of the original referees (see comments below), and given their satisfaction with the revisions, we shall be happy to accept the study for rapid publication as soon as the following remaining editorial points have been taken care of:

- The text still refers to a "Table 1" (in Material&Methods, p.16) and an "Appendix Table 1" (in figure legends, p.32), neither of which appear to be present. Could you please check if these references should instead both be for "Table EV1", and if so, correct the call-outs, or otherwise upload the respective tables.

- The following funding sources acknowledged in the text appear to still have not been entered into our submission system - can you please enter them/double-check that the info in text and in submission system is congruent:
Brain Foundation Research grant (2020), a Mito Foundation Incubator Grant (2022); National Health and Medical Research Council of Australia grants (APP1140064 and APP1150083 and fellowship APP1156489), AIRC/Marie Curie, American Italian Cancer Foundation (AICF) and NIH/T32CA009161 grant, Mitochondrial Disease Patient Cohort (UK) (G0800674), Medical Research Council International Centre for Genomic Medicine in Neuromuscular Disease (MR/S005021/1), the Medical Research Council (MR/W019027/1), the Lily Foundation, the Pathological Society, the UK NIHR Biomedical Research Centre for Ageing and Age-related disease award to the Newcastle upon Tyne Foundation Hospitals NHS Trust and the UK NHS Highly Specialised Service for Rare Mitochondrial Disorders of Adults and Children, Stafford Fox Foundation Fellowship

- Source data should be provided in separate files for each of the main manuscript figures - this is currently not the case for the numerical source data compiled in a single .XLSX file. Could you please separate them into distinct .XLSX files (1 per each main figure, and 1 combined for all the EV figures)? Also, could you please double-check the source data ZIP file "Figure 1 images", which is the only one I have been unable to open/extract on my computer.

- The current synopsis image is not in the correct format needed for proper display on our website - it should be 550 pixels in width but not more than 600 pixels in height - could you please adapt it slightly to fit these dimensions.

- Finally, please provide suggestions for a short 'blurb' text prefacing and summing up the conceptual aspect of the study in two sentences (max. 250 characters), followed by 3-5 one-sentence 'bullet points' with brief factual statements of key experiments/results of the paper; they will form the basis of an editor-written 'Synopsis' accompanying the online version of the article.

I am returning the manuscript to you for a second round of revision, solely to allow you to incorporate the requested editorial modifications, and upload all modified files. Once we will have received the re-revised files, we should be ready to swiftly proceed with formal acceptance of the manuscript.

With kind regards,

Hartmut

Hartmut Vodermaier, PhD
Senior Editor, The EMBO Journal
h.vodermaier@embojournal.org

Use the link below to submit your revision:

Link Not Available

Referee #1:

The authors have satisfactorily addressed all my concerns and I recommend publication.

Referee #2:

In this revised manuscript, Nguyen-Dien et al. provided additional data and descriptions to clarify most of the points suggested by the referees. In particular, the authors demonstrated that the hyper-stable NIX(delta151-170) and BNIP3(delta181-203) mutants are less efficiently ubiquitylated than the wild-type proteins (Fig. EV2D), and that NIX(delta151-170) accelerates mitophagy in a manner dependent on its LIR, a motif critical for recruiting the mammalian Atg8-family protein LC3 (Fig. EV3F) to mitochondria. Importantly, cells expressing FBXL4(R435*), a mitochondrial DNA depletion syndrome 13-derived mutant, exhibited less efficient NIX ubiquitylation (Fig. EV4F) and enhanced mitophagy that was dependent on NIX and BNIP3 (Fig. 4H). Although the physiological relevance of FBXL4-mediated regulation of NIX/BNIP3-driven mitophagy remains to be elucidated, this study will establish a fundamental concept that mitophagy is tightly controlled both positively and negatively at an appropriate level.

2nd Revision - Editorial Decision **20th Apr 2023**

Dr. Julia Pagan
University of Queensland
Otto Building
QLD 4067
Australia

20th Apr 2023

Re: EMBOJ-2022-112767R1
FBXL4 suppresses mitophagy by restricting the accumulation of NIX and BNIP3 mitophagy receptors

Dear Julia,

Thank you for uploading your final revised manuscript and corrections. I am pleased to inform you that we have now accepted it for publication in The EMBO Journal.

Your article will be processed for publication in The EMBO Journal by EMBO Press and Wiley, who will contact you with further information regarding production/publication procedures and license requirements. You will also be provided with page proofs after copy-editing and typesetting of main manuscript and expanded view figure files.

Should you be planning a Press Release on your article, please get in contact with embojournal@wiley.com as early as possible, in order to coordinate publication and release dates.

Thank you again for this contribution to The EMBO Journal and congratulations on a successful publication! Please consider us again in the future for your most exciting work.

With kind regards,

Hartmut

Hartmut Vodermaier, PhD
Senior Editor, The EMBO Journal
h.vodermaier@embojournal.org

EMBO Press Author Checklist

Corresponding Author Name: Pagan	**USEFUL LINKS FOR COMPLETING THIS FORM**
Journal Submitted to: The EMBO Journal	The EMBO Journal - Author Guidelines
Manuscript Number: 2022-112767R	EMBO Reports - Author Guidelines
	Molecular Systems Biology - Author Guidelines
	EMBO Molecular Medicine - Author Guidelines

Reporting Checklist for Life Science Articles (updated January
This checklist is adapted from Materials Design Analysis Reporting (MDAR) Checklist for Authors. MDAR establishes a minimum set of requirements in transparent reporting in the life sciences (see Statement of Task: 10.31222/osf.io/9sm4x). Please follow the journal's guidelines in preparing your
Please note that a copy of this checklist will be published alongside your article.

Abridged guidelines for figures
1. Data
The data shown in figures should satisfy the following conditions:
- the data were obtained and processed according to the field's best practice and are presented to reflect the results of the experiments in an accurate and unbiased manner.
- ideally, figure panels should include only measurements that are directly comparable to each other and obtained with the same assay.
- plots include clearly labeled error bars for independent experiments and sample sizes. Unless justified, error bars should not be shown for technical
- if n<5, the individual data points from each experiment should be plotted. Any statistical test employed should be justified.
- Source Data should be included to report the data underlying figures according to the guidelines set out in the authorship guidelines on Data

2. Captions
Each figure caption should contain the following information, for each panel where they are relevant:
- a specification of the experimental system investigated (eg cell line, species name).
- the assay(s) and method(s) used to carry out the reported observations and measurements.
- an explicit mention of the biological and chemical entity(ies) that are being measured.
- an explicit mention of the biological and chemical entity(ies) that are altered/varied/perturbed in a controlled manner.
- the exact sample size (n) for each experimental group/condition, given as a number, not a range;
- a description of the sample collection allowing the reader to understand whether the samples represent technical or biological replicates (including how many animals, litters, cultures, etc.).
- a statement of how many times the experiment shown was independently replicated in the laboratory.
- definitions of statistical methods and measures:
  - common tests, such as t-test (please specify whether paired vs. unpaired), simple χ2 tests, Wilcoxon and Mann-Whitney tests, can be unambiguously identified by name only, but more complex techniques should be described in the methods section;
  - are tests one-sided or two-sided?
  - are there adjustments for multiple comparisons?
  - exact statistical test results, e.g., P values = x but not P values < x;
  - definition of 'center values' as median or average;
  - definition of error bars as s.d. or s.e.m.

Please complete ALL of the questions below.
Select "Not Applicable" only when the requested information is not relevant for your study.

Materials

Newly Created Materials	Information included in the manuscript?	In which section is the information available? (Reagents and Tools Table, Materials and Methods, Figures, Data Availability Section)
New materials and reagents need to be available; do any restrictions apply?	Not Applicable	No restrictions apply

Antibodies	Information included in the manuscript?	In which section is the information available? (Reagents and Tools Table, Materials and Methods, Figures, Data Availability Section)
For **antibodies** provide the following information:<br>- Commercial antibodies: RRID (if possible) or supplier name, catalogue number and or/clone number<br>- Non-commercial: RRID or citation	Yes	Materials and Methods

DNA and RNA sequences	Information included in the manuscript?	In which section is the information available? (Reagents and Tools Table, Materials and Methods, Figures, Data Availability Section)
**Short novel DNA or RNA including primers, probes**: provide the sequences.	Yes	Materials and Methods

Cell materials	Information included in the manuscript?	In which section is the information available? (Reagents and Tools Table, Materials and Methods, Figures, Data Availability Section)
**Cell lines:** Provide species information, strain. Provide accession number in repository **OR** supplier name, catalog number, clone number, and/**OR** RRID.	Yes	Materials and Methods
**Primary cultures:** Provide species, strain, sex of origin, genetic modification status.	Yes	Materials and Methods
Report if the cell lines were recently **authenticated** (e.g., by STR profiling) and tested for mycoplasma contamination.	Yes	Materials and Methods

Experimental animals	Information included in the manuscript?	In which section is the information available? (Reagents and Tools Table, Materials and Methods, Figures, Data Availability Section)
**Laboratory animals or Model organisms:** Provide species, strain, sex, age, genetic modification status. Provide accession number in repository **OR** supplier name, catalog number, clone number, **OR** RRID.	Not Applicable	
**Animal observed in or captured from the field:** Provide species, sex, and age where possible.	Not Applicable	
Please detail **housing and husbandry conditions**.	Not Applicable	

Plants and microbes	Information included in the manuscript?	In which section is the information available? (Reagents and Tools Table, Materials and Methods, Figures, Data Availability Section)
**Plants:** provide species and strain, ecotype and cultivar where relevant, unique accession number if available, and source (including location for collected wild specimens).	Not Applicable	
**Microbes:** provide species and strain, unique accession number if available, and source.	Not Applicable	

Human research participants	Information included in the manuscript?	In which section is the information available? (Reagents and Tools Table, Materials and Methods, Figures, Data Availability Section)
If collected and within the bounds of privacy constraints report on age, sex and gender or ethnicity for all study participants.	Not Applicable	

Core facilities	Information included in the manuscript?	In which section is the information available? (Reagents and Tools Table, Materials and Methods, Figures, Data Availability Section)
If your work benefited from core facilities, was their service mentioned in the acknowledgments section?	Yes	Methods and Materials

Design

Study protocol	Information included in the manuscript?	In which section is the information available? (Reagents and Tools Table, Materials and Methods, Figures, Data Availability Section)
If study protocol has been **pre-registered, provide DOI in the manuscript**. For clinical trials, provide the trial registration number **OR** cite DOI.	Not Applicable	
Report the **clinical trial registration number** (at ClinicalTrials.gov or equivalent), where applicable.	Not Applicable	

Laboratory protocol	Information included in the manuscript?	In which section is the information available? (Reagents and Tools Table, Materials and Methods, Figures, Data Availability Section)
Provide DOI OR other citation details if **external detailed step-by-step protocols** are available.	Not Applicable	

Experimental study design and statistics	Information included in the manuscript?	In which section is the information available? (Reagents and Tools Table, Materials and Methods, Figures, Data Availability Section)
Include a statement about **sample size** estimate even if no statistical methods were used.	Yes	Materials and Methods
Were any steps taken to minimize the effects of subjective bias when allocating animals/samples to treatment (e.g. **randomization procedure**)? If yes, have they been described?	Yes	Materials and Methods
Include a statement about **blinding** even if no blinding was done.	Yes	Materials and Methods
Describe **inclusion/exclusion criteria** if samples or animals were excluded from the analysis. Were the criteria pre-established?<br><br>If sample or data points were omitted from analysis, report if this was due to attrition or intentional exclusion and provide justification.	Yes	Materials and Methods
For every figure, are **statistical tests** justified as appropriate? Do the data meet the assumptions of the tests (e.g., normal distribution)? Describe any methods used to assess it. Is there an estimate of variation within each group of data? Is the variance similar between the groups that are being statistically compared?	Yes	Materials and Methods

Sample definition and in-laboratory replication	Information included in the manuscript?	In which section is the information available? (Reagents and Tools Table, Materials and Methods, Figures, Data Availability Section)
In the figure legends: state number of times the experiment was **replicated** in laboratory.	Not Applicable	
In the figure legends: define whether data describe **technical or biological replicates**.	Not Applicable	

Ethics

Ethics	Information included in the manuscript?	In which section is the information available? (Reagents and Tools Table, Materials and Methods, Figures, Data Availability Section)
Studies involving **human participants**: State details of **authority granting ethics approval** (IRB or equivalent committee(s), provide reference number for approval.	Not Applicable	
Studies involving **human participants**: Include a statement confirming that **informed consent** was obtained from all subjects and that the experiments conformed to the principles set out in the WMA Declaration of Helsinki and the Department of Health and Human Services Belmont Report.	Not Applicable	
Studies involving **human participants:** For publication of **patient photos**, include a statement confirming that consent to publish was obtained.	Not Applicable	
Studies involving experimental **animals**: State details of **authority granting ethics approval** (IRB or equivalent committee(s), provide reference number for approval. Include a statement of compliance with ethical regulations.	Not Applicable	
Studies involving **specimen and field samples:** State if relevant **permits** obtained, provide details of authority approving study; if none were required, explain why.	Not Applicable	

Dual Use Research of Concern (DURC)	Information included in the manuscript?	In which section is the information available? (Reagents and Tools Table, Materials and Methods, Figures, Data Availability Section)
Could your study fall under dual use research restrictions? Please check biosecurity documents and list of **select agents and toxins** (CDC): https://www.selectagents.gov/sat/list.htm	Not Applicable	
If you used a select agent, is the security level of the lab appropriate and reported in the manuscript?	Not Applicable	
If a study is subject to dual use research of concern regulations, is the name of the **authority granting approval and reference number** for the regulatory approval provided in the manuscript?	Not Applicable	

Reporting

The MDAR framework recommends adoption of discipline-specific guidelines, established and endorsed through community initiatives. Journals have their own policy about requiring specific guidelines and recommendations to complement MDAR.

Adherence to community standards	Information included in the manuscript?	In which section is the information available? (Reagents and Tools Table, Materials and Methods, Figures, Data Availability Section)
State if relevant guidelines or checklists (e.g., **ICMJE, MIBBI, ARRIVE, PRISMA**) have been followed or provided.	Not Applicable	
For **tumor marker prognostic studies,** we recommend that you follow the **REMARK** reporting guidelines (see link list at top right). See author guidelines, under 'Reporting Guidelines'. Please confirm you have followed these guidelines.	Not Applicable	
For **phase II and III randomized controlled trials**, please refer to the **CONSORT** flow diagram (see link list at top right) and submit the CONSORT checklist (see link list at top right) with your submission. See author guidelines, under 'Reporting Guidelines'. Please confirm you have submitted this list.	Not Applicable	

Data Availability

Data availability	Information included in the manuscript?	In which section is the information available? (Reagents and Tools Table, Materials and Methods, Figures, Data Availability Section)
Have **primary datasets** been deposited according to the journal's guidelines (see 'Data Deposition' section) and the respective accession numbers provided in the Data Availability Section?	Yes	
Were **human clinical and genomic datasets** deposited in a public access-controlled repository in accordance to ethical obligations to the patients and to the applicable consent agreement?	Not Applicable	
Are **computational models** that are central and integral to a study available without restrictions in a machine-readable form? Were the relevant accession numbers or links provided?	Not Applicable	
If publicly available data were reused, provide the respective **data citations in the reference list**.	Not Applicable	
