# Supplementary material for: FBXL4 suppresses mitophagy by restricting the accumulation of NIX and BNIP3 mitophagy receptors
Source: EMBO J. 2023 May 10;42(13):e112767. doi: 10.15252/embj.2022112767 (PMC10308361; doi:10.15252/embj.2022112767)
Supplement: Supplementary file 5 — Source Data for Figure 1 [file EMBJ-42-e112767-s001.zip › FIGURE_1.zip!/FIGURE 1 /1G /Annotations_Fig 1G.pdf]

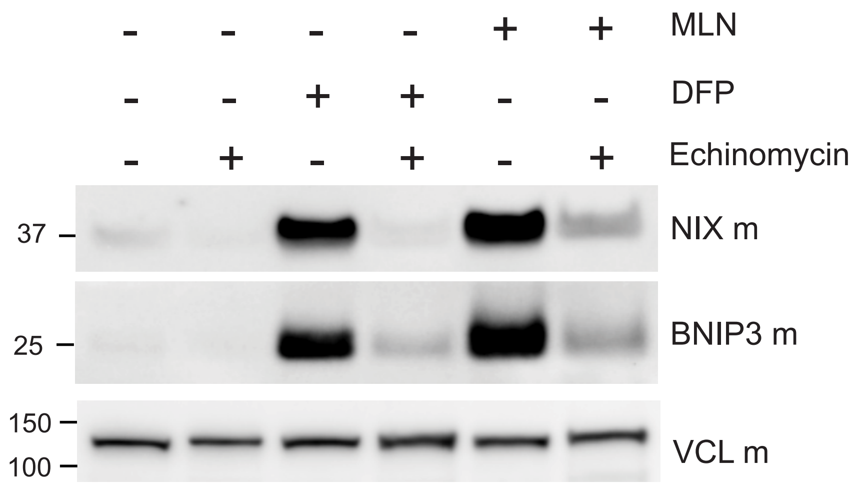

- - - - + + MLN
- - + + - - DFP
- + - + - + Echinomycin
37
NIX m
25
BNIP3 m
150
100
VCL m

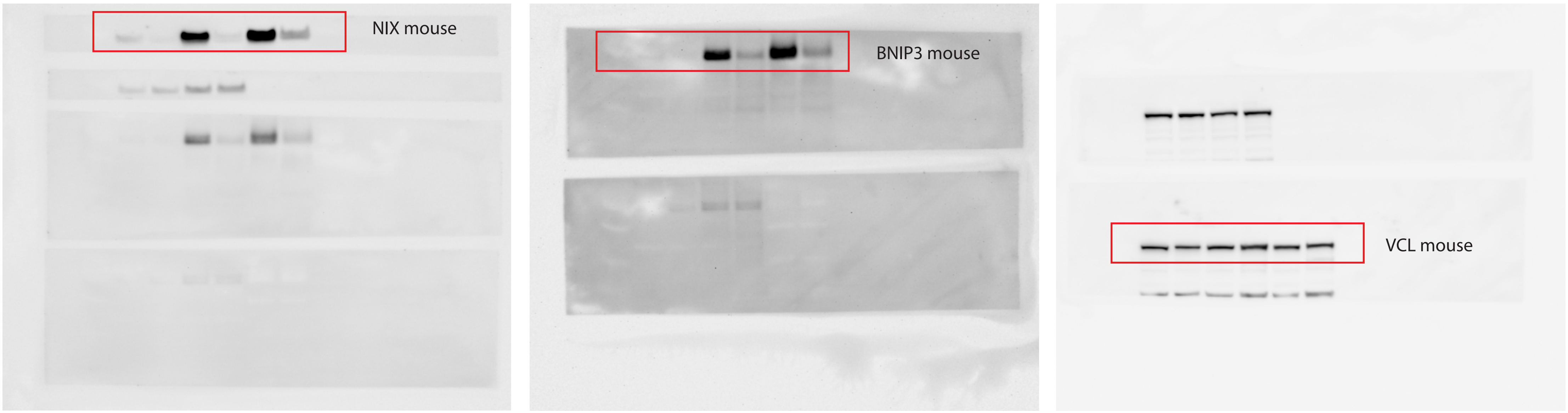

NIX mouse
BNIP3 mouse
VCL mouse

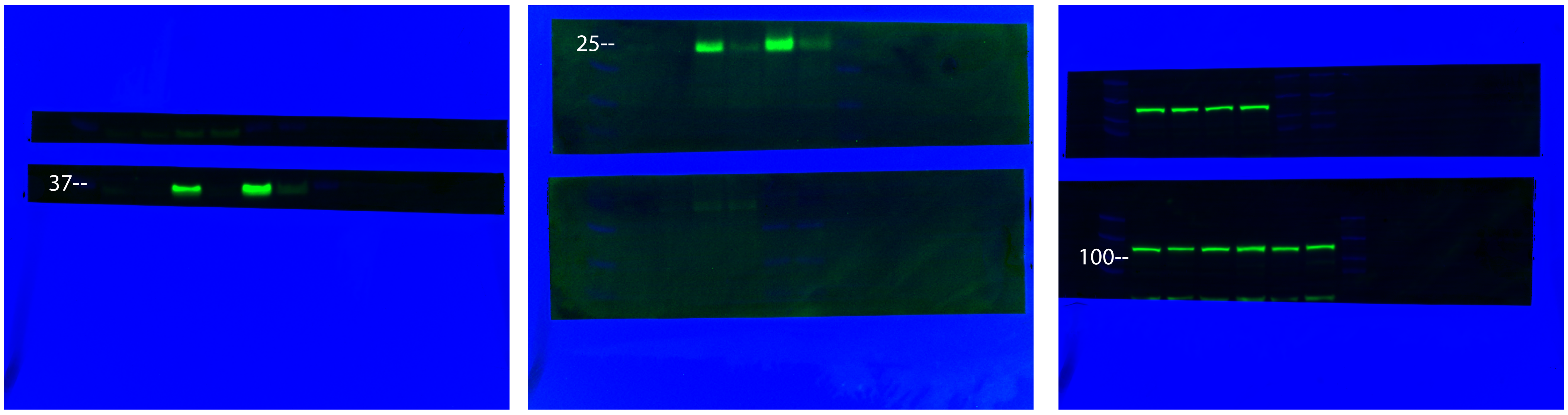

37--
25--
100--
