# Supplementary material for: FBXL4 suppresses mitophagy by restricting the accumulation of NIX and BNIP3 mitophagy receptors
Source: EMBO J. 2023 May 10;42(13):e112767. doi: 10.15252/embj.2022112767 (PMC10308361; doi:10.15252/embj.2022112767)
Supplement: Supplementary file 5 — Source Data for Figure 1 [file EMBJ-42-e112767-s001.zip › FIGURE_1.zip!/FIGURE 1 /1C /Annotations_Fig 1C.pdf]

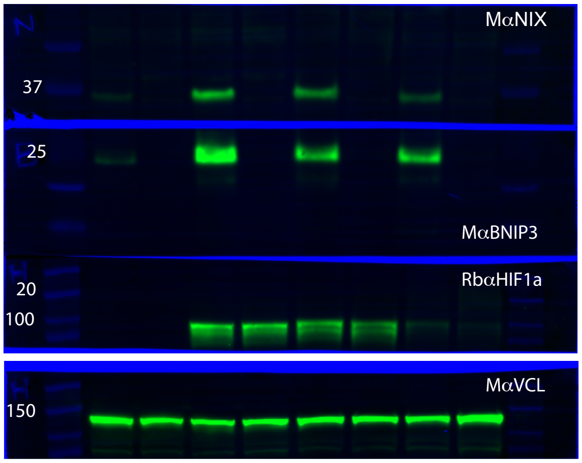
MαNIX
37
25
MαBNIP3
RbαHIF1a
20
100
MαVCL
150

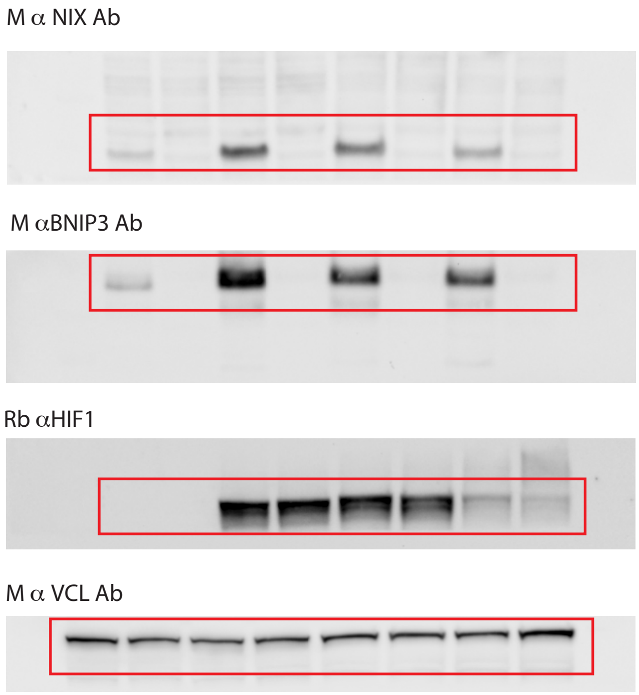
M α NIX Ab
M αBNIP3 Ab
Rb αHIF1
M α VCL Ab
