# Supplementary material for: FBXL4 suppresses mitophagy by restricting the accumulation of NIX and BNIP3 mitophagy receptors
Source: EMBO J. 2023 May 10;42(13):e112767. doi: 10.15252/embj.2022112767 (PMC10308361; doi:10.15252/embj.2022112767)
Supplement: Supplementary file 6 — Source Data for Figure 2 [file EMBJ-42-e112767-s010.zip › FIGURE_2.zip!/FIGURE 2/2A /Annotations_Fig-2A.pdf]

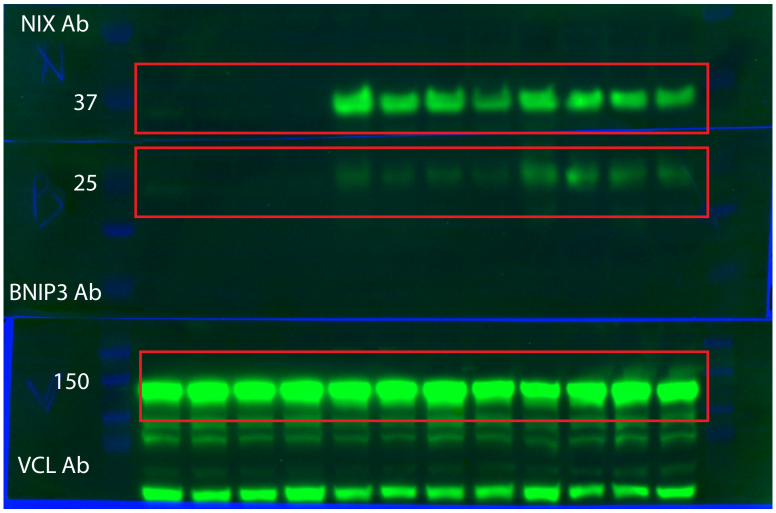

NIX Ab
37
25
BNIP3 Ab
150
VCL Ab

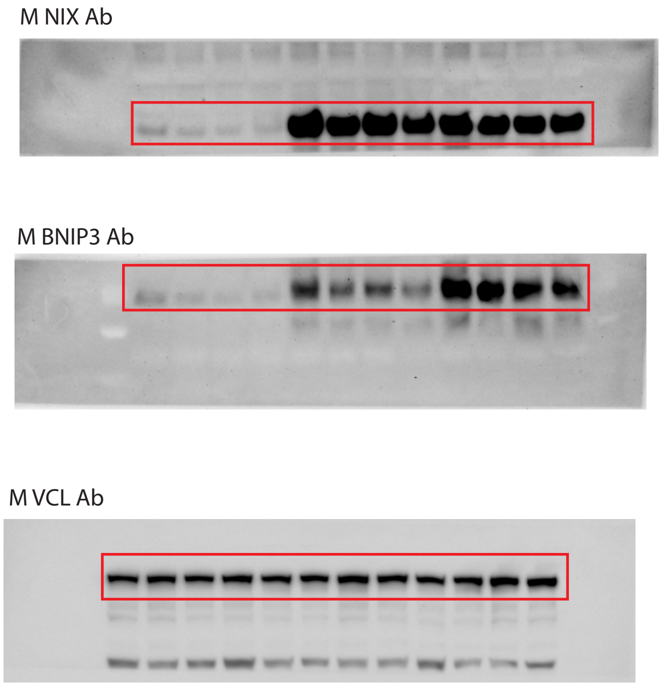

M NIX Ab
M BNIP3 Ab
M VCL Ab

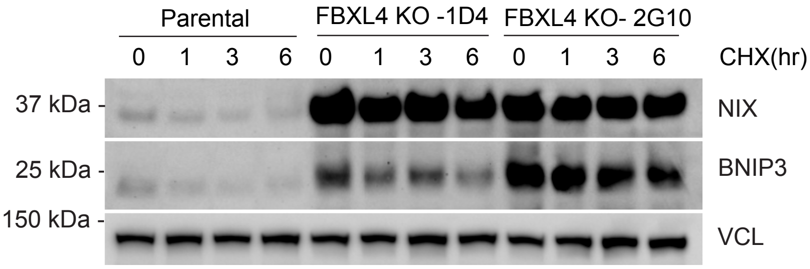

Parental
FBXL4 KO -1D4
FBXL4 KO- 2G10
0 1 3 6 0 1 3 6 0 1 3 6
CHX(hr)
37 kDa -
25 kDa -
150 kDa -
NIX
BNIP3
VCL
