# Supplementary material for: FBXL4 suppresses mitophagy by restricting the accumulation of NIX and BNIP3 mitophagy receptors
Source: EMBO J. 2023 May 10;42(13):e112767. doi: 10.15252/embj.2022112767 (PMC10308361; doi:10.15252/embj.2022112767)
Supplement: Supplementary file 6 — Source Data for Figure 2 [file EMBJ-42-e112767-s010.zip › FIGURE_2.zip!/FIGURE 2/2G /Annotated 2E Ubiquitylation assay.pdf]

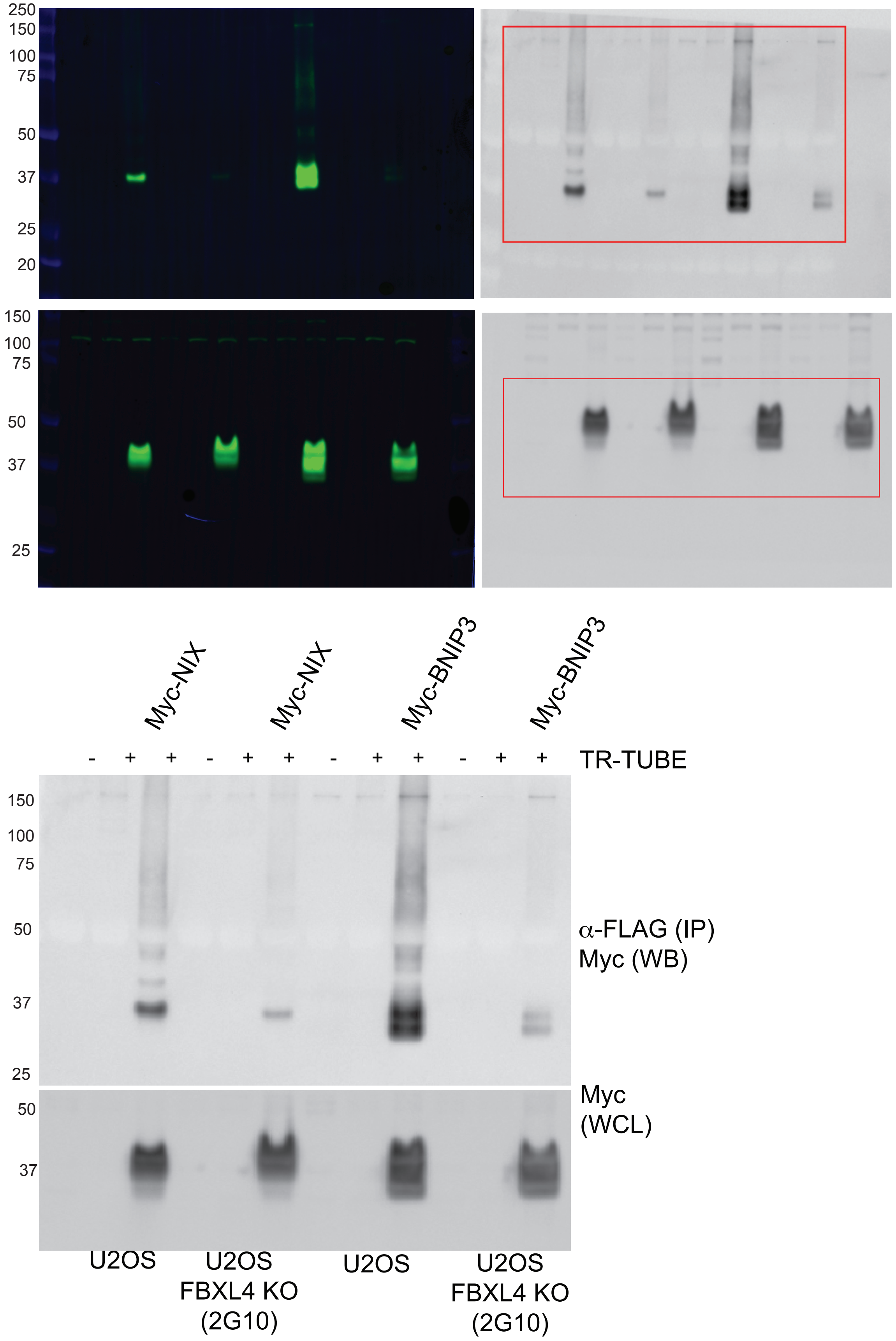
250
150
100
75
50
37
25
20
150
100
75
50
37
25
Myc-NIX
Myc-NIX
Myc-BNIP3
Myc-BNIP3
- + + - + + - + + - + +
TR-TUBE
150
100
75
50
37
25
α-FLAG (IP)
Myc (WB)
50
37
Myc
(WCL)
U2OS
U2OS
FBXL4 KO
(2G10)
U2OS
U2OS
FBXL4 KO
(2G10)
