# Supplementary material for: FBXL4 suppresses mitophagy by restricting the accumulation of NIX and BNIP3 mitophagy receptors
Source: EMBO J. 2023 May 10;42(13):e112767. doi: 10.15252/embj.2022112767 (PMC10308361; doi:10.15252/embj.2022112767)
Supplement: Supplementary file 6 — Source Data for Figure 2 [file EMBJ-42-e112767-s010.zip › FIGURE_2.zip!/FIGURE 2/2C/Annotations_Fig EV2-A.pdf]

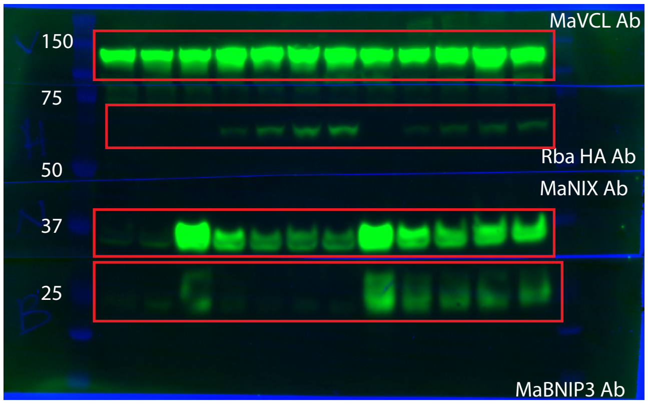

MaVCL Ab
150
75
Rba HA Ab
50
MaNIX Ab
37
25
MaBNIP3 Ab

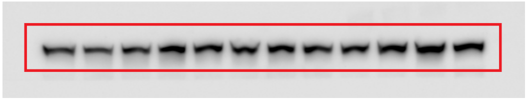

M a VCL Ab

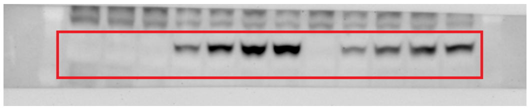

Rb a HA Ab

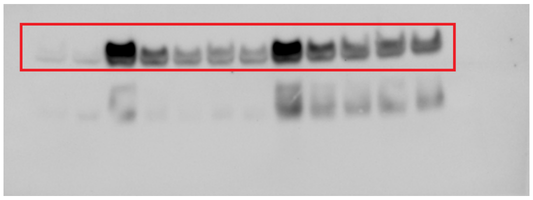

M a NIX Ab

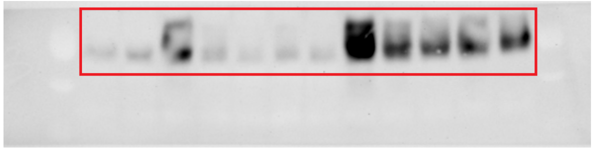

M a BNIP3 Ab

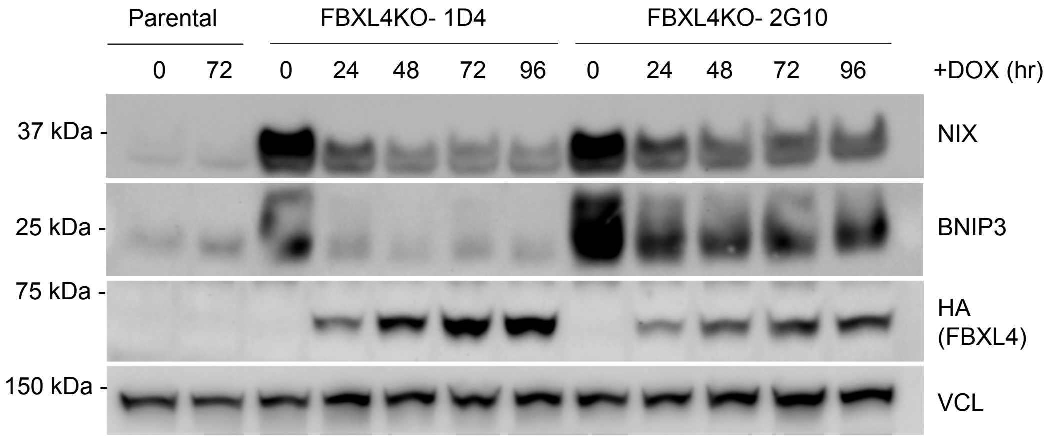

Parental
FBXL4KO- 1D4
FBXL4KO- 2G10
0 72 0 24 48 72 96 0 24 48 72 96
+DOX (hr)
37 kDa -
NIX
25 kDa -
BNIP3
75 kDa -
HA (FBXL4)
150 kDa -
VCL
