# Supplementary material for: FBXL4 suppresses mitophagy by restricting the accumulation of NIX and BNIP3 mitophagy receptors
Source: EMBO J. 2023 May 10;42(13):e112767. doi: 10.15252/embj.2022112767 (PMC10308361; doi:10.15252/embj.2022112767)

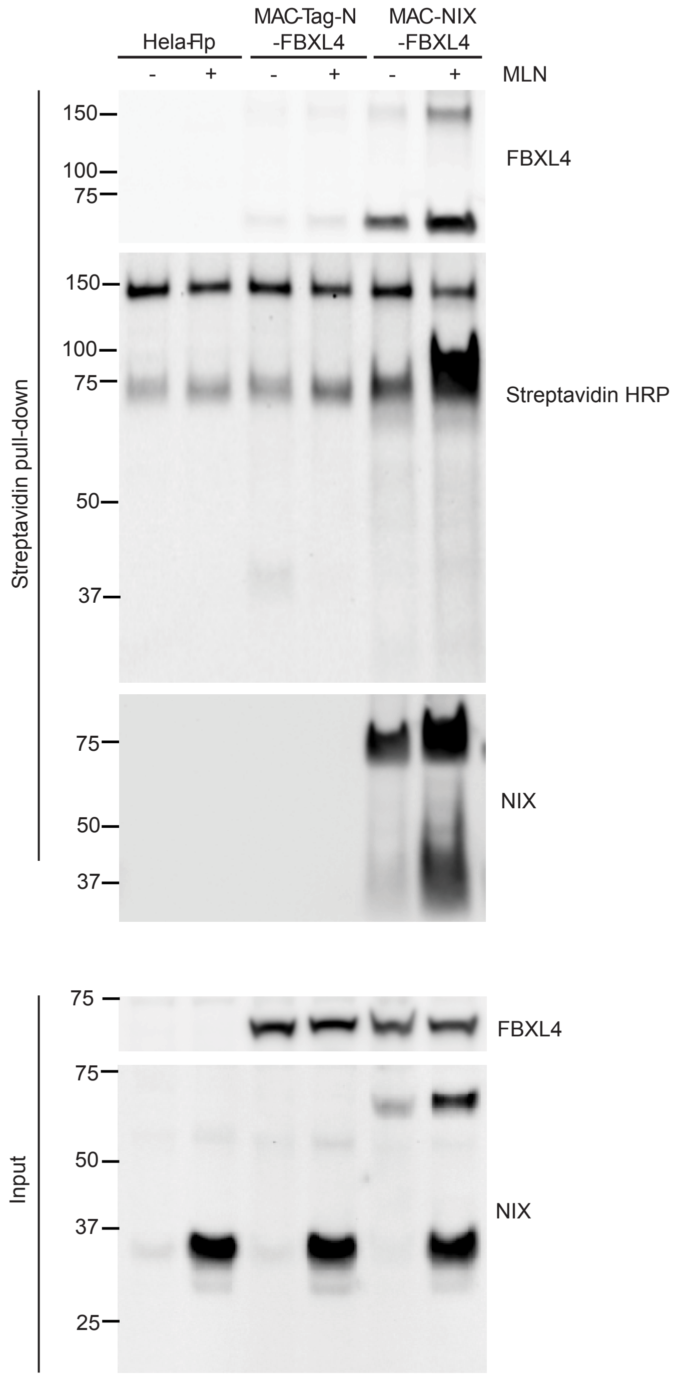

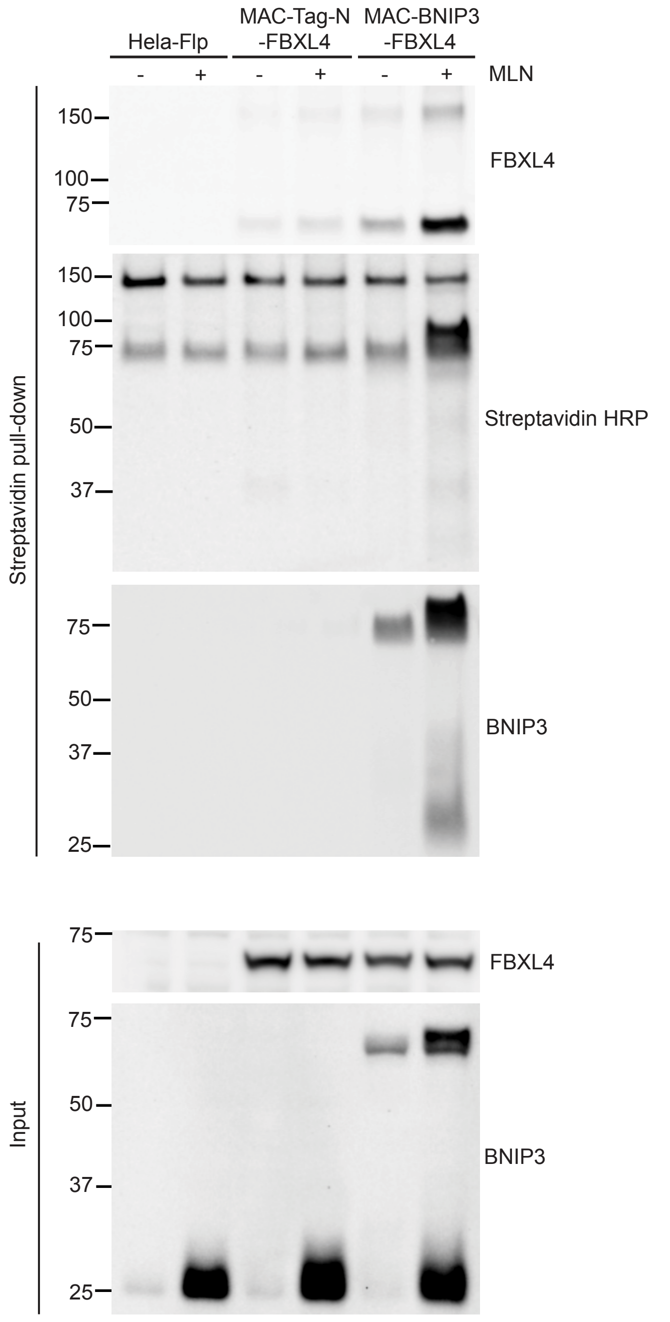

MAC-BNIP3: Input, FBXL4 antibody

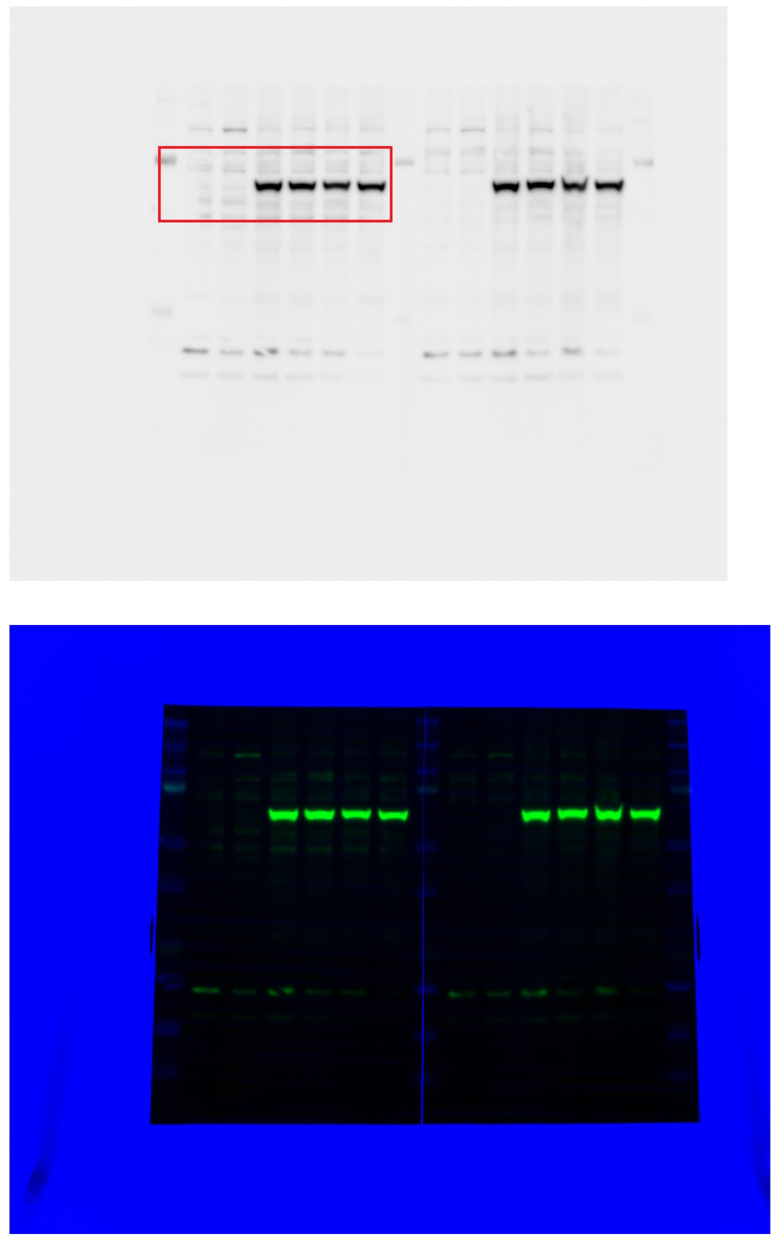

MAC-BNIP3: Streptavidin pull-down, Streptavidin HRP antibody

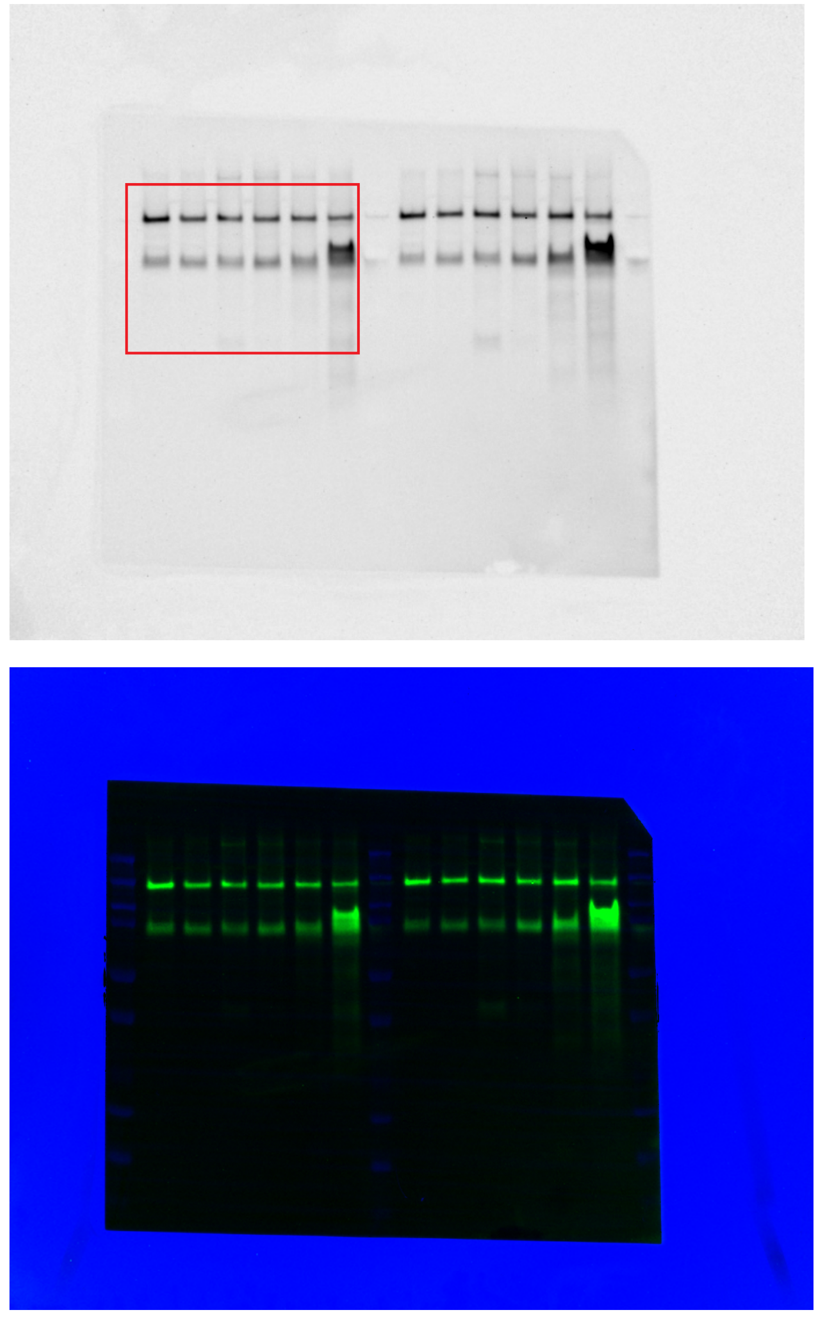

MAC-BNIP3: Streptavidin pull-down, FBXL4 antibody

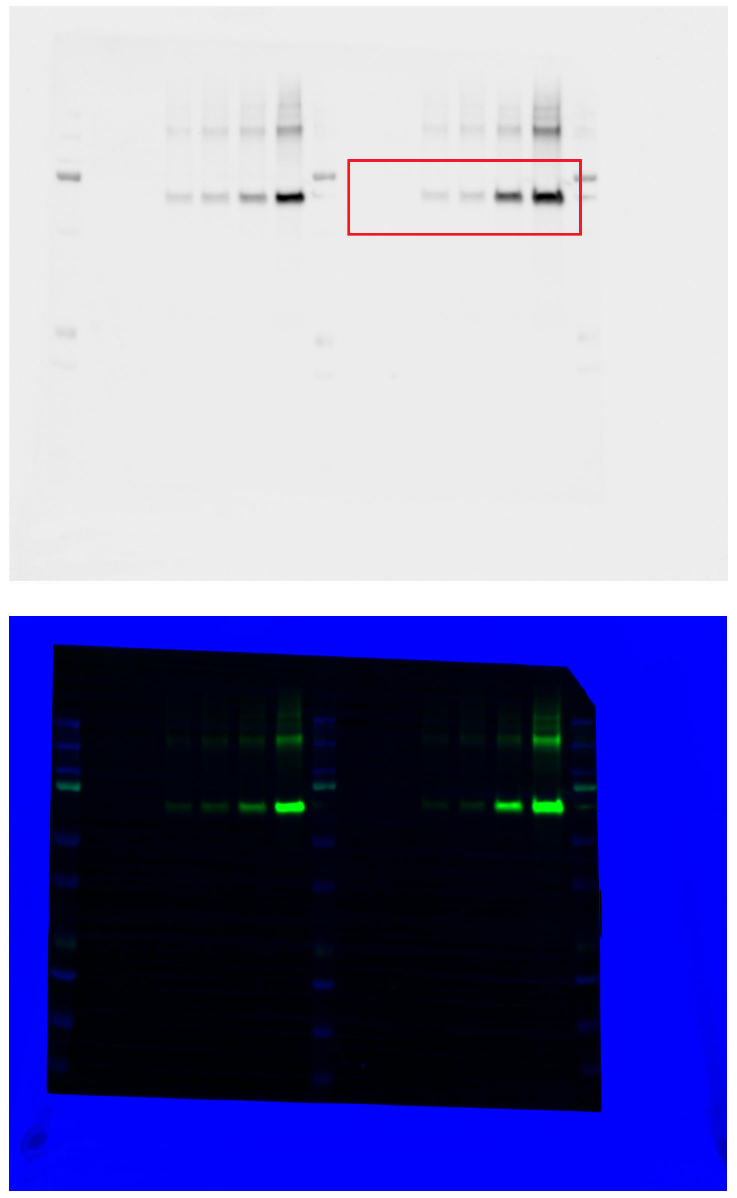

MAC-BNIP3: Streptavidin pull-down, BNIP3 antibody

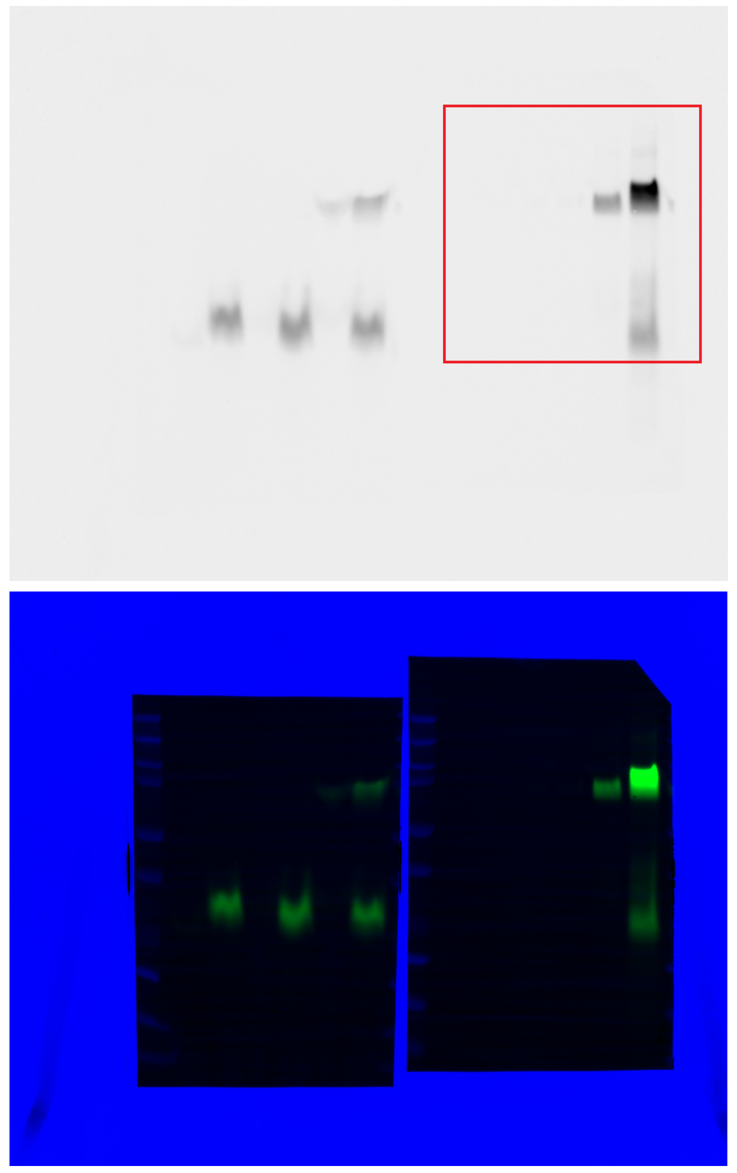

MAC-BNIP3: Input, BNIP3 antibody

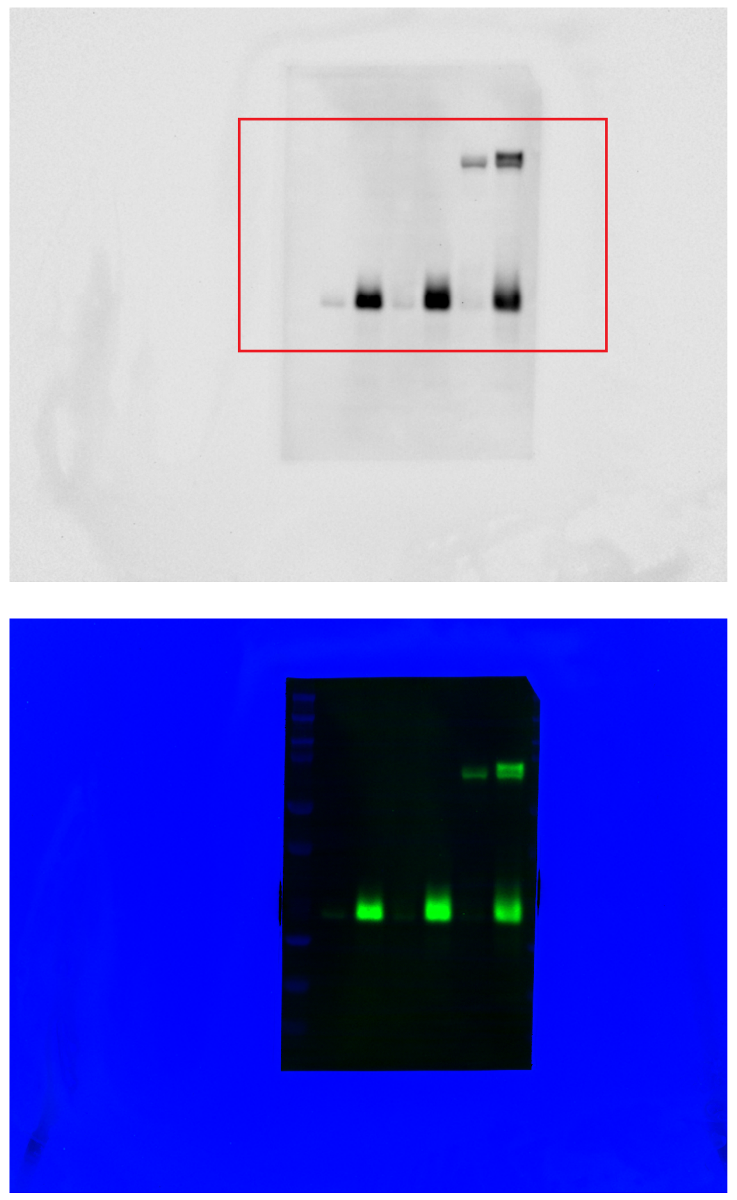

MAC-NIX: Input, FBXL4 antibody

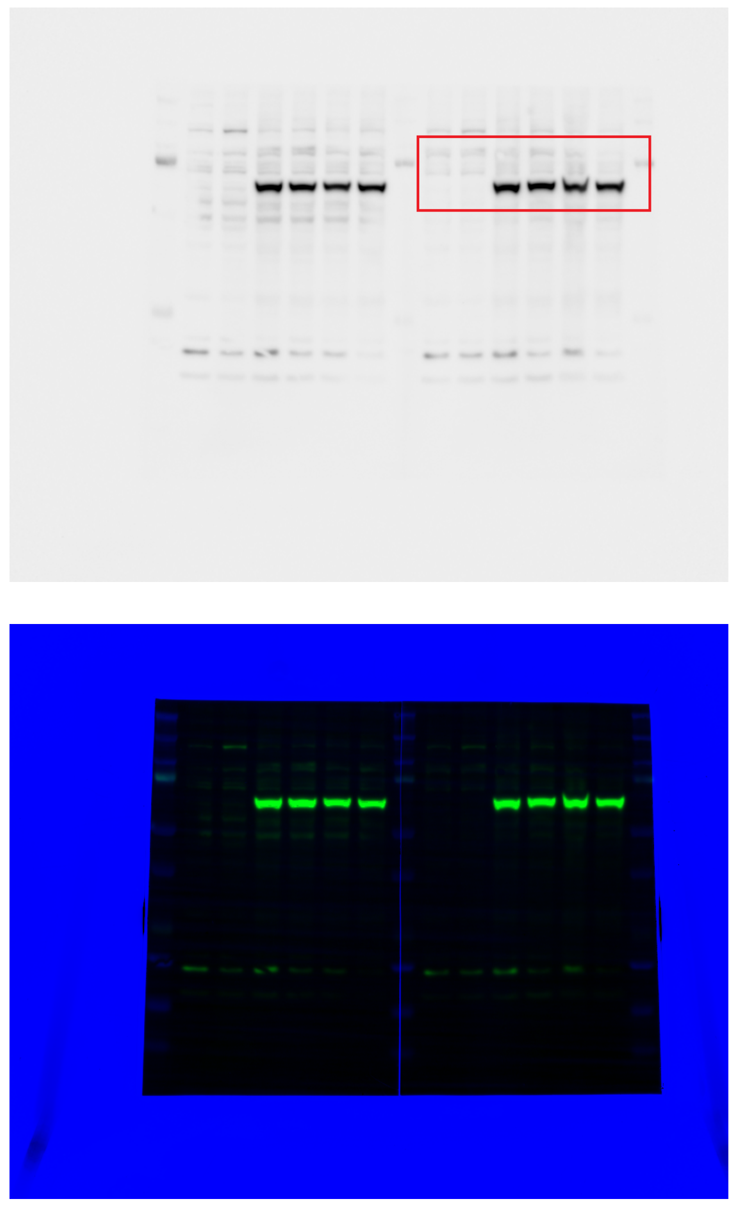

MAC-NIX: Input, NIX antibody

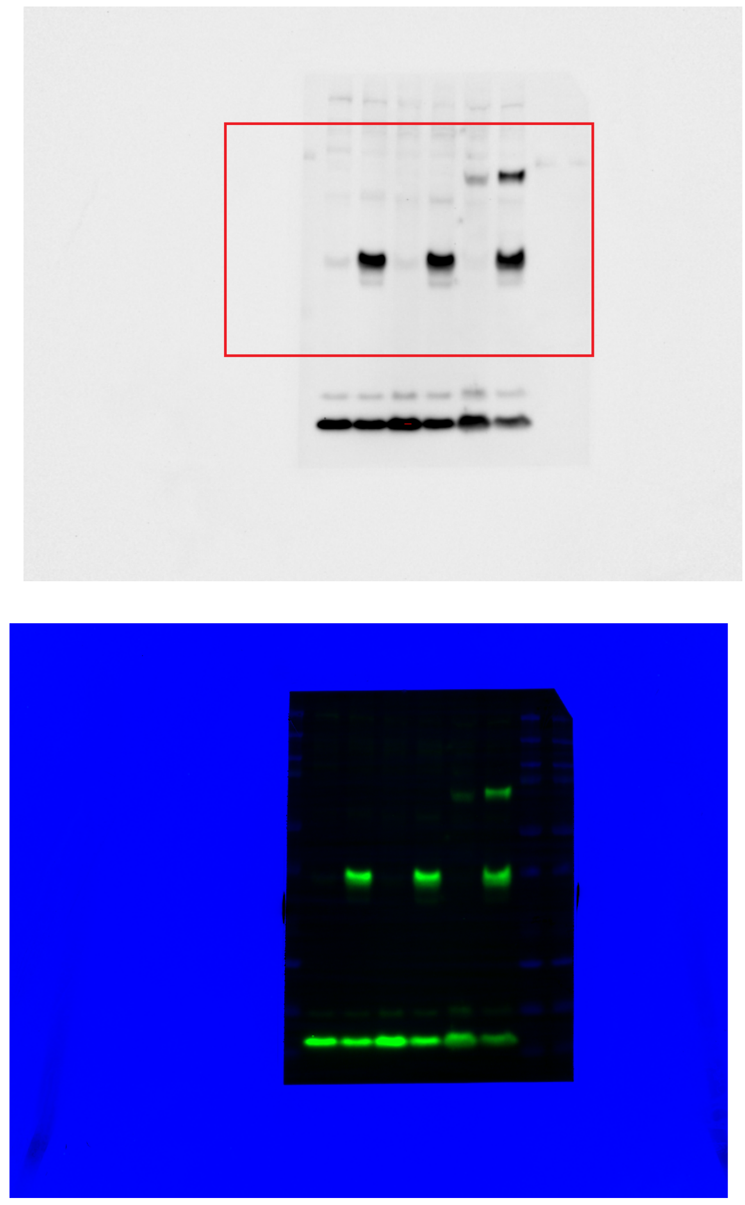

MAC-NIX: Streptavidin pull-down, FBXL4 antibody

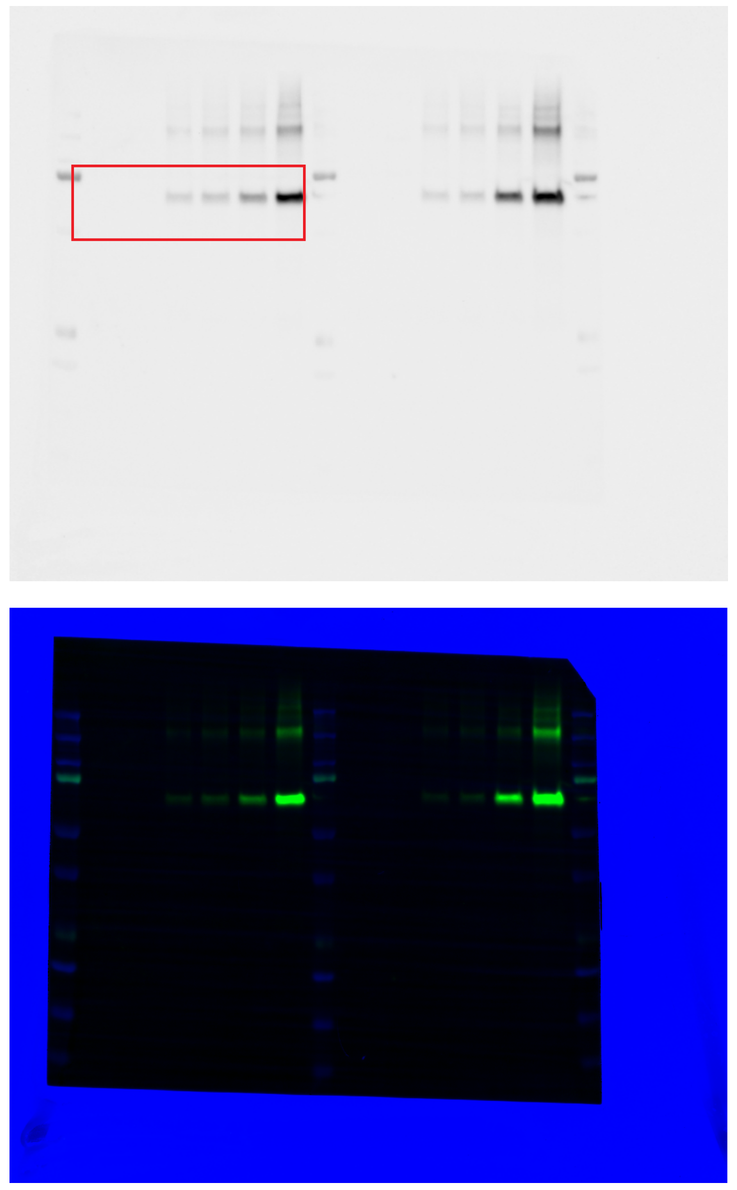

MAC-NIX: Streptavidin pull-down, NIX antibody

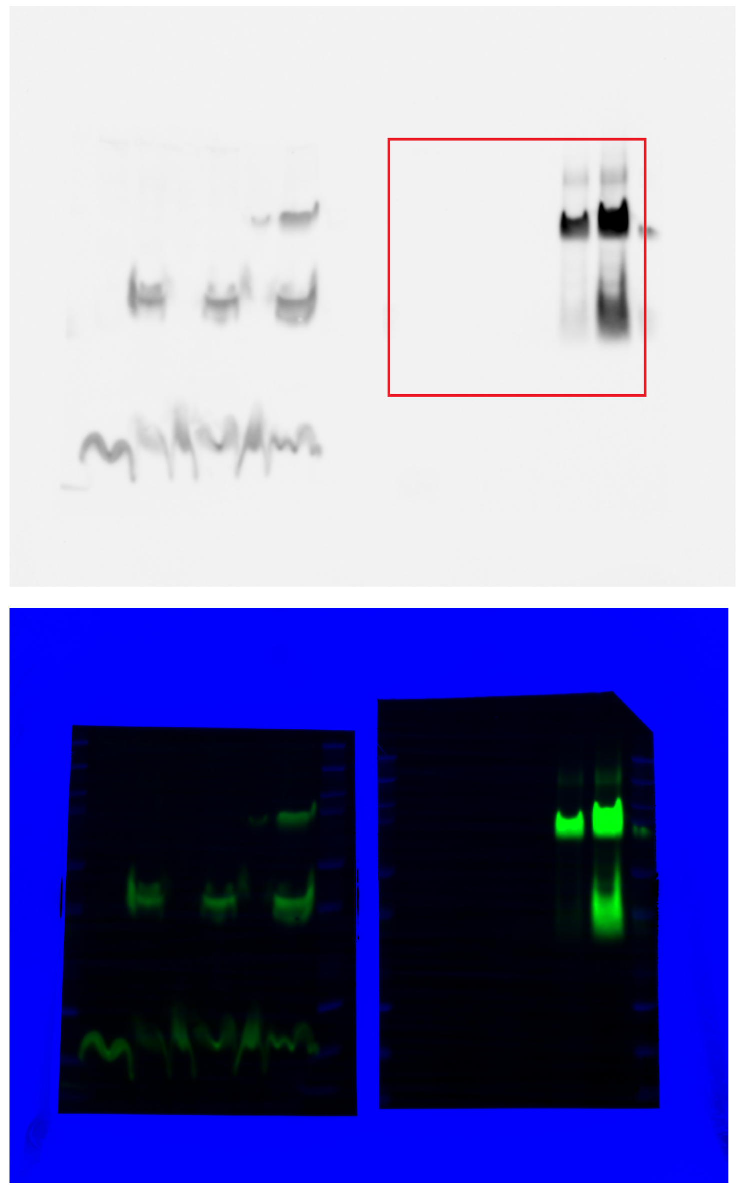

MAC-NIX: Streptavidin pull-down, Streptavidin HRP antibody

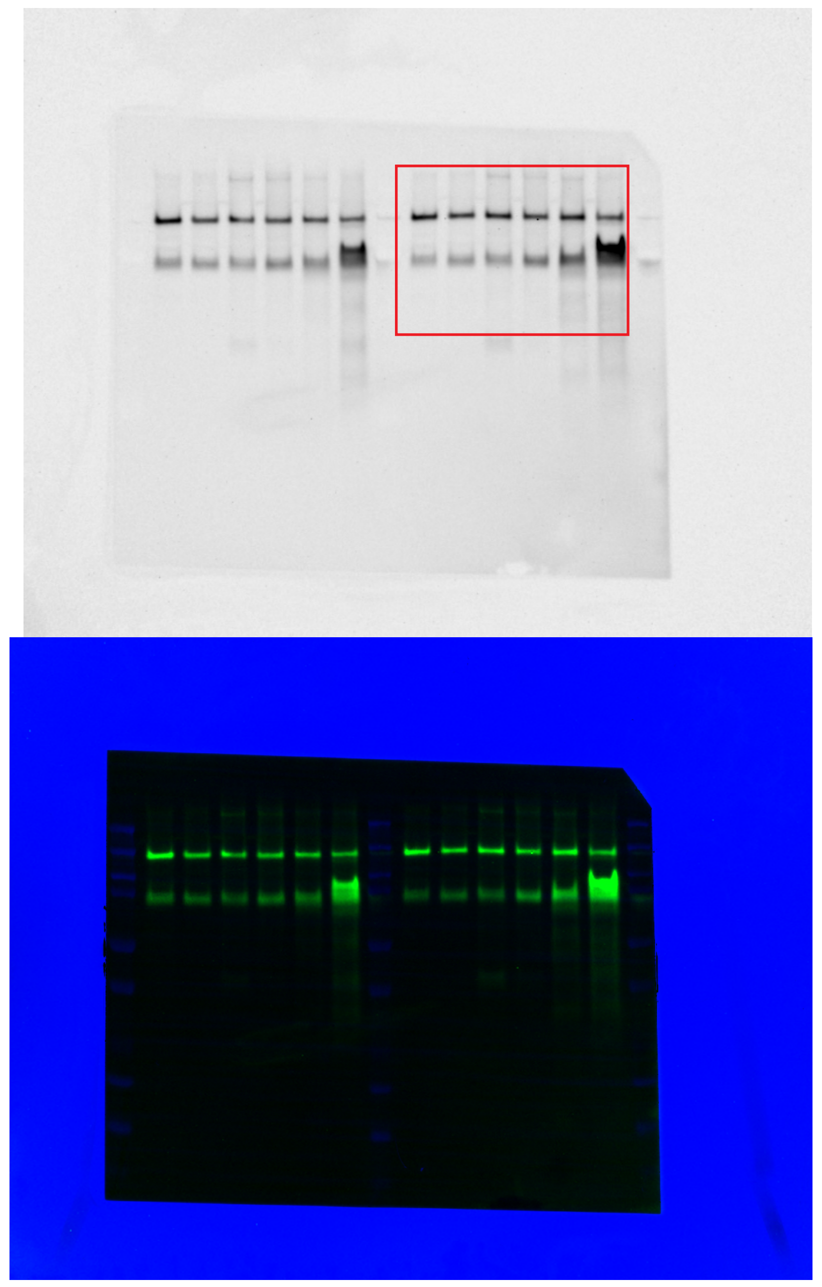

Supplement: Supplementary file 6 — Source Data for Figure 2 [file EMBJ-42-e112767-s010.zip › FIGURE_2.zip!/FIGURE 2/2F/Annotated 2F.pdf]
