# Supplementary material for: FBXL4 suppresses mitophagy by restricting the accumulation of NIX and BNIP3 mitophagy receptors
Source: EMBO J. 2023 May 10;42(13):e112767. doi: 10.15252/embj.2022112767 (PMC10308361; doi:10.15252/embj.2022112767)

# Figure 2D

VCL mouse 1:1000

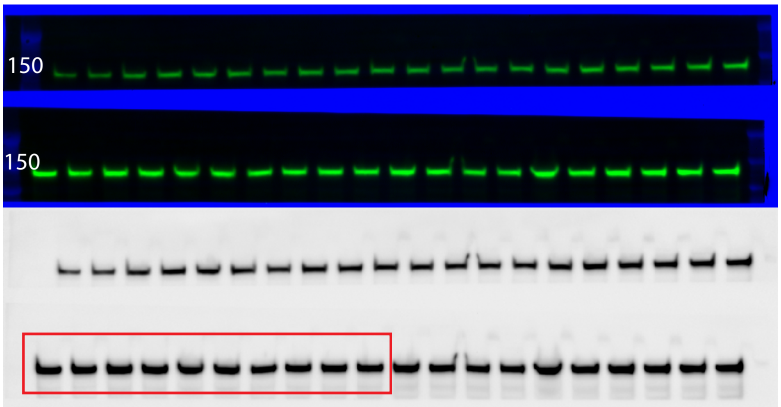


p27 mouse 1:1000

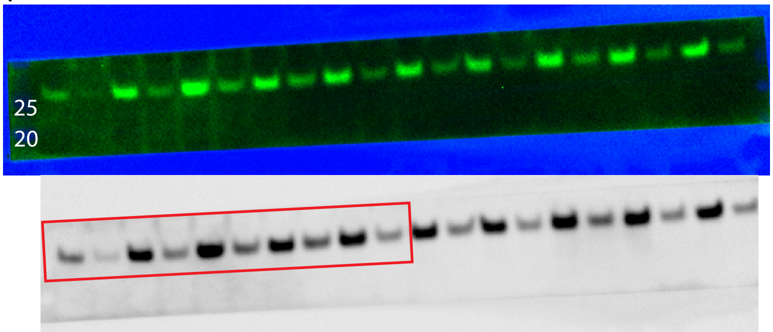


BNIP3 Mouse 1:500

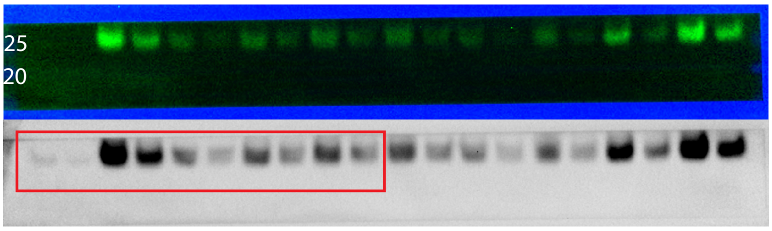


HA Rb 1:1000

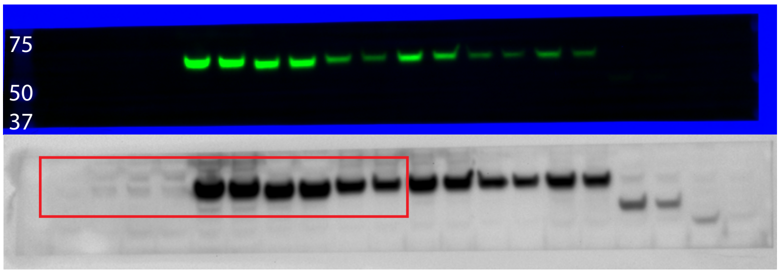


NIX mouse 1:1000

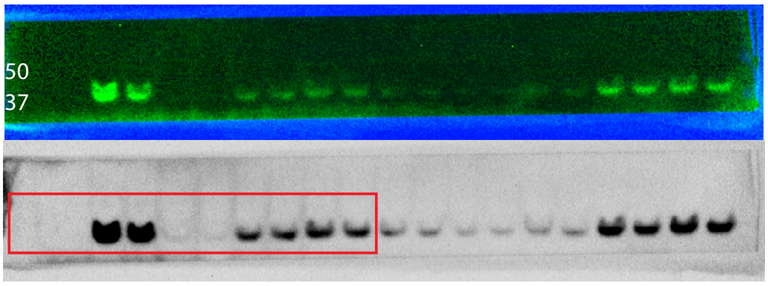


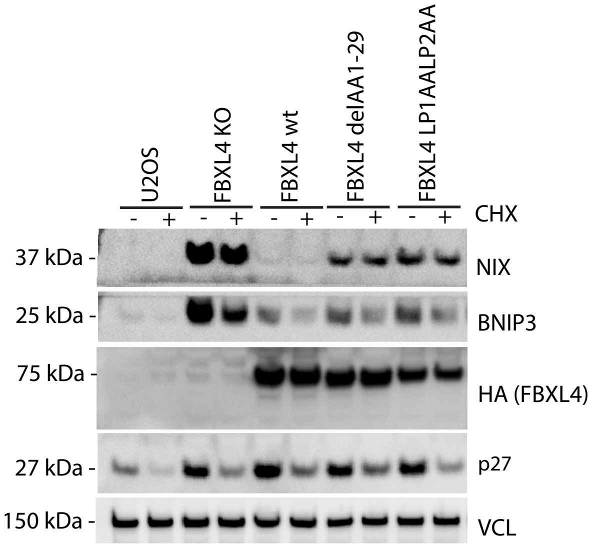

Supplement: Supplementary file 6 — Source Data for Figure 2 [file EMBJ-42-e112767-s010.zip › FIGURE_2.zip!/FIGURE 2/2D /Annotations - Fig 2D edit.pdf]
