# Supplementary material for: FBXL4 suppresses mitophagy by restricting the accumulation of NIX and BNIP3 mitophagy receptors
Source: EMBO J. 2023 May 10;42(13):e112767. doi: 10.15252/embj.2022112767 (PMC10308361; doi:10.15252/embj.2022112767)
Supplement: Supplementary file 6 — Source Data for Figure 2 [file EMBJ-42-e112767-s010.zip › Figure_2_Images.zip!/Figure 2 Images /2B BNIP3 NIX FBXL4 2G10 KO/Annotations_Fig 2B.pdf]

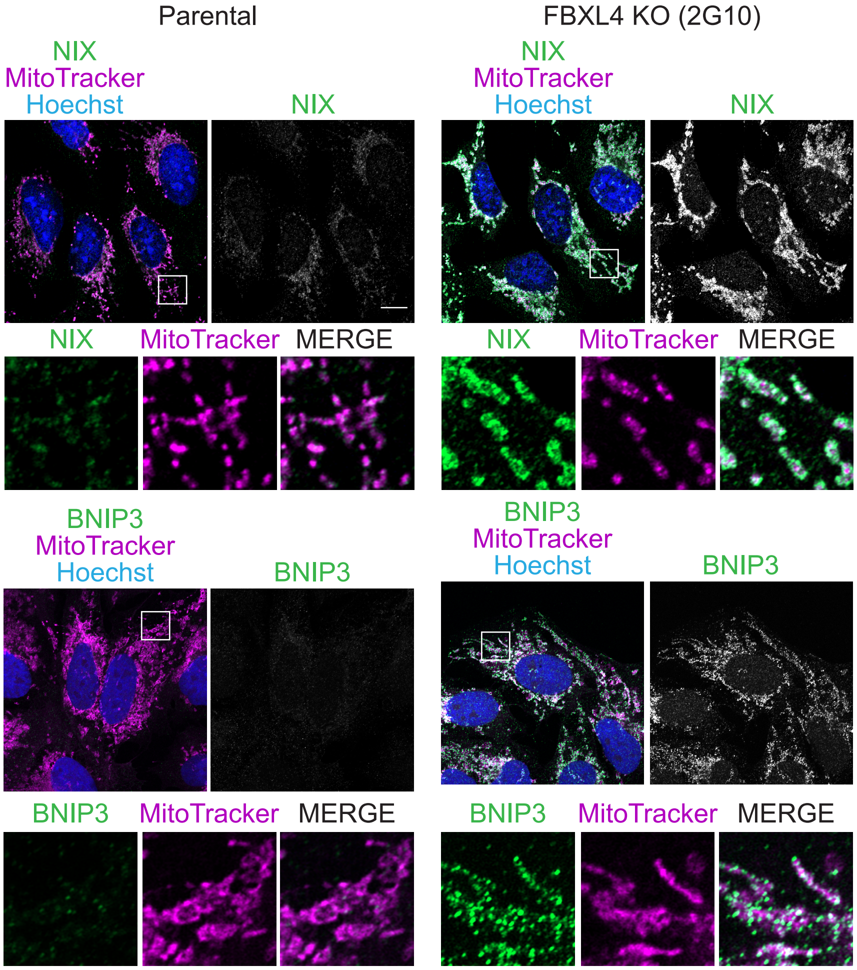

Scale bar 10 micron
inset 10 micron
