# Supplementary material for: FBXL4 suppresses mitophagy by restricting the accumulation of NIX and BNIP3 mitophagy receptors
Source: EMBO J. 2023 May 10;42(13):e112767. doi: 10.15252/embj.2022112767 (PMC10308361; doi:10.15252/embj.2022112767)
Supplement: Supplementary file 7 — Source Data for Figure 3 [file EMBJ-42-e112767-s006.zip › FIGURE_3.zip!/FIGURE 3/3C/Annotations 3C.pdf]

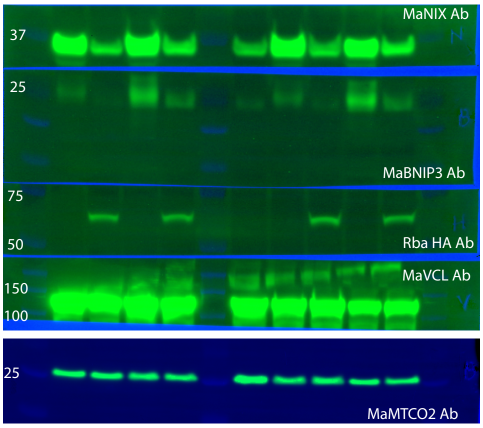
MaNIX Ab
37
25
MaBNIP3 Ab
75
50
Rba HA Ab
MaVCL Ab
150
100
25
MaMTCO2 Ab

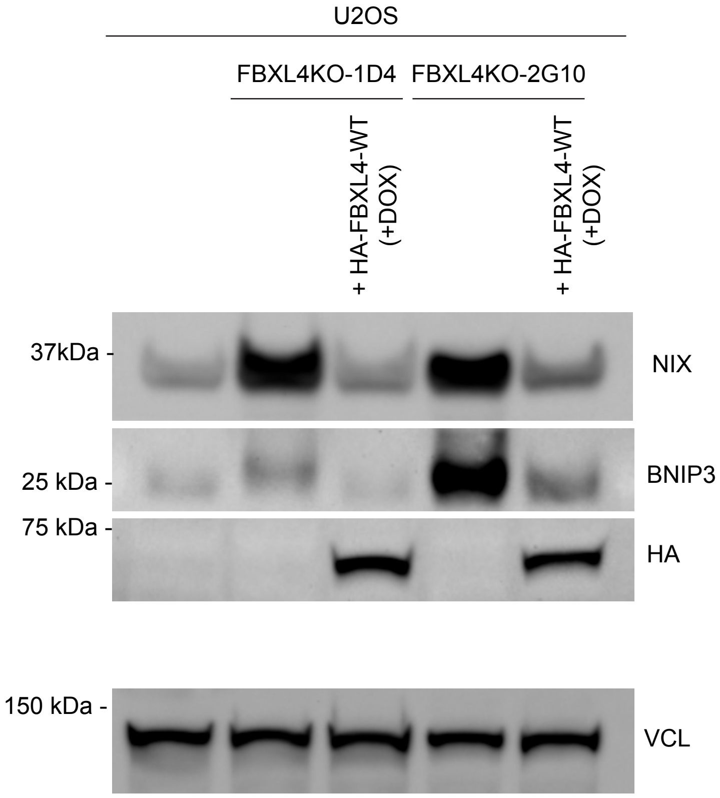
U2OS
FBXL4KO-1D4
FBXL4KO-2G10
+ HA-FBXL4-WT (+DOX)
+ HA-FBXL4-WT (+DOX)
37kDa -
NIX
25 kDa -
BNIP3
75 kDa -
HA
150 kDa -
VCL

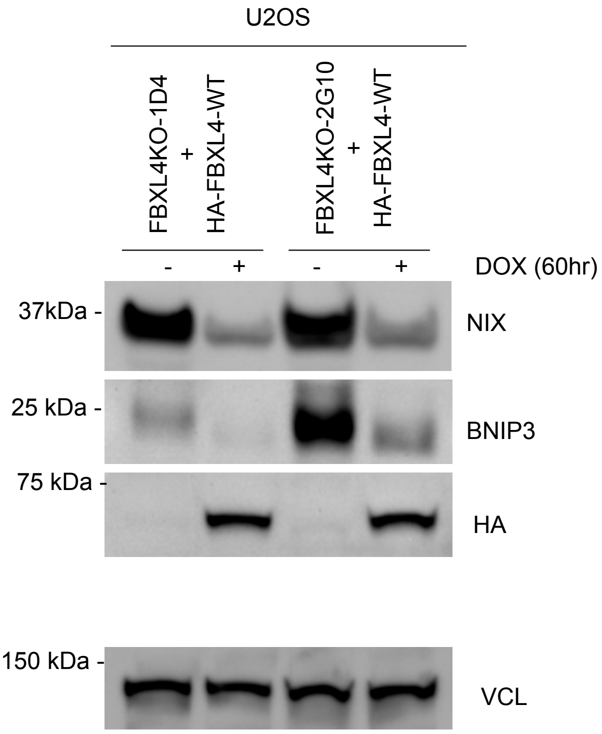
U2OS
FBXL4KO-1D4 + HA-FBXL4-WT
FBXL4KO-2G10 + HA-FBXL4-WT
- + - +
DOX (60hr)
37kDa -
NIX
25 kDa -
BNIP3
75 kDa -
HA
150 kDa -
VCL
