# Supplementary material for: FBXL4 suppresses mitophagy by restricting the accumulation of NIX and BNIP3 mitophagy receptors
Source: EMBO J. 2023 May 10;42(13):e112767. doi: 10.15252/embj.2022112767 (PMC10308361; doi:10.15252/embj.2022112767)
Supplement: Supplementary file 7 — Source Data for Figure 3 [file EMBJ-42-e112767-s006.zip › Figure_3_Images.zip!/Figure 3 Images /3G/Annotations 3G.pdf]

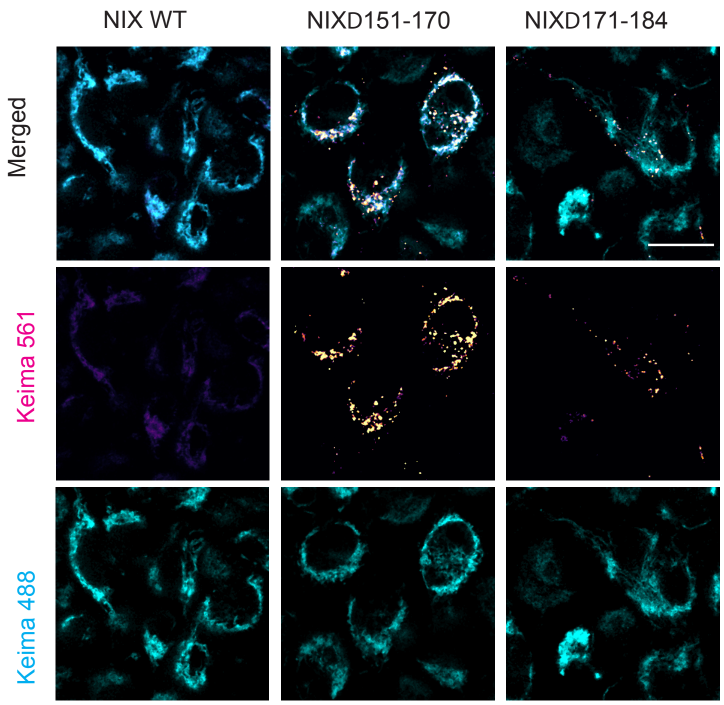
NIX WT
NIXD151-170
NIXD171-184
Merged
Keima 561
Keima 488

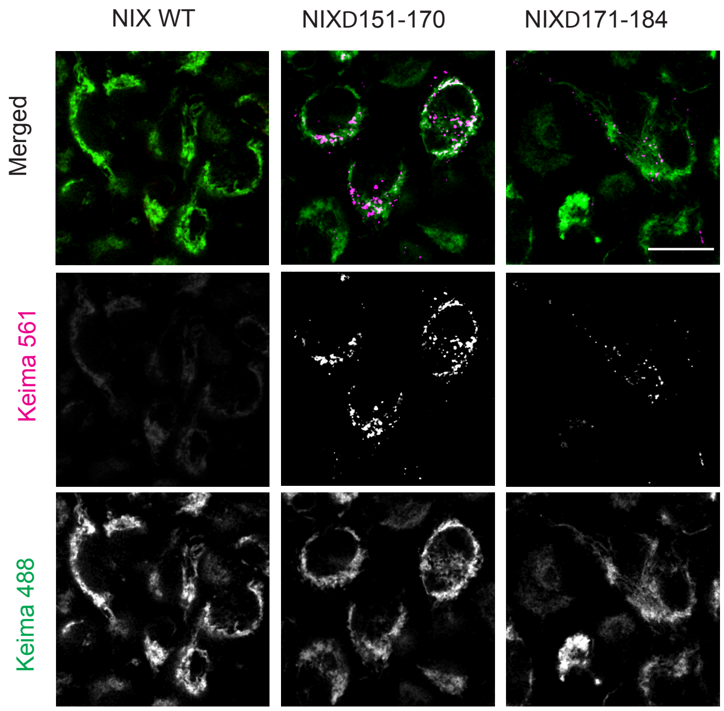
NIX WT
NIXD151-170
NIXD171-184
Merged
Keima 561
Keima 488
