# Supplementary material for: FBXL4 suppresses mitophagy by restricting the accumulation of NIX and BNIP3 mitophagy receptors
Source: EMBO J. 2023 May 10;42(13):e112767. doi: 10.15252/embj.2022112767 (PMC10308361; doi:10.15252/embj.2022112767)
Supplement: Supplementary file 8 — Source Data for Figure 4 [file EMBJ-42-e112767-s007.zip › FIGURE_4.zip!/FIGURE 4 /4G/4G Annotations.pdf]

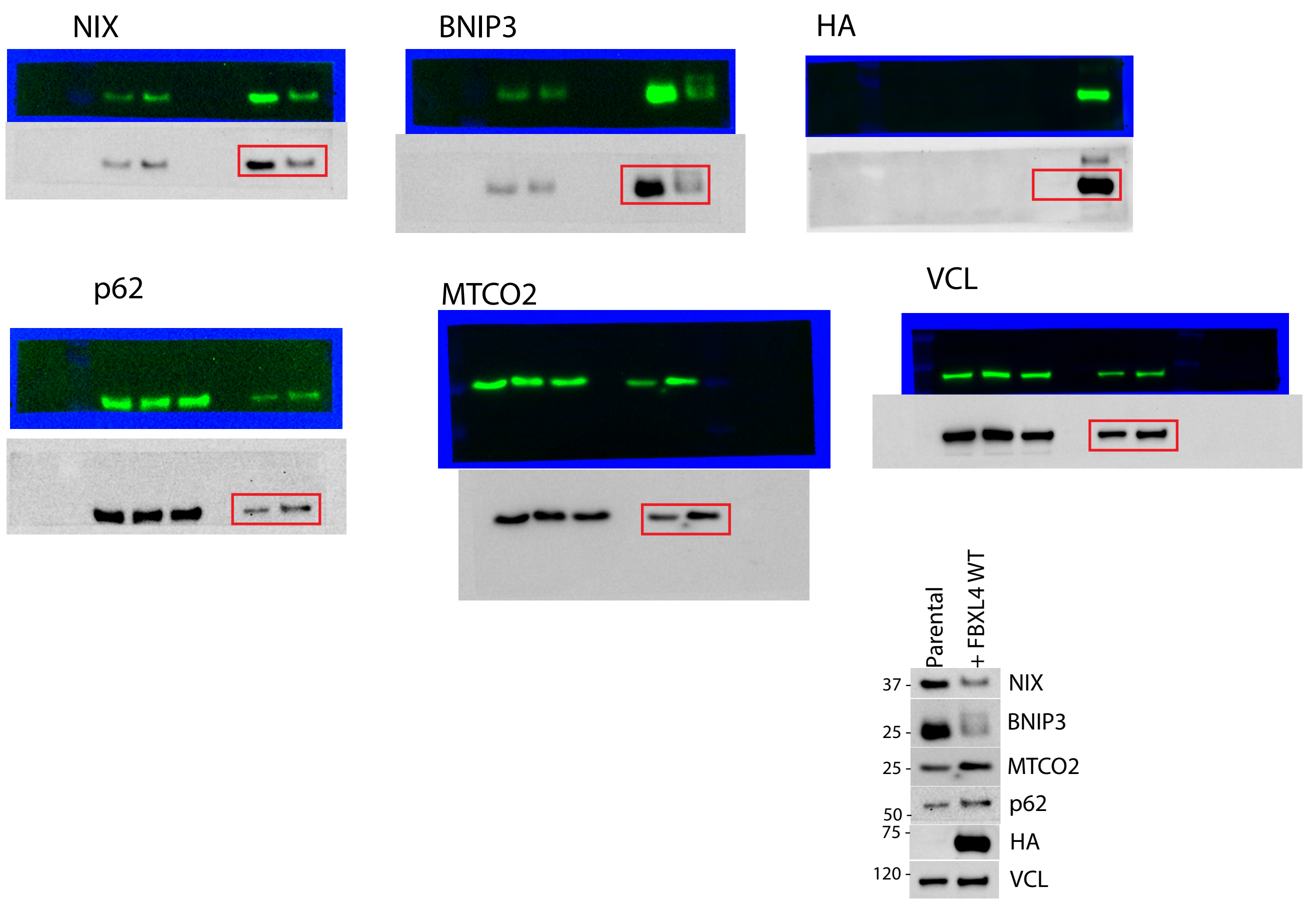
NIX
BNIP3
HA
p62
MTCO2
VCL
Parental
+ FBXL4 WT
37 -
25 -
25 -
50 -
75 -
120 -
NIX
BNIP3
MTCO2
p62
HA
VCL
