# Supplementary material for: FBXL4 suppresses mitophagy by restricting the accumulation of NIX and BNIP3 mitophagy receptors
Source: EMBO J. 2023 May 10;42(13):e112767. doi: 10.15252/embj.2022112767 (PMC10308361; doi:10.15252/embj.2022112767)
Supplement: Supplementary file 8 — Source Data for Figure 4 [file EMBJ-42-e112767-s007.zip › FIGURE_4.zip!/FIGURE 4 /4E/Annotated 4E.pdf]

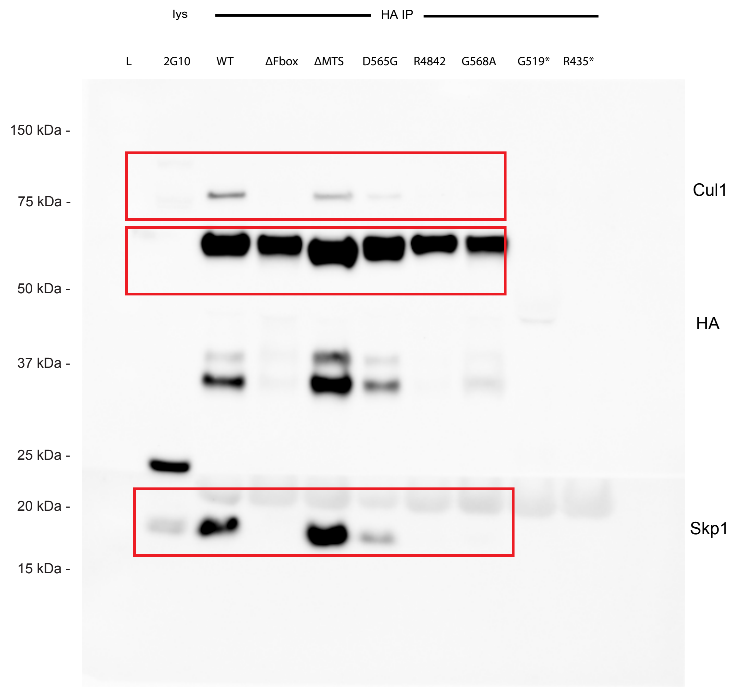
lys
HA IP
L
2G10
WT
ΔFbox
ΔMTS
D565G
R4842
G568A
G519*
R435*
150 kDa -
75 kDa -
50 kDa -
37 kDa -
25 kDa -
20 kDa -
15 kDa -
Cul1
HA
Skp1

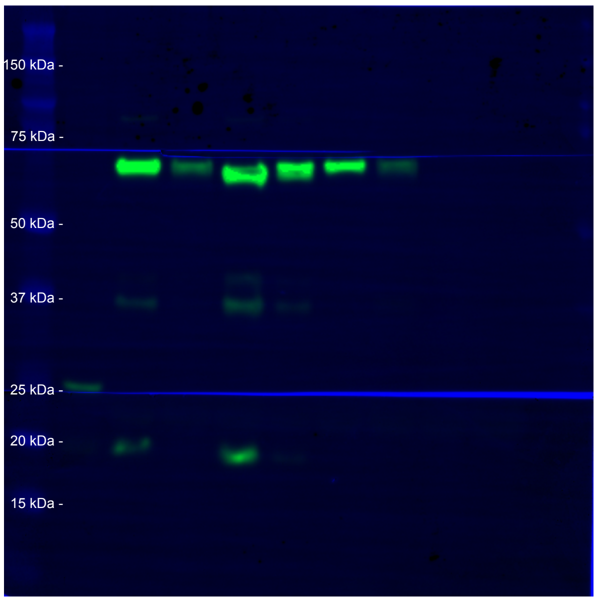
150 kDa -
75 kDa -
50 kDa -
37 kDa -
25 kDa -
20 kDa -
15 kDa -
