# Supplementary material for: FBXL4 suppresses mitophagy by restricting the accumulation of NIX and BNIP3 mitophagy receptors
Source: EMBO J. 2023 May 10;42(13):e112767. doi: 10.15252/embj.2022112767 (PMC10308361; doi:10.15252/embj.2022112767)

VCL M 1:1000

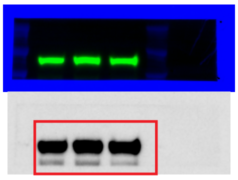

NIX M 1:1000

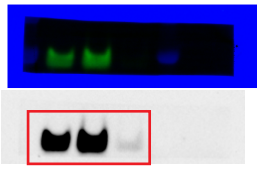

BNIP3 M 1:500

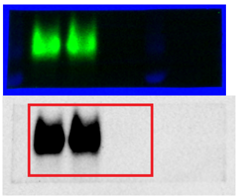

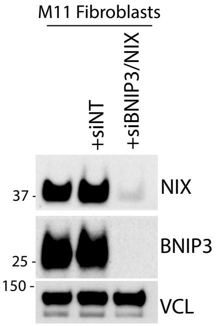

Supplement: Supplementary file 8 — Source Data for Figure 4 [file EMBJ-42-e112767-s007.zip › FIGURE_4.zip!/FIGURE 4 /4H/Annotation 4H.pdf]
