# Supplementary material for: FBXL4 suppresses mitophagy by restricting the accumulation of NIX and BNIP3 mitophagy receptors
Source: EMBO J. 2023 May 10;42(13):e112767. doi: 10.15252/embj.2022112767 (PMC10308361; doi:10.15252/embj.2022112767)
Supplement: Supplementary file 8 — Source Data for Figure 4 [file EMBJ-42-e112767-s007.zip › FIGURE_4.zip!/FIGURE 4 /4C/Annotations - Fig 4C.pdf]

Figure 4C

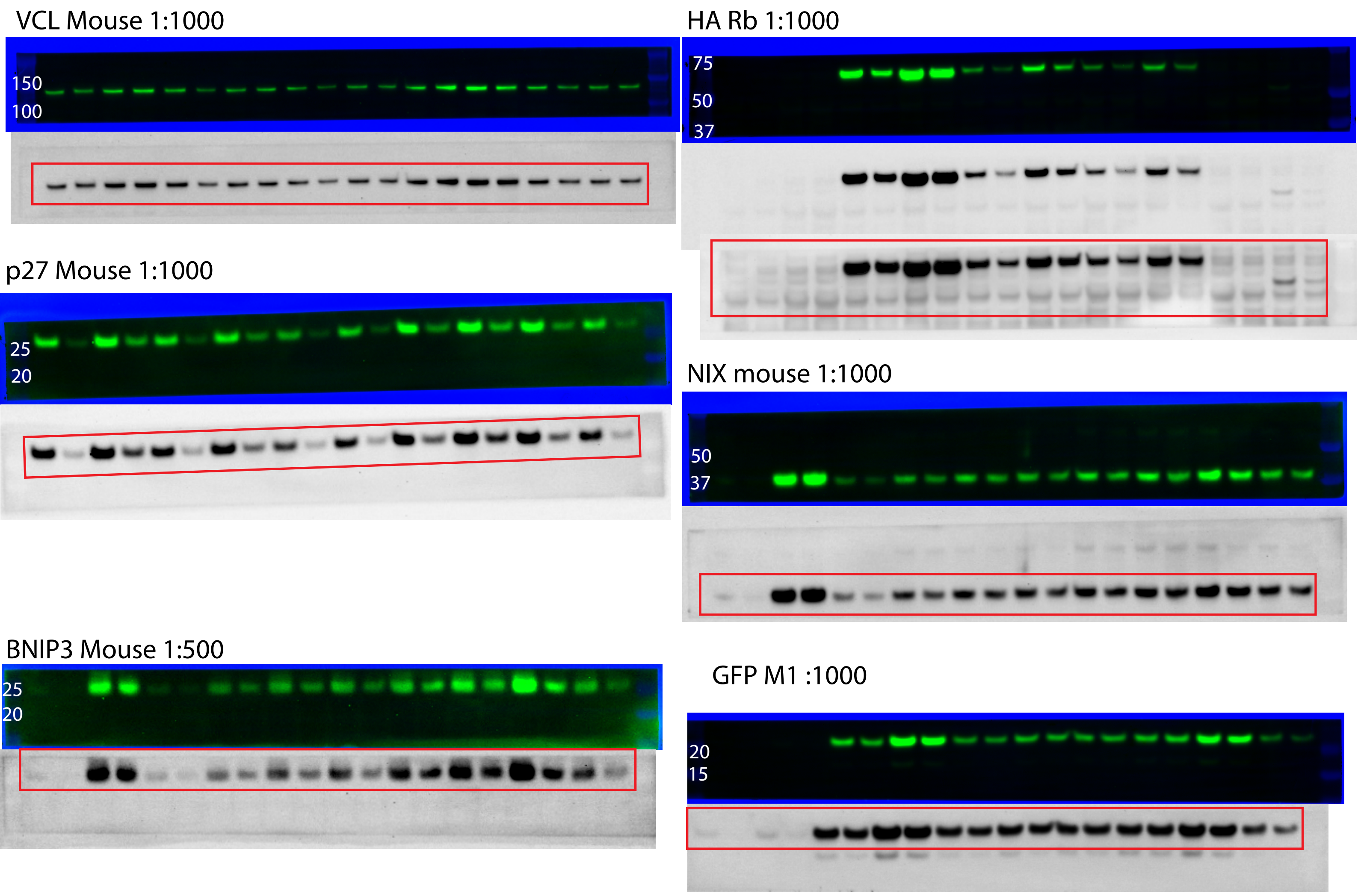
VCL Mouse 1:1000
150
100
HA Rb 1:1000
75
50
37
p27 Mouse 1:1000
25
20
NIX mouse 1:1000
50
37
BNIP3 Mouse 1:500
25
20
GFP M1 :1000
20
15

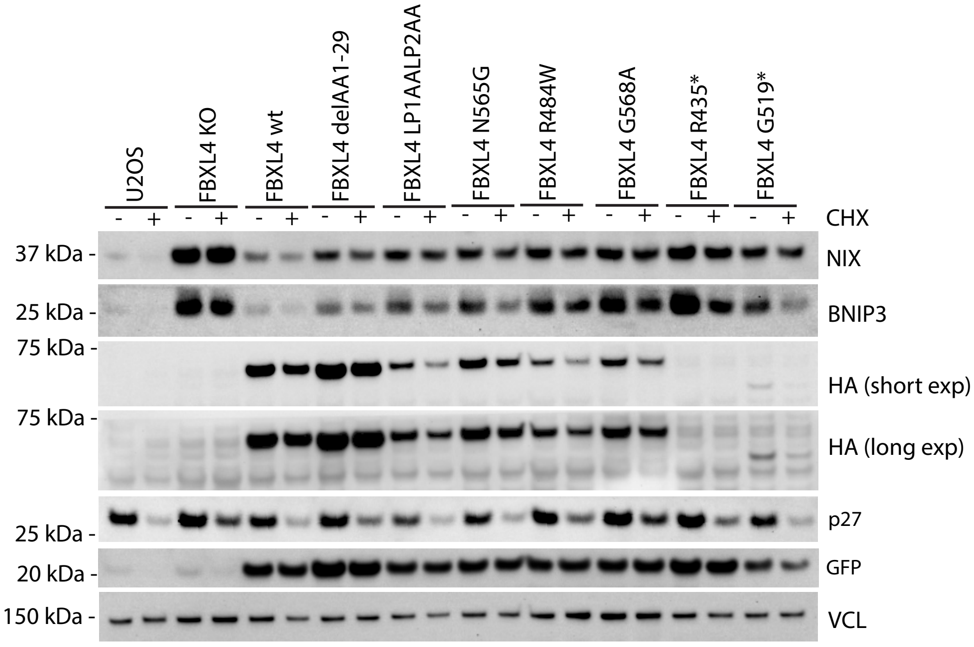
U2OS
FBXL4 KO
FBXL4 wt
FBXL4 delAA1-29
FBXL4 LP1AALP2AA
FBXL4 N565G
FBXL4 R484W
FBXL4 G568A
FBXL4 R435*
FBXL4 G519*
- + - + - + - + - + - + - + - + - + - +
CHX
37 kDa -
NIX
25 kDa -
BNIP3
75 kDa -
HA (short exp)
75 kDa -
HA (long exp)
25 kDa -
p27
20 kDa -
GFP
150 kDa -
VCL
